# Vacancy‐Suppressed Garnet Electrolytes for Durable Solid‐State Batteries

**DOI:** 10.1002/advs.202522562

**Published:** 2026-03-03

**Authors:** Seokjae Hong, Kwang Ho Shin, Kyoung Sun Kim, Jinhwan Kim, Jaegi Lee, Byung‐Gun Park, Won G. Hong, In Hye Kwak, Kyubin Shim, Young‐Sang Yu, Ju Young Kim, Young Hwa Jung, Min Wook Pin, Hyeon‐Jong Lee, Hojoon Kim, Dong‐Hwa Seo, Hosun Shin, Dongju Lee, Seung‐Ho Yu, Sung‐Kyun Jung, Hyungsub Kim

**Affiliations:** ^1^ Neutron Science Division Korea Atomic Energy Research Institute (KAERI) Yuseong‐gu Daejeon Republic of Korea; ^2^ Department of Chemical and Biological Engineering Korea University Seongbuk‐gu Seoul Republic of Korea; ^3^ Division of Chemical and Material Metrology‐Emerging Material Metrology Group Korea Research Institute of Standard and Science (KRISS) Yuseong‐gu Daejeon Republic of Korea; ^4^ Institute For Rechargeable Battery Innovations Research Seoul National University (SNU) Seoul Republic of Korea; ^5^ HANARO Utilization Division Korea Atomic Energy Research Institute (KAERI) Yuseong‐gu Daejeon Republic of Korea; ^6^ Strategic Research Center for Smart Battery Korea Basic Science Institute (KBSI) Yuseong‐gu Daejeon Republic of Korea; ^7^ Department of Physics Chungbuk National University Cheongju Chungbuk Republic of Korea; ^8^ Smart Materials Research Section Electronics and Telecommunications Research Institute (ETRI) Yuseong‐gu Daejeon Republic of Korea; ^9^ Beamline Division Pohang Accelerator Laboratory (PAL) Pohang Republic of Korea; ^10^ Analysis and Assessment Group Research Institute of Industrial Science and Technology (RIST) Pohang Republic of Korea; ^11^ Department of Materials Science and Engineering Korea Advanced Institute of Science and Technology (KAIST) Daejeon Republic of Korea; ^12^ Department of Applied Measurement Science University of Science and Technology (UST) Yuseong‐gu Daejeon Republic of Korea; ^13^ Department of Urban, Energy, and Environmental Engineering Chungbuk National University Cheongju Chungbuk Republic of Korea; ^14^ Advanced Energy Research Institute Chungbuk National University Cheongju Chungbuk Republic of Korea; ^15^ Research Institute of Advanced Materials (RIAM) Seoul National University (SNU) Seoul Republic of Korea; ^16^ School of Transdisciplinary Innovations Seoul National University (SNU) Seoul Republic of Korea; ^17^ Department of Materials Science and Engineering Seoul National University (SNU) Seoul Republic of Korea

**Keywords:** all‐solid‐state batteries, garnet solid electrolyte, interfacial stability, intrinsic‐vacancy, Li/O‐rich sintering

## Abstract

Cubic‐garnet‐type solid electrolytes (SEs) are promising candidates for all‐solid‐state batteries (ASSBs) due to their high ionic conductivity and stability against lithium metal. However, intrinsic lithium and oxygen vacancies formed during high‐temperature sintering can lead to interfacial metal reduction and phase transitions, ultimately causing short circuits. In this study, we quantified these intrinsic vacancies in Li_6.5_La_3_Zr_1.5_Ta_0.5_O_12_ (LLZTO) and proposed a strategy to achieve vacancy‐suppressed, lithium‐stuffed garnet SEs by simply tuning the lithium content in both the green pellet and bedding powder during sintering. The optimized Li‐stuffed LLZTO exhibited lithium occupancy exceeding 6.5 per formula unit (pfu) and oxygen‐vacancy concentrations below 0.02 pfu, resulting in improved chemical stability at the electrolyte–electrode interface and enhanced air stability. The garnet electrolyte demonstrated a critical current density of 1.00 mA cm^−2^ at 30°C and 1.75 mA cm^−2^ at 60°C, along with stable cycling performance over 3000 h in lithium symmetric cells and 2000 cycles in hybrid full‐cells, demonstrating 90% capacity retention. These findings highlight the pivotal role of intrinsic vacancy control in enhancing the structural and electrochemical integrity of garnet electrolytes, thereby promoting their practical application in ASSBs.

## Graphical abstract

A Li/O‐rich sintering strategy effectively suppresses intrinsic lithium and oxygen vacancies in cubic garnet electrolytes, yielding a dense and stable microstructure. The vacancy‐controlled electrolyte exhibits enhanced interfacial and air stability, enabling long‐term solid‐state battery cycling and providing a general design principle for defect‐regulated solid electrolytes.

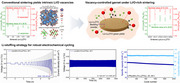

## 1 Introduction

The growing demand for all‐solid‐state batteries (ASSBs), driven by safety concerns associated with flammable liquid electrolytes in Li‐ion batteries, has brought oxide‐based garnets into the spotlight as promising solid‐state ionic conductors [1, 2, 3]. Garnet‐type solid electrolytes (SEs) offer several critical advantages, including high Li‐ion conductivity, negligible electronic conductivity, a wide electrochemical stability window, and chemical compatibility with Li metal [4, 5, 6]. In garnet SEs, the cubic phase, stabilized through aliovalent doping that introduces Li vacancies, exhibits four‐orders‐of‐magnitude higher ionic conductivity (∼10^−3^ S cm^−1^) at room temperature than the tetragonal phase (∼10^−7^ S cm^−1^ at 25°C) [7, 8, 9]. Li vacancies are generally recognized as primary contributors to high ionic conductivity, while O vacancies can also serve as additional pathways facilitating ion transport [10, 11, 12, 13]. However, because Li and O vacancies are typically introduced during high‐temperature sintering through Li_2_O volatilization, they destabilize the cubic phase both at the surface and in the bulk, emphasizing the importance of suppressing their formation to achieve durable and high‐performance garnet SEs.

A number of studies have examined intrinsic Li and O vacancies in garnet SEs using both theoretical calculations and experimental approaches. These vacancies typically appear as Li─O Schottky‐type defects, involving the concurrent removal of Li and O from the crystal lattice [10, 11]. Computational studies have shown that such Schottky defects can lower the Li migration activation energy from values exceeding 1.2 eV to as low as 0.4 eV [12]. Experimental investigations using isotope‐labelled ^18^O and depth‐profiling techniques have further revealed that O vacancies increase the O diffusion coefficient, thereby enhancing Li‐ion mobility [13]. Despite these apparent benefits for ionic transport, Li/O vacancies also create chemically active sites that accelerate degradation. At the Li metal interface, they promote the reduction of metal cations (e.g., Zr) and induce local cubic‐to‐tetragonal phase transition, thereby increasing the interfacial resistance and potentially initiating Li dendrite growth [8, 14, 15, 16, 17]. In addition, garnet SEs are highly susceptible to proton exchange with the ambient atmosphere, which extracts Li and O from the lattice. This process is exacerbated by the presence of Li/O vacancies, leading to the formation of insulating LiOH and Li_2_CO_3_ surface layers and compositional heterogeneities that ultimately degrade the structural stability [18, 19, 20, 21].

To mitigate vacancy‐induced degradation in garnet SEs, many approaches to suppress Li/O vacancies have been explored. The incorporation of excess Li precursors and bedding powders during sintering is widely employed to compensate for Li_2_O volatilization, while oxygen‐rich atmospheres can suppress O deficiency [22, 23, 24, 25]. Kim et al. demonstrated that Li_6.4_La_3_Zr_1.4_Ta_0.6_O_12_ sintered under oxygen‐rich conditions inhibited Li loss while facilitating densification [24]. Ma et al. reported that garnet SEs prepared under an air atmosphere exhibited better mechanical robustness and interfacial stability than those prepared under Ar atmosphere [25]. An entropy‐driven synthetic approach has also been developed to stabilize the cubic phase, with multi‐component doping shifting the Li occupancy toward the theoretical limit of 7 per formula unit (pfu) [26, 27]. These strategies have succeeded in improving the conductivity and phase stability to some extent, yet intrinsic Li/O vacancies remain unavoidable under conventional high‐temperature processing. Consequently, it is essential to elucidate the mechanisms of vacancy formation during garnet synthesis and to establish rational design principles that suppress their detrimental effects.

In this study, we systematically investigate the generation and evolution of Li and O vacancies in cubic Li_6.5_La_3_Zr_1.5_Ta_0.5_O_12_ (LLZTO) prepared by conventional solid‐state synthesis. Neutron diffraction (ND) combined with complementary spectroscopies reveals persistent vacancy formation in the cubic structure, with the Li occupancy remaining below 6.5 pfu and O vacancy concentrations of 0–0.05 pfu even when large excess Li contents (20 wt.%) are introduced. Building on these insights, we introduce a Li/O‐rich sintering protocol that leverages the compositional asymmetry between green pellets and bedding powders. This strategy effectively suppresses Li and O vacancies, achieving Li occupancies above 6.5 pfu and O vacancy concentrations as low as 0–0.02 pfu. The optimized garnet exhibits dense, pore‐free microstructures, enhanced air stability, and reduced interfacial resistance against Li metal. It also delivers excellent long‐term cycling performance, enabling stable operation for over 3000 h in Li symmetric cells and maintaining 90% capacity retention over 2000 cycles in LiFePO_4_ hybrid full‐cells. These results highlight the use of vacancy suppression through compositional tuning as a rational and scalable approach for stabilizing garnet SEs, providing new design guidelines for robust ASSBs.

## 2 Results and Discussion

### 2.1 Intrinsic Atomic Vacancies Formed in Cubic‐Garnet Solid Electrolytes During Conventional Calcination and Sintering Protocols

We synthesized cubic‐garnet‐type LLZTO with varying target excess Li contents (*x *= 0–20 wt.%) using a conventional two‐step solid‐state method (hereafter denoted as Li*x*–LLZTO) to investigate the role of Li during calcination and sintering. The initial calcination temperature was set to 900°C, based on thermogravimetric analysis (TGA), differential scanning calorimetry (DSC), and x‐ray diffraction (XRD) of the precursor mixtures (Figure S1, Supporting Information) [28, 29]. A cubic‐garnet structure was obtained across all Li‐excess compositions at 900°C, while a minor unreacted phase, La_2_O_3_, caused by Li deficiency, was observed in the Li0–LLZTO sample (see Table S1, Supporting Information).

**FIGURE 1:**
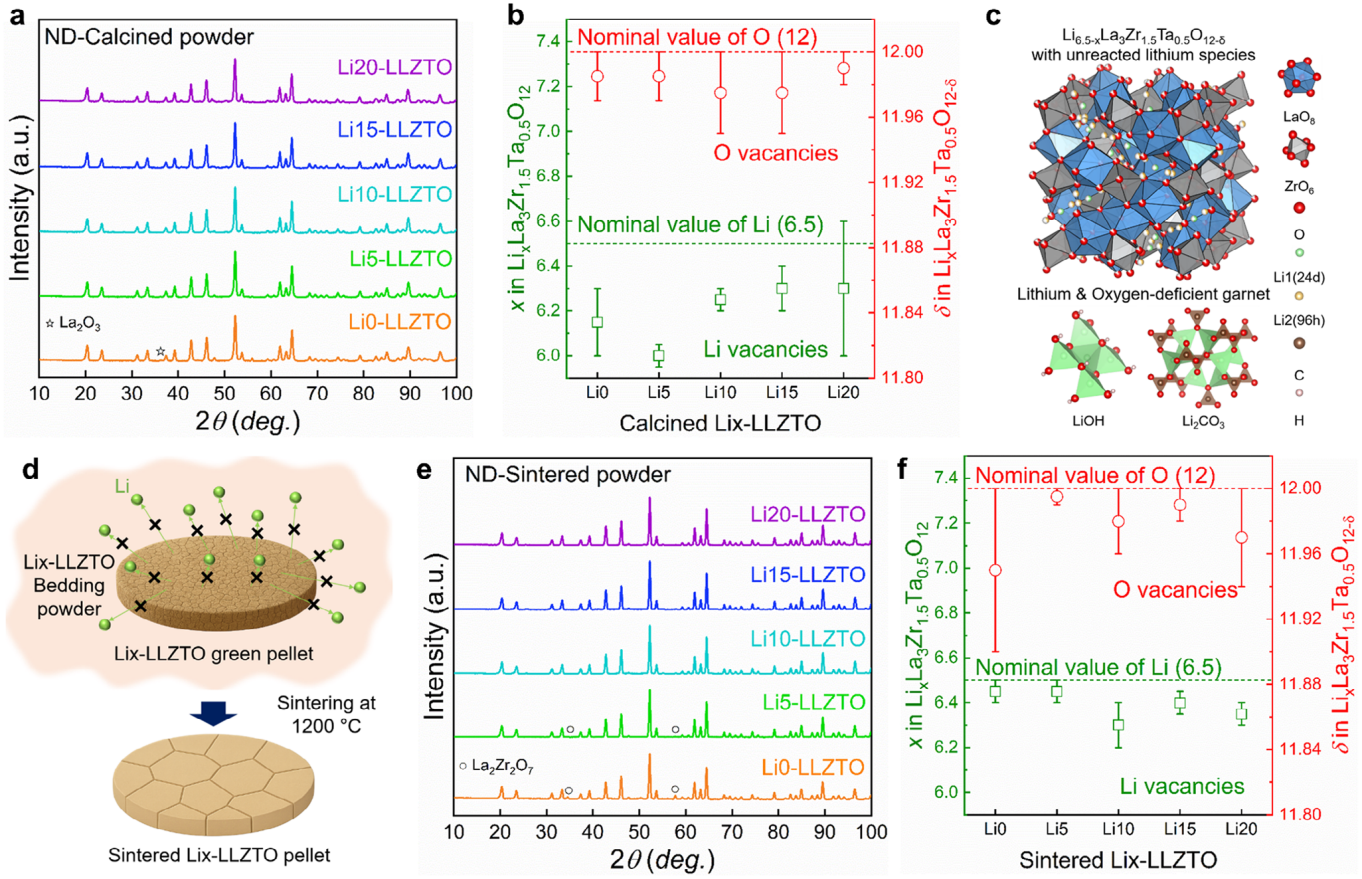
Vacancy evaluation and compositional analysis of LLZTO. (a) ND patterns of calcined Li*x*–LLZTO powders (*x* = 0, 5, 10, 15, and 20). (b) Quantitative analysis of lithium and oxygen contents in calcined Li*x*–LLZTO derived from ND Rietveld refinement (*x* = 0, 5, 10, 15, and 20). (c) Crystal structure model of lithium‐ and oxygen‐deficient cubic‐garnet with unreacted lithium species. (d) Schematic illustration of the sintering process for Li*x*–LLZTO green pellets (*x* = 0, 5, 10, 15, and 20). (e) ND patterns of sintered Li*x*–LLZTO pellets (*x* = 0, 5, 10, 15, and 20). (f) Quantitative analysis of lithium and oxygen contents in sintered Li*x*–LLZTO derived from ND Rietveld refinement (*x* = 0, 5, 10, 15, and 20).

To examine the intrinsic Li vacancies, along with O vacancies in the cubic structure, we performed ND analysis, which is highly sensitive to light elements such as Li and O [30, 31, 32]. As shown in Figure 1a, all the ND peaks matched the cubic‐garnet phase, consistent with the XRD results. However, as depicted in Figure 1b, the Li contents in all the samples was found to be below the nominal value of 6.5 pfu, indicating Li deficiency in the crystal structure. This deficiency is attributed to the volatilization of Li_2_O during high‐temperature calcination, resulting in partial Schottky‐type defects involving Li and O vacancies [10, 11, 12, 25, 31]. To account for charge compensation, a structural model incorporating oxygen defects (Li_6.5−_ *_x_* La_3_Zr_1.5_Ta_0.5_O_12−δ_) was used for Rietveld refinement of the ND data, revealing the presence of approximately 0.02–0.05 O vacancies in the calcined powders. A detailed analysis of the Li and O contents in the cubic structure is provided in Figures S2 and S3 (Supporting Information) and the corresponding note. Although minor variations in the crystal structure, including the lattice parameters and M─O bond lengths, were observed among the Li*x*–LLZTO samples (Figure S4 and Table S2, Supporting Information), residual Li species such as LiOH and Li_2_CO_3_ were clearly identified, as confirmed by titration and O *K*‐edge x‐ray absorption spectroscopy (XAS) (Figure S5, Supporting Information) [33, 34]. The quantities of these residual Li compounds increased proportionally with the target Li contents. Based on these findings, the calcined Li*x*–LLZTO powder can be described as a composite of Li_6.5−x_La_3_Zr_1.5_Ta_0.5_O_12−δ_ and unreacted Li species, as illustrated in Figure 1c.

The influence of excess Li on the formation of intrinsic vacancies was further examined during high‐temperature sintering. TGA–DSC analysis of calcined Li0– and Li20–LLZTO revealed a weight‐loss step associated with the decomposition of residual Li‐containing species such as LiOH and Li_2_CO_3_ (Figure S6, Supporting Information). To further investigate the thermal behavior and phase evolution, ex situ annealing was conducted at 400°C, 600°C, and 800°C for 1 h (Figure S7, Supporting Information), and the corresponding XRD patterns indicated the phase separation of volatile Li species, along with the formation of Li‐deficient secondary phases such as La_2_O_3_ and ZrO_2_. This phase separation was more pronounced near the pellet surface, considering the limited penetration depth of x‐rays into the pellet (∼10 µm) (see Figure S7a–c, Supporting Information) [35]. To quantitatively assess Li sublimation from both residual Li species and the garnet lattice, the Li content of annealed LLZTO samples prepared without bedding powder was analyzed using ND analysis. The results revealed a systematic depletion of Li from the crystal structure with increasing annealing temperature (Figure S8, Supporting Information). These observations highlight the importance of suppressing Li sublimation during sintering. A common strategy to address this issue involves not only introducing excess Li in the starting composition prior to heat treatment but also covering the green pellets with bedding powder of identical composition during sintering (Figure 1d) [22, 36]. Following this approach, Li*x*–LLZTO pellets (*x* = 0, 5, 10, 15, and 20) were sintered at 1200°C using corresponding bedding powders. Both the relative density and ionic conductivity of the sintered pellets increased with Li content, consistent with previous reports (Figure S9, Supporting Information) [35, 37].

The crystal structure of the sintered samples was analyzed using XRD (Figure S10 and Table S3, Supporting Information). Notably, a Li‐poor pyrochlore phase, La_2_Zr_2_O_7_, was detected at the pellet surface, whereas its presence was absent or significantly reduced in pellets pulverized into powder. This discrepancy is attributed to pronounced Li_2_O volatilization from the pellet surface during high‐temperature sintering. For samples with excess Li contents above 10 wt.%, the La_2_Zr_2_O_7_ phase was absent in the powder XRD patterns of the pulverized pellet, indicating that Li sublimation was partially suppressed in the bulk. To quantitatively analyze the Li and O contents in garnet, ND analysis was conducted (Figure 1e). Note that the Li content in the garnet phase remained at or below 6.5 pfu, and the refinement results confirmed the consistent presence of oxygen defects in the sintered samples (Figure 1f). Interestingly, these findings indicate that even when bedding powders are used and the La_2_Zr_2_O_7_ formation is absent, complete prevention of Li loss is not achieved, as Li_2_O volatilization still occurs to a certain extent. This was evident even in the sintered Li20–LLZTO sample, where the added excess Li did not fully prevent the volatilization, resulting in a final Li content below 6.5 pfu and O vacancies. The detailed structural parameters of the sintered Li*x*–LLZTO samples are listed in Figure S11 and Table S4 (Supporting Information). These findings underscore the limitations of the conventional sintering process and highlight the essential need for a revised synthesis protocol capable of suppressing lithium loss and minimizing oxygen‐defect formation.

### 2.2 Synthesis and Characterization of Li‐Stuffed Cubic‐Garnet Solid Electrolytes Under Li/O‐Rich Atmosphere

**FIGURE 2:**
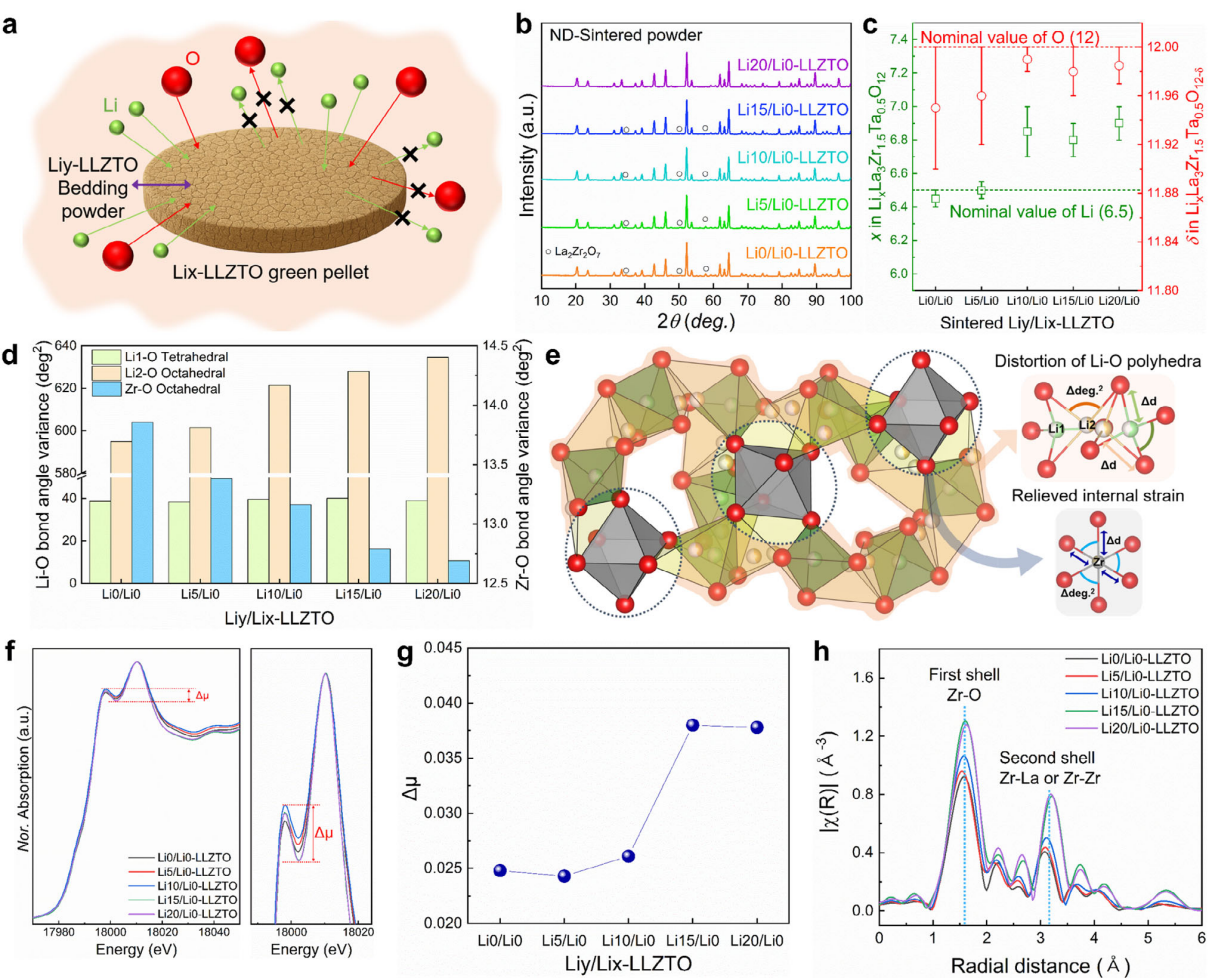
Tailoring the crystal structure of Li‐stuffed garnet through Li/O‐rich atmosphere sintering. (a) Schematic illustration of Li/O‐rich atmosphere sintering protocol. (b) ND patterns of sintered Li*y*/Li0–LLZTO (*y* = 0, 5, 10, 15, and 20). (c) Quantitative analysis of lithium and oxygen contents in sintered Li*y*/Li0–LLZTO derived from ND Rietveld refinement (*y* = 0, 5, 10, 15, and 20). (d) Calculated structural parameters: Li─O and Zr─O bond‐angle variance. (e) Local crystal structures of Zr─O octahedra, Li1─O tetrahedra, and Li2–O octahedra, highlighting Li─O distortion and the associated relief of internal strain. (f) Zr *K‐*edge XAS spectra of Li*y*/Li0–LLZTO (*y* = 0, 5, 10, 15, and 20). (g) Variations in absorption coefficients (Δµ) of ZrO_6_ octahedra in Li*y*/Li0–LLZTO (*y* = 0, 5, 10, 15, and 20). (h) Fourier‐transformed extended x‐ray absorption fine structure spectra at the Zr *K‐*edge of Li*y*/Li0–LLZTO (*y* = 0, 5, 10, 15, and 20).

Building on our understanding of the intrinsic Li and O vacancies formed during synthesis, we intentionally introduced excess Li into the bulk structure by employing Li‐rich bedding powders. Our initial attempts used LiOH or Li_2_CO_3_ as bedding materials to create a Li/O‐rich atmosphere and suppress Li and O volatilization from green pellets during sintering (Figure S12, Supporting Information). However, at the high sintering temperature of 1200°C, these Li compounds melted, leading to deformation and severe extraction of the green pellets. It thus became evident that sintering conditions that limit lithium incorporation beyond the desired level while maintaining compositional parity among La, Zr, and Ta are essential. To address this issue, we devised a strategy in which calcined LLZTO with different excess‐Li levels was used for the green pellet and bedding powder. Guided by titration, XAS, and inductively coupled plasma optical emission sepctroscopy (ICP‐OES) analyses, which consistently showed that the residual Li content increased with increasing excess‐Li levels in the calcined powders (Table S5, Supporting Information, and accompanying text), sintering was conducted using a green pellet (Li*x*–LLZTO) with a lower Li content (*x*) and a bedding powder (Li*y*–LLZTO) with higher Li content (*y*) to control the chemical potential through the compositional asymmetry between them. This configuration, referred to as Li*y*/Li*x*–LLZTO, established a LiOH‐ and Li_2_CO_3_‐enriched environment while maintaining a consistent La, Zr, and Ta composition (Figure 2a). The thermal decomposition of Li compounds generated a Li/O‐rich atmosphere, promoting Li and O incorporation during sintering. The feasibility of this approach was examined using Li*y*/Li*x*–LLZTO powder mixtures, whose thermal and structural evolution is presented in Figures S13–S15 (Supporting Information).

For simplicity, we first prepared Li*y*/Li0–LLZTO samples (*y* = 0–20) sintered at 1200°C for 4 h and examined their crystal structures using ND and XRD (Figure 2b; Figure S16, Supporting Information). It was revealed that high‐temperature sintering induced partial Li_2_O loss from Li0–LLZTO pellets when combined with bedding powders containing up to 15 wt.% excess Li, forming Li‐deficient La_2_Zr_2_O_7_ in pellets pulverized into powder. However, quantitative phase analysis revealed a steady decline of La_2_Zr_2_O_7_ as the Li content in the bedding powder increased (Table S6, Supporting Information). Notably, the XRD patterns of the sintered pellets (Figure S16b, Supporting Information), representing the surface structure, revealed Li‐poor impurities only in samples prepared with Li0– and Li5–LLZTO bedding powders, whereas no secondary phases were detected at Li contents above 10 wt.%. These findings confirm that a Li/O‐rich sintering environment effectively suppresses Li_2_O volatilization, particularly at the pellet surface, and prevents the formation of La_2_Zr_2_O_7_. In parallel, the lattice parameters of both powder and pellet samples systematically decreased with increasing Li content in the bedding powder, suggesting meaningful changes in the garnet framework.

To elucidate these changes, we analyzed the structural parameters using ND and XAS. Rietveld refinement of the ND patterns revealed progressive Li incorporation into the garnet lattice, reaching ∼6.9 pfu without triggering a cubic‐to‐tetragonal transition (Figure 2c). In parallel, the O‐vacancy concentrations decreased, confirming that Li/O‐rich sintering suppresses intrinsic Li and O deficiencies and stabilizes the cubic phase well beyond the nominal 6.5 pfu (Figure S17 and Table S7, Supporting Information). A detailed thermodynamic interpretation of the Li/O incorporation behavior is provided in Figure S17 (Supporting Information), and extended comparison with prior reports on the cubic‐to‐tetragonal transition is summarized in Table S8 (Supporting Information) and corresponding text. A notable feature of this stabilization is the preferential occupation of the Li2 (96 h) site, which alters local Li─Li distances and Li‐loop angles (Table S9, Supporting Information). This site distribution distorts Li2–O octahedra while leaving the Li1–O tetrahedra largely intact (Figure 2d,e), introducing local strain in the Li─O network that is accommodated by relaxation in the adjacent ZrO_6_ framework. Such relaxation reduces Zr─O bond angle disorder and gradually elongates Zr─O bonds (Figure S18, Supporting Information).

Zr *K*‐edge XAS provided complementary evidence for these local structural changes (Figure 2f–h). The increase in the absorption coefficient (Δµ) of the first Zr *K*‐edge peak, along with the intensification of the primary Zr─O peak and moderate enhancements of the Zr─La and higher‐order peak (extending to ∼6 Å) in the Fourier‐transformed extended x‐ray absorption fine structure (EXAFS) spectra, are attributed to lithium enrichment and suppression of O vacancies. These changes within the local and intermediate coordination environments indicate improved short‐ and long‐range structural ordering in the garnet framework [38, 39, 40, 41]. In contrast, Ta L_3_‐edge XAS spectra revealed negligible changes in the oxidation state (Figure S19, Supporting Information). For Li*x*–LLZTO, however, the variations were minimal: the bond lengths remained nearly constant, the Li─Li distances and Li‐loop angles changed only slightly, the Zr *K‐*edge Δµ values were unchanged, and the EXAFS amplitudes and Ta oxidation states remained essentially identical (Figures S20–S23 and Table S10, Supporting Information). The small shifts observed in Rietveld refinement reflect minor Li‐site redistribution between Li1 (24d) and Li2 (96 h) rather than changes in the total Li content (Table S11, Supporting Information).

These observations can be directly linked to the mechanism of the cubic‐to‐tetragonal transition in Li‐stuffed garnets [42, 43, 44]. In tetragonal garnets, ordered Li reduces Li─Li repulsion and relieves Li─O strain but distorts ZrO_6_; in cubic garnets stabilized by aliovalent doping, vacancy‐induced disorder increases Li─O strain but relaxes ZrO_6_ distortion. Our results bridge these regimes: increased Li occupancy at the 96 h site intensifies Li2–O distortions while simultaneously relaxing the ZrO_6_ framework, enhancing the structural symmetry and stabilizing the cubic phase even above 6.5 pfu. In addition to this structural mechanism, we also considered possible charge‐compensation pathways associated with Li enrichment and reduced oxygen deficiency. Defect models allowing partial La and Zr vacancies were incorporated into the Rietveld refinement, as such cation deficiencies can act as charge‐compensating defects to balance the excess positive charge from additional Li insertion. These models yielded improved agreement with the experimental data, supporting the hypothesis that a small fraction of La and Zr vacancies coexist with reduced O vacancies to maintain overall charge neutrality in the Li‐stuffed garnet. The detailed refinement parameters and site occupancies are summarized in Table S12 (Supporting Information).

### 2.3 Effects of Li‐Stuffed Cubic‐Garnet Solid Electrolytes on Electrochemical Performance and Interface Stability

To assess the electrochemical benefits of Li stuffing, we compared Li*y*/Li0–LLZTO (*y* =  0, 5, 10, 15, and 20) pellets with conventionally sintered Li*x*–LLZTO counterparts. As summarized in Figure S24 (Supporting Information), Au|Li*y*/Li0–LLZTO|Au symmetric cells exhibited similar trends in bulk resistance, relative density, and room‐temperature ionic conductivity across both series. The relative density increased steadily with Li content in the bedding powder, and the ionic conductivity reached a high and stable value at *y* ≥ 10 (> 10^−3^ S cm^−1^), requiring a relative density above 90%. In contrast, samples with *y* = 0 and 5 exhibited low density and conductivity, indicating that insufficient Li severely degrades the bulk transport properties and emphasizing the need to decouple the effects of Li enrichment on densification from those on ionic conductivity.

**FIGURE 3:**
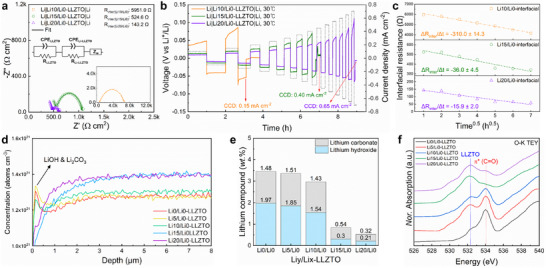
Lithium‐ and oxygen‐vacancy‐dependent interfacial and electrochemical behavior of garnet‐based cells. (a) Nyquist plots for lithium symmetric cells employing Li*y*/Li0–LLZTO (*y* = 10, 15, and 20). The inset displays the equivalent circuit model employed for fitting the impedance spectra together with the extracted resistance components. (b) CCD measurements of lithium symmetric cells, starting at 0.1 mA cm^−2^ with increments of 0.05 mA cm^−2^. (c) Interfacial resistance plot derived from time‐dependent EIS measurements of lithium symmetric cells employing Li*y*/Li0–LLZTO (*y* = 10, 15, and 20). (d) Neutron depth profiling of Li*y*/Li0–LLZTO pellet surface (*y* = 0, 5, 10, 15, and 20). (e) Titration results of sintered Li*y*/Li0–LLZTO (*y* = 0, 5, 10, 15, and 20) powders. (f) O *K‐*edge XAS of sintered Li*y*/Li0–LLZTO (*y* = 0, 5, 10, 15, and 20).

Electrochemical impedance spectroscopy (EIS) on Li|Li*y*/Li0–LLZTO|Li symmetric cells (*y* = 10, 15, and 20) revealed a clear decrease in interfacial resistance with increasing Li content, despite similar ionic conductivity observed in Au symmetric cells (Figure 3a). Critical current density (CCD) measurements followed the same trend, dropping from 0.65 mA cm^−2^ for Li20/Li0–LLZTO to 0.15 mA cm^−2^ for Li10/Li0–LLZTO under identical conditions (Figure 3b). This behavior may be attributed to the reduced interfacial resistance and relative density achieved at *y* ≥ 10. Continuous Li plating/stripping cycling further highlighted the impact of Li stuffing: Li20/Li0–LLZTO maintained low overpotentials and stable cycling for up to 1900 h, whereas Li10/Li0– and Li15/Li0–LLZTO exhibited rapid overpotential growth and short‐circuit failure (Figure S25, Supporting Information). The lithium symmetric cell cycling performance achieved in this study is superior to the cycling stability previously reported for conventionally synthesized lithium‐rich garnet electrolytes in our previous work [35]. In hybrid full‐cells with LiFePO_4_ cathodes, garnets with higher Li content delivered noticeably larger discharge capacities at 0.1 C, confirming that surface Li enrichment and oxygen‐vacancy suppression enhance interfacial stability against Li metal despite unchanged bulk transport properties (Figure S26, Supporting Information). To elucidate the origin of the distinct electrochemical behavior observed among the samples, the physical state of the Li/SE interface after cycling was directly examined by cross‐sectional scanning electron microscope (SEM) analysis (Figure S27, Supporting Information). While Li10/Li0–LLZTO exhibits the formation of a pronounced interfacial gap after cycling, Li15/Li0– and Li20/Li0–LLZTO maintain intimate contact between the lithium metal and the pellet. This preserved interfacial integrity is consistent with their superior electrochemical stability and indicates suppressed interfacial degradation.

To elucidate the origin of the improved interfacial stability, we analyzed pellet surfaces using atomic force microscopy. All the samples exhibited smooth topography and comparable roughness, indicating that morphological factors such as contact area were not the primary cause of resistance reduction (Figure S28, Supporting Information) [45, 46]. Instead, time‐dependent EIS revealed pronounced differences in the degradation kinetics: Li10/Li0–LLZTO showed a rapid decrease in resistance from 5951 to 4159 Ω, whereas that of Li20/Li0–LLZTO gradually decreased from 143 to 57 Ω, consistent with improved reduction stability (Figure 3c). Neutron depth profiling further confirmed pronounced Li enrichment at the surfaces of Li15/Li0– and Li20/Li0–LLZTO, extending up to ∼8 µm without detectable LiOH or Li_2_CO_3_ peaks near the surface (∼1 µm) (Figure 3d). Such Li enrichment suppresses Li vacancies at both the surface and in the bulk, thereby reducing reactive sites that initiate interfacial degradation.

Chemical stability analysis supported this interpretation. Titration measurements and O *K*‐edge XAS revealed systematic reductions in LiOH/Li_2_CO_3_ surface species with increasing Li content (Figure 3e,f) [33, 34]. This enhanced stability originates from the reduced tendency for proton exchange on Li‐ and O‐deficient surfaces, which are otherwise prone to Li attack [19, 20, 21]. Consistent with this interpretation, density functional theory calculations show that reducing the concentration of Li and O vacancies increases the energetic penalty for Li^+^/H^+^ exchange, making proton exchange reactions thermodynamically less favorable (Figure S29, Supporting Information).In line with this finding, electrochemical tests on air‐exposed pellets showed that although all the samples experienced resistance growth after 24 h in ambient air, the increase was smallest for Li20/Li0–LLZTO (Figure S30, Supporting Information). Its CCD after exposure was 0.40 mA cm^−2^, nearly four times higher than that of Li15/Li0–LLZTO, confirming that Li enrichment combined with oxygen‐vacancy suppression effectively mitigates degradation. Despite these improvements, the overall electrochemical performance of Li*y*/Li0–LLZTO remains constrained by the limited densification of the Li0–LLZTO green pellets. Cross‐sectional SEM and energy‐dispersive x‐ray spectroscopy (EDS) revealed closed pores enriched in C and O, even for Li20/Li0–LLZTO, likely reflecting trapped Li_2_CO_3_ formed during sintering under Li‐deficient conditions (Figures S31–S33, Supporting Information). Although both the size and frequency of such pores decreased with increasing Li content, consistent with suppressed Li/O volatilization and vacancy formation, they nonetheless highlight the need for further optimization of pellet densification and interfacial stability. Collectively, these results demonstrate that Li stuffing not only enhances the chemical robustness of garnet electrolytes but also provides a practical route to stabilize Li metal interfaces, provided that bulk microstructural limitations are simultaneously addressed.

### 2.4 Stabilization of Li‐Stuffed Cubic‐Garnet Solid Electrolytes Through Compositional Optimization and Vacancy Suppression

**FIGURE 4:**
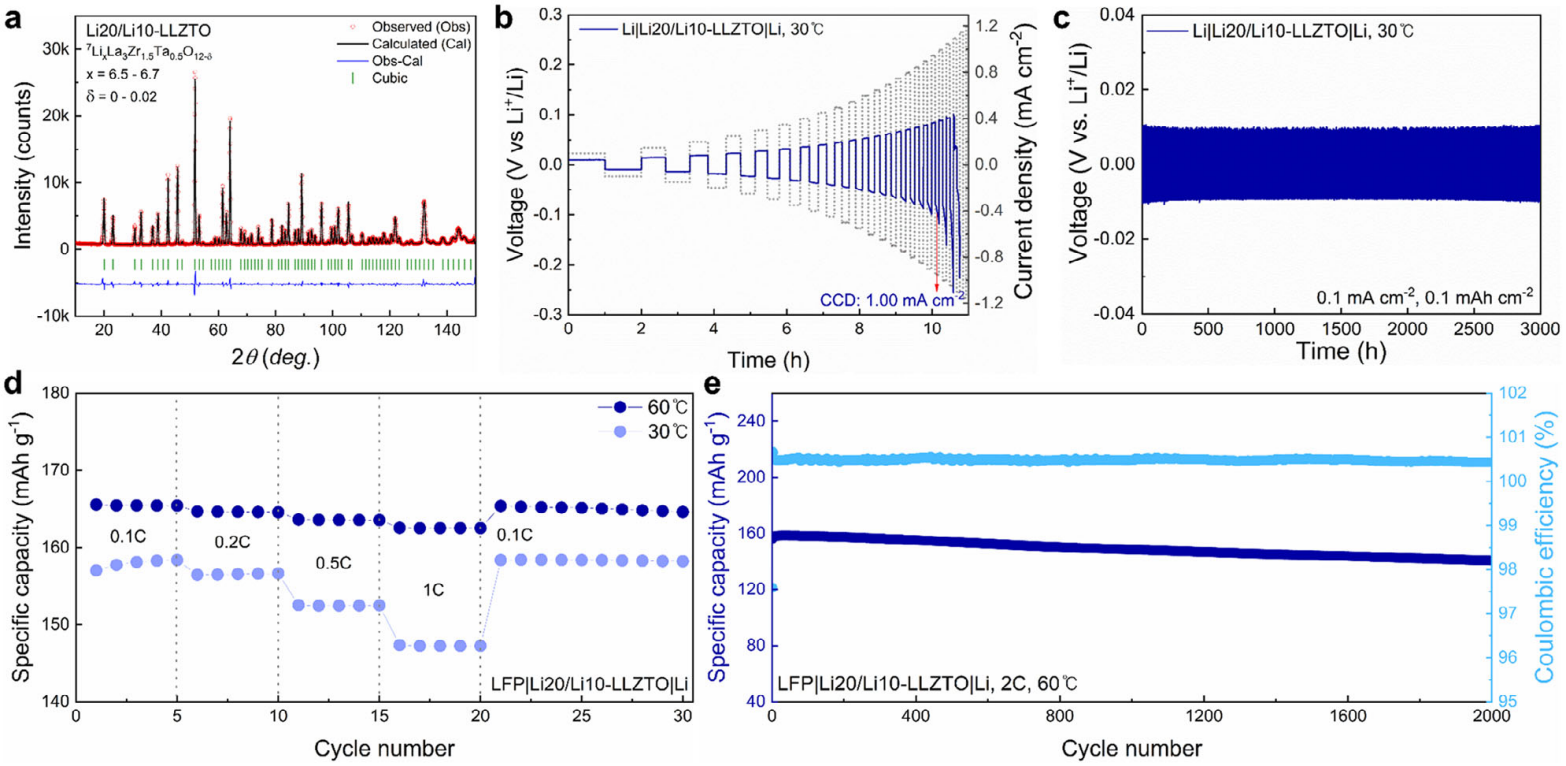
Lithium plating/stripping behavior and hybrid full‐cell performance of compositional modified garnet. (a) ND pattern and Rietveld refinement of Li20/Li10–LLZTO synthesized using a ^7^Li precursor, showing observed (Obs), calculated (Cal), and difference (Obs−Cal) profiles; the vertical tick marks indicate the Bragg reflection positions for the cubic‐garnet phase. (b) CCD measurements of a lithium symmetric cell employing Li20/Li10–LLZTO garnet, starting at 0.1 mA cm^−2^ with an incremental step of 0.05 mA cm^−2^. (c) Voltage profile for a lithium symmetric cell operated at 0.1 mA cm^−2^ and 30°C. (d) Rate performance of the LFP|Li20/Li10–LLZTO|Li hybrid full‐cells cycled at 30°C and 60°C. (e) Discharge capacities of the LFP|Li20/Li10–LLZTO|Li hybrid full‐cell as a function of cycle number operated at 2°C and 60°C.

Building on these insights, we designed a composition combining a 10 wt.% Li‐rich green pellet with a 20 wt.% Li‐rich bedding powder, yielding Li20/Li10–LLZTO. XRD of both pellet surfaces and pulverized pellets into powder confirmed a phase‐pure cubic garnet without detectable Li‐rich or Li‐poor impurities (Figure S34, Supporting Information). ND analysis of ^7^Li‐enriched Li20/Li10–LLZTO, employed to minimize neutron absorption and improve data quality, enabled precise quantification of the stoichiometry (Figure 4a). The Li content in the cubic garnet lattice was refined to 6.5–6.7 pfu, and the oxygen‐vacancy concentration was suppressed to 0–0.02, demonstrating the beneficial effect of Li‐rich bedding powder. The detailed structural parameters are summarized in Figure S35 and Table S13 (Supporting Information). Furthermore, cross‐sectional SEM–EDS revealed a dense, pore‐free microstructure, suggesting strong potential for improved electrochemical performance (Figure S36, Supporting Information).

Electrochemically, Li20/Li10–LLZTO exhibited high ionic conductivity (1.429 mS cm^−1^) with a moderate activation energy (0.3766 eV) in Au symmetric cells (Figure S37, Supporting Information). In Li symmetric cells, Li20/Li10–LLZTO displayed a markedly low initial interfacial resistance (62 Ω) with minimal growth over time. The CCD reached 1.00 mA cm^−2^ at 30°C and 1.75 mA cm^−2^ at 60°C, outperforming Li20/Li0–LLZTO and most previously reported cubic garnet electrolyte, even when compared with extrinsically modified systems (Figure 4b; Figures S38 and S39, Supporting Information). Long‐term cycling at 0.1 mA cm^−2^ and 0.1 mAh cm^−2^ remained stable for over 3000 h without short‐circuiting (Figure 4c). Air‐exposure testing further confirmed its robustness: after 24 h in ambient air, the interfacial resistance increased to 301 Ω, yet the CCD remained 0.60 mA cm^−2^ at 30°C, significantly higher than the value of 0.40 mA cm^−2^ recorded for Li20/Li0–LLZTO under identical conditions (Figure S40, Supporting Information). Hybrid full‐cells with LiFePO_4_ cathodes further validated the performance of Li20/Li10–LLZTO. At 1C, the cell delivered discharge capacities close to the theoretical value (∼162 mAh g^−1^) at 60°C and ∼147 mAh g^−1^ at 30°C (Figure 4d). The capacity retention remained at 90% after 2000 cycles at 2C and 60°C, underscoring the structural and interfacial durability of this optimized garnet (Figure 4e). Overall, these results demonstrate that precise compositional tuning, balancing Li enrichment with oxygen‐vacancy suppression, provides a practical route to achieve phase purity, dense microstructures, low interfacial resistance to Li metal, and long‐term cycling stability in solid‐state‐battery architectures, all without the need for sintering aids or surface coatings.

## 3 Conclusions

In summary, this work demonstrates that intrinsic Li and O vacancies generated during conventional garnet processing critically compromise the structural and electrochemical stability. By systematically investigating vacancy‐formation pathways, we introduced a Li/O‐rich sintering strategy based on the compositional asymmetry between green pellets and bedding powders. This approach effectively suppressed vacancies without inducing a cubic‐to‐tetragonal transition, yielding lithium occupancies above 6.5 pfu and oxygen vacancy concentrations below 0.02. The optimized Li20/Li10–LLZTO exhibited a dense microstructure, stable interfaces against Li metal and air exposure, and long‐term durability in both symmetric cells (3000 h) and hybrid full‐cells (2000 cycles). These findings establish the use of vacancy suppression through compositional tuning as a straightforward and scalable strategy to stabilize garnet electrolytes, providing a practical route toward robust, high‐performance ASSBs.

## 4 Methods

### 4.1 Synthesis of LLZTO With 0–20 wt.% Additional Lithium

Garnet‐type SEs were synthesized through a solid‐state reaction. Precise stoichiometric amounts of LiOH∙H_2_O (99.995%, Aldrich), La_2_O_3_ (99.99%, Aldrich), ZrO_2_ (98%, Aldrich), and Ta_2_O_5_ (99%, Aldrich) were homogenized through planetary ball‐milling at 300 rpm for 3 h to ensure compositional uniformity. The lithium precursor was intentionally added before mixing with the other precursors at specifically 0, 5, 10, 15, and 20 wt.%. The precursor mixture was calcined at 900°C for 6 h, followed by grinding and subsequent re‐ball‐milling at 300 rpm for 3 h. Finally, the resulting powder was sieved to achieve a uniform particle‐size distribution. For the synthesis of garnet using ^7^Li, the same procedure was followed, employing ^7^LiOH∙H_2_O (99.9%, Aldrich) as the lithium precursor.

### 4.2 Sintering Protocol Utilizing Li─O Rich Environment Sintering Through Excess Lithium Content in Garnet

The calcined LLZTO powders with varying excess lithium contents were pelletized under a pressure of 200 MPa, yielding green pellets with a diameter of 10 mm and a mass of 0.5 g. To establish a Li/O‐rich atmosphere during the sintering process, the green pellets were embedded in lithium‐containing mother powders, including precursors (LiOH·H_2_O, Li_2_CO_3_) and Li*x*–LLZTO powders with varying lithium content (*x *= 0, 5, 10, 15, and 20). For all experiments, a constant mass of 4.0 g of mother powder was used, corresponding to a fixed powder‐to‐pellet mass ratio, which strictly maintained across all compositions to ensure reproducibility.

The sintering configuration was designed to ensure complete encapsulation of the pellet by the surrounding powder. A platinum plate was used as the substrate, on which a base layer of mother powder was first spread to define a fixed footprint of approximately 20 × 20 with a thickness of 5 mm. A green pellet was placed at the center of this powder bed. Subsequently, additional bedding powder was added to fully cover the pellet, resulting in a final powder geometry of approximately 20 × 20 × 10 mm^3^ and a symmetric sintering configuration in which the pellet was completely surrounded by the bedding powder during sintering. All samples were sintered at 1200°C for 4 h. A platinum crucible was used in place of an aluminum crucible to prevent unintended aluminum contamination during high‐temperature sintering, except for the lithium‐precursor case. This precaution was essential, as aluminum incorporation from the crucible could alter the lithium composition.

After sintering, the mother powder was completely removed from the pellets. The sintered pellets were then polished with abrasive paper to eliminate and residual powder or surface impurities. The mother powder was not reused for subsequent sintering experiments, as lithium volatilization and Li/O diffusion during high‐temperature processing induce compositional changes in the powder, which would compromise the reproducibility of the sintering environment.

### 4.3 Physical Characterization

The crystal structures of the LLZTO samples were characterized using XRD and ND. XRD data were obtained using an x‐ray diffractometer (Empyrean, Malvern PANalytical) equipped with Cu K*α* radiation (λ = 1.540598 Å). The measurements were performed in the 2*θ* range of 10°–90° with a step size of 0.013 and step time of 150 s. ND data for LLZTO was collected from the high‐resolution powder diffractometer (HRPD) at the HANARO facility at the Korea Atomic Energy Research Institute (KAERI). The measurements were conducted in the 2*θ* range of 0°–160° with a step size of 0.05° using a constant wavelength of 1.834578 Å. TGA–DSC (Labsys Evo TG–DTA, SETARAM) was conducted to 1300°C at a rate of 10°C min^−1^ under synthetic air atmosphere. The calcined Li*x*–LLZTO samples were subjected to ex situ annealing for 1 h at each designated temperature. For ex situ XRD and ND analysis, a 1:1 wt.% mixture powder of Li0– and Li20–LLZTO was heat‐treated at temperatures ranging from 25°C to 1200°C at 100°C intervals for 1 h prior to measurement. The Zr *K‐*, Ta L_3_‐, and O *K‐*edge of LLZTO were characterized using beamlines 10C (Wide XAFS), 8D (XRS), and 10A2 (HR‐PES II) at Pohang Acceleratory Laboratory (PAL). The lithium‐depth distributions in garnet pellets were measured by neutron depth profiling at the HANARO facility at KAERI, which resolves the near‐surface lithium concentration from the energy loss of charged particles generated by the ^6^Li(*n*,*α*)^3^H reaction. LLZTO pellets with a diameter of 10 mm were mounted in a vacuum chamber and analyzed with a silicon detector, and in situ flux normalization was applied. The neutron beam irradiated a circular surface with a diameter of 9 mm, defined by the aperture and collimation, such that the acquired Li depth profiles represent an area‐averaged lithium distribution over the irradiated region. Depth calibration was performed using TRIM (Transport of Ions in Matter) Monte Carlo calculations, parameterized with the measured density of each pellet, providing reliable depth information up to 8 µm. The areal density and absolute Li concentrations were determined by referencing the NIST SRM–2137 standard. The pellet surface images were characterized using SEM (XFlash 6I100, Bruker). The density of each pellet was determined based on Archimedes’ principle, using deionized (DI) water as the liquid medium. The relative density was obtained by calculating the ratio of the measured volume density to the theoretical density. Residual lithium compounds (LiOH and Li_2_CO_3_) were quantified using an acid–base titration method (Eco Titrator, Methohm). The surface morphological properties were measured using AFM (INNOVA–LABRAM HR800, Bruker). The elemental compositions of Li, La, Zr and Ta in the calcined LLZTO powders were quantified by ICP‐OES (Optima 5300 DV, PerkinElmer)

### 4.4 Electrochemical Measurement

For the electrochemical measurement, the sintered pellets were polished in a dry room. EIS measurement was conducted using a potentiostat (SP‐300, Bio‐Logic) to evaluate the ionic conductivity of the SEs and the interfacial resistance of the lithium symmetric cells. Au‐blocking electrodes were deposited on both parallel sides of the polished pellet through sputtering. Subsequently, EIS measurements were conducted using a frequency range of 7 MHz to 100 mHz with an amplitude of 10 mV at 30°C. The ionic conductivities (*σ)* were calculated using the following equation:

(1) $$s=l{\left(RA\right)}^{-1}$$

where *R*, *A*, and *l* represent the resistance, area of the Au‐blocking electrode, and pellet thickness, respectively. The activation energies (*E*_a_) were determined from the behavior of the conductivity as a function of temperature using the following Arrhenius equation:

(2) $$sT=A\exp(-E_{a}/kT)$$

where *A* is the pre‐exponential factor, *k* is the Boltzmann constant, and *T* is the absolute temperature of the sample.

The lithium symmetric cell was prepared by attaching 20‐µm‐thick lithium‐metal foil to both sides of the pellet and applying a CIP of 250 MPa for 5 min in a vacuum environment. The lithium symmetric cells were cycled at a current density of 0.1 mA cm^−2^ with an areal capacity 0.1 mAh cm^−2^ at 30°C (WBCS 3000, WonA Tech). CCD tests of lithium symmetric cells were conducted under an areal capacity 0.1 mAh cm^−2^, with a 0.05 mA cm^−2^ step. The electrodes were fabricated using LFP, polyvinylidene fluoride (PVDF) binder, and Super *P* in a mass ratio of 8:1:1 in *N*‐*methyl*‐2‐pyrrolidone (99.5%, Aldrich). The resulting slurry was spread onto aluminum foil, followed by vacuum drying at 120°C, and then pressed using a roll‐press machine. Coin‐type cells (CR2032, Wellcos) were assembled in an Ar‐filled glovebox by stacking an electrode and the LLZTO pellet (with the counter side attached to Li foil by CIP) and using a 2 M LiFSI in Pyr13FSI ionic liquid to establish an intimate interface and facilitate the formation of an ion‐conduction interface between the electrode and SE. The hybrid full‐cells were charged and discharged within the potential range of 2.5–4.2 V (vs. Li^+^/Li) at 30°C and 60°C.

### 4.5 Density Functional Theory Calculations

First‐principles calculations were performed using spin‐polarized density functional theory (DFT) as implemented in the Vienna Ab initio Simulation Package (VASP) [47, 48, 49, 50]. The generalized gradient approximation (GGA) with the Perdew–Burke–Ernzerhof (PBE) functional was used for exchange‐correlation interactions [51], while the projector augmented wave (PAW) method described ion‐electron interactions. The plane‐wave cutoff energy was set to 650 eV. All DFT relaxations were converged to a 10^−5^ eV for energy and 0.02 eV Å^−^^1^ for force. Other INCAR parameters and *k*‐point grid were generated by MPRelaxSet implemented in pymatgen [52].

Structural models were constructed using supercells containing 8 formula units (Z = 8) based on the stoichiometric Li_56_La_24_Zr_16_O_96_ composition. The tetragonal phase was adopted from the ICSD (CC: 246816) [53]. For the cubic phase, we selected the most stable structure by screening symmetry‐reduced configurations from prior literature [54] using static energy calculations with the universal machine learning interatomic potential model, M3GNet‐MatPES‐PBE‐v2025.1 [55].

To evaluate phase stability with varying compositions, we established baseline supercell: Li_52_La_24_Zr_12_Ta_4_O_96_. Initial models for both tetragonal and cubic phases were generated by substituting Ta at Zr sites and adjusting La/Li atoms were enumerated using the supercell code [56] in the LLZO lattice. From these, 10 most electrostatically favorable configurations were selected for each composition. We then applied a sequential depletion strategy, where stoichiometric two Li atoms and one O atom were iteratively removed to reach a Li content of 46. At each step, 10 electrostatically favorable candidates were generated and fully relaxed using DFT. The tetra‐to‐cubic driving force was determined from the energy difference between the lowest‐energy cubic and tetragonal structures.

Li^+^/H^+^ exchange energies were investigated for cubic phase with the composition Li_52‐2n_La_24_Zr_12_Ta_4_O_96‐n_ (0 ≤ n ≤ 3). From the relaxation process described above, the top 5 energetically stable structures identified via DFT were selected. Based on recent findings that proton exchange occurs preferentially at the energetically less stable octahedral sites [57], the exchange was modeled by substituting a Li atom at an electrostatically unstable 96 h site within the unrelaxed configuration with a hydrogen atom. Finally, the exchange energy was calculated as the total energy difference between the fully relaxed H‐substituted and pristine structures.

## Conflicts of Interest

The authors declare no conflicts of interest.

## Supporting information




**Supporting File**: advs74031‐sup‐0001‐SuppMat.docx.

## Data Availability

The data that support the findings of this study are available in the supplementary material of this article.
