# Supplementary material for: Vacancy‐Suppressed Garnet Electrolytes for Durable Solid‐State Batteries
Source: Adv Sci (Weinh). 2026 Mar 3;13(21):e22562. doi: 10.1002/advs.202522562 (PMC13073312; doi:10.1002/advs.202522562)
Supplement: Supplementary file 1 — Supporting File: advs74031‐sup‐0001‐SuppMat.docx. [file ADVS-13-e22562-s001.docx]

Supporting Information

**Vacancy-Suppressed Garnet Electrolytes for Durable Solid-State Batteries**

Seokjae Hong^1,2,3^, Kwang Ho Shin^4^, Kyoung Sun Kim^1,2^, Jinhwan Kim^5^, Jaegi Lee^5^, Byung-Gun Park^5^, Won G. Hong^6^, In Hye Kwak^6^, Kyubin Shim^6^, Young-Sang Yu^7^, Ju Young Kim^8^, Young Hwa Jung^9^, Min Wook Pin^10^, Hyeon-Jong Lee^11^, Hojoon Kim^11^, Dong-Hwa Seo^11^, Hosun Shin^3,12^, Dongju Lee^13,14^, Seung-Ho Yu^2^, Sung-Kyun Jung^4,15,16,17^*, and Hyungsub Kim^1,14^*

**
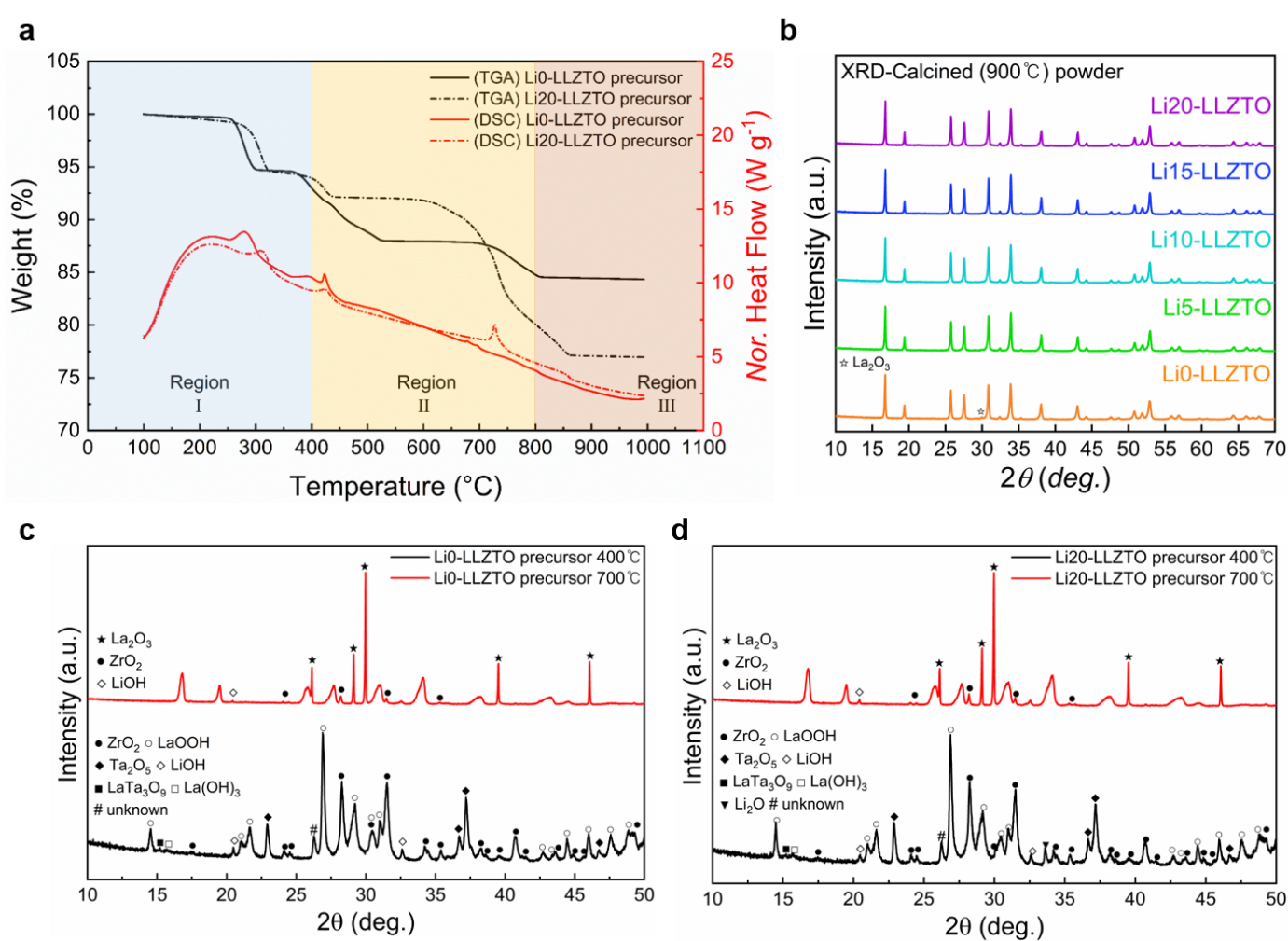
**

**Figure S1**. (a) Thermogravimetric analysis and differential scanning calorimetry (TGA–DSC) curves of Li0– and Li20–LLZTO precursor mixtures. (b) X-ray diffraction (XRD) patterns of Li*x*–LLZTO calcined at 900°C with varying Li contents (*x* = 0, 5, 10, 15, and 20). XRD patterns of Li0–LLZTO (c) and Li20–LLZTO (d) precursor mixtures annealed at 400°C and 700°C.

**Supporting Text for Figure S1**

Cubic-garnet-type Li_6.5_La_3_Zr_1.5_Ta_0.5_O_12_ (LLZTO) with varying target excess Li contents (*x* = 0–20 *wt.*%, Li*x*–LLZTO) was synthesized using stoichiometric amounts of LiOH·H_2_O, La_2_O_3_, ZrO_2_, and Ta_2_O_5_ precursors. The powders were homogenized by planetary ball milling at 300 rpm for 3 h.

*Reaction region I (RT–400°C): Dehydration of lithium precursors and formation of lanthanum hydroxide species*

During low-temperature treatment, the primary process involves removal of adsorbed moisture and dehydration of hydroxyl groups (–OH) from Li precursors. Concurrently, hydroxyl groups react with La_2_O_3_ to yield intermediate lanthanum hydroxides such as LaOOH and La(OH)_3_. After calcination at 400°C, the detected intermediate phases included LaOOH, La(OH)_3_, LaTa_3_O_9_, ZrO_2_, Ta_2_O_5_, and residual LiOH (Figure S1c–d).

*Reaction region II (400°C–800°C): Decomposition of lanthanum hydroxides and initial crystallization*

In this stage, lanthanum hydroxides fully decompose into La_2_O_3_. Simultaneously, LiOH decomposes into Li_2_O, which begins to react with the other precursors. These reactions initiate the crystallization of the cubic garnet phase. After calcination at 700°C, the observed phases were cubic LLZTO, La_2_O_3_, ZrO_2_, and LiOH.

*Reaction region III (800°C–1000°C): Formation and growth of cubic LLZTO*

At elevated temperatures, the remaining La_2_O_3_, ZrO_2_ and LiOH participate in a reaction, enabling complete phase formation. With sufficient thermal energy and time, the precursors are incorporated into the lattice, producing a fully crystallized cubic LLZTO phase (see Figure S1b).

**
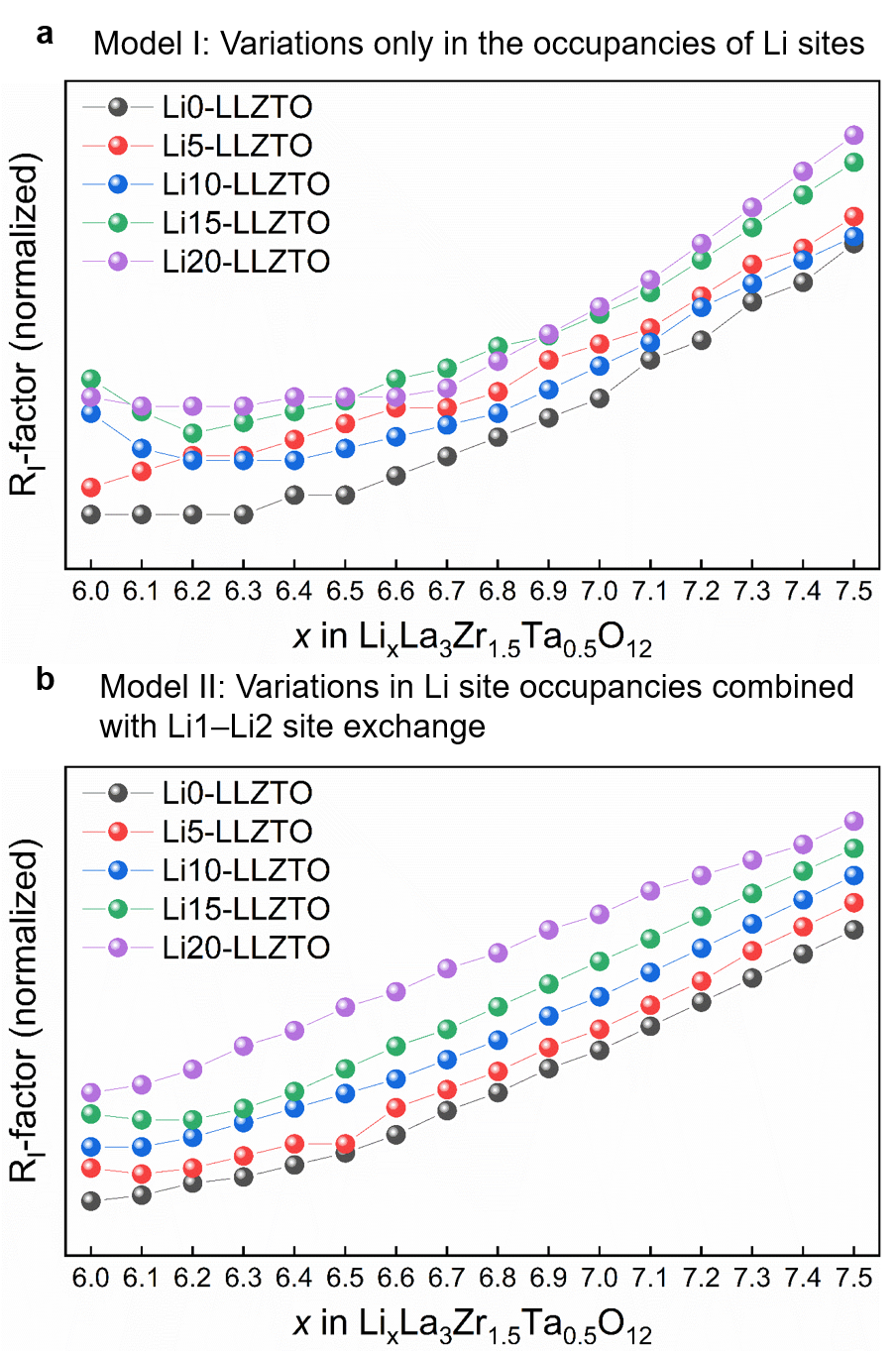
**

**Figure S2**. Quantitative analysis of Li content in the cubic phase of calcined Li*x*–LLZTO (*x* = 0, 5, 10, 15, and 20), derived from Rietveld refinement of neutron diffraction (ND) patterns using two structural models. (a) Model I assumes variations only in the occupancies of the Li1 (24d) and Li2 (96h) sites, whereas (b) Model II incorporates both site occupancies and intersite exchange of Li.


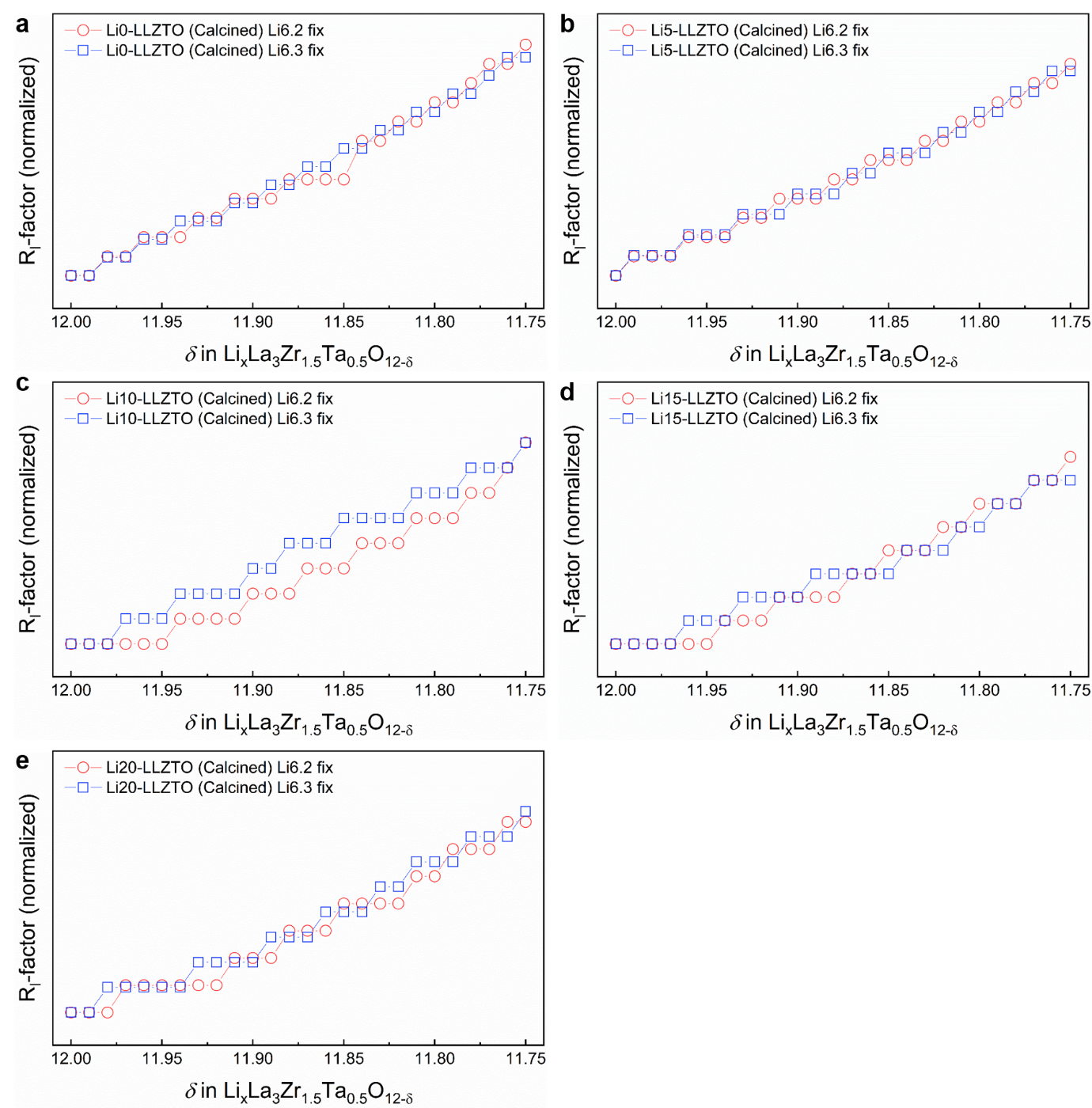


**Figure S3**. Quantitative analysis of oxygen content in the cubic phase of calcined Li*x*–LLZTO (*x* = (a) 0, (b) 5, (c) 10, (d) 15 and (e) 20), derived from Rietveld refinement of ND patterns.

**Supporting Text for Figure S3**

Rietveld refinement of ND patterns was performed to quantitatively evaluate Li and O contents using the Thompson–Cox–Hastings pseudo Voigt function for profile fitting. Several structural models were tested to establish a reliable approach.

For Li quantification, the O sites were initially assumed to be fully occupied. After refining all the parameters unrelated to Li occupancy, the Li-site occupancies were examined by monitoring the variation of the R-factor. Two models were compared: Model I considered only changes in the occupancies of the Li1 (24d) and Li2 (96h) sites, whereas Model II also included possible intersite exchange between Li1 and Li2. Model II did not adequately capture the experimental behavior, whereas Model I provided a better fit; thus, Model I was adopted for further analysis.

Once the Li occupancies were determined, the O vacancies were evaluated with Li fixed at the optimized values. Although the Li contents varied slightly among samples, most clustered around 6.2–6.3 per formula unit (pfu), which was used as the reference composition for oxygen analysis. The oxygen occupancy was then systematically varied to assess its effect on the refinement. To ensure consistency, the R-factor values were normalized for dataset-dependent variations. Thresholds of 0.05 for Li and 0.06 for oxygen were applied as criteria for identifying the most reliable occupancy values. Although the complexity of the analysis limits absolute quantification, the results reliably illustrate the overall trends in Li and oxygen occupancy.


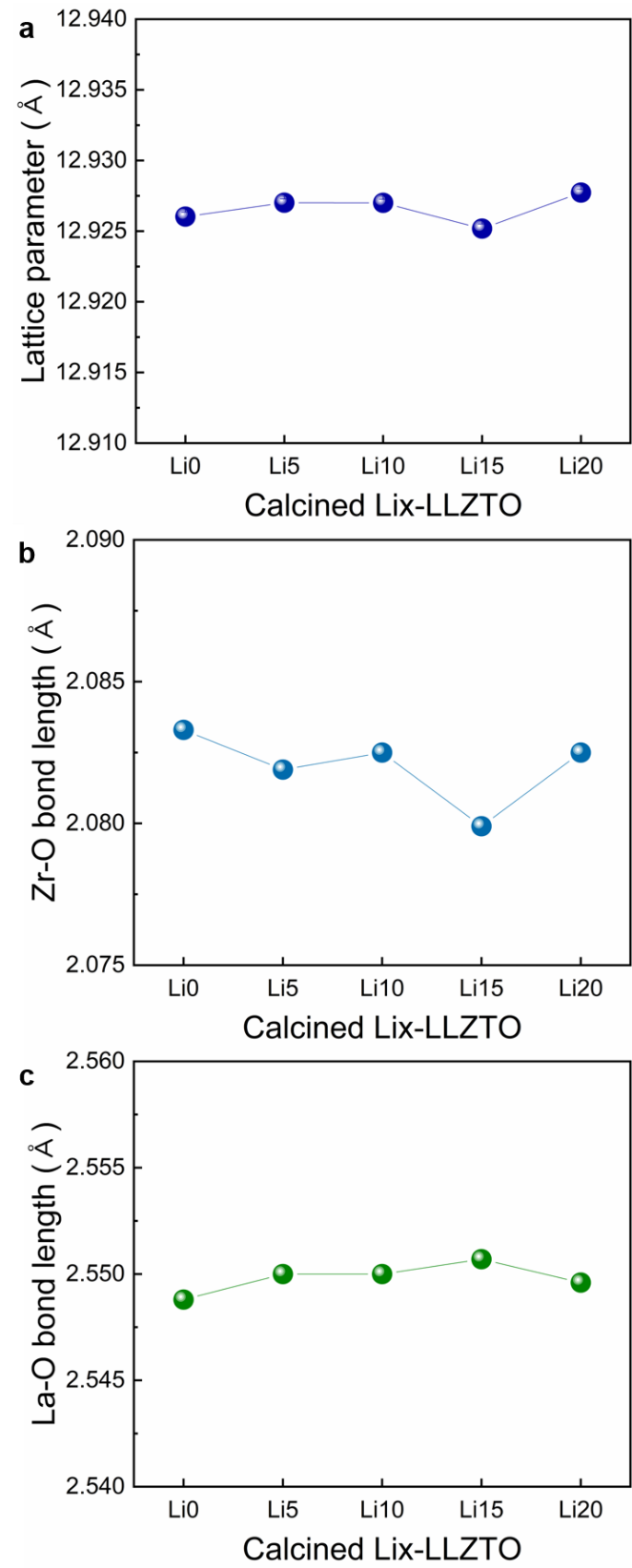


**Figure S4**. Structural parameters of calcined Li*x*–LLZTO (*x* = 0, 5, 10, 15 and 20) derived from ND Rietveld refinement: (a) lattice parameter, (b) Zr–O bond length, and (c) La–O bond length.

**
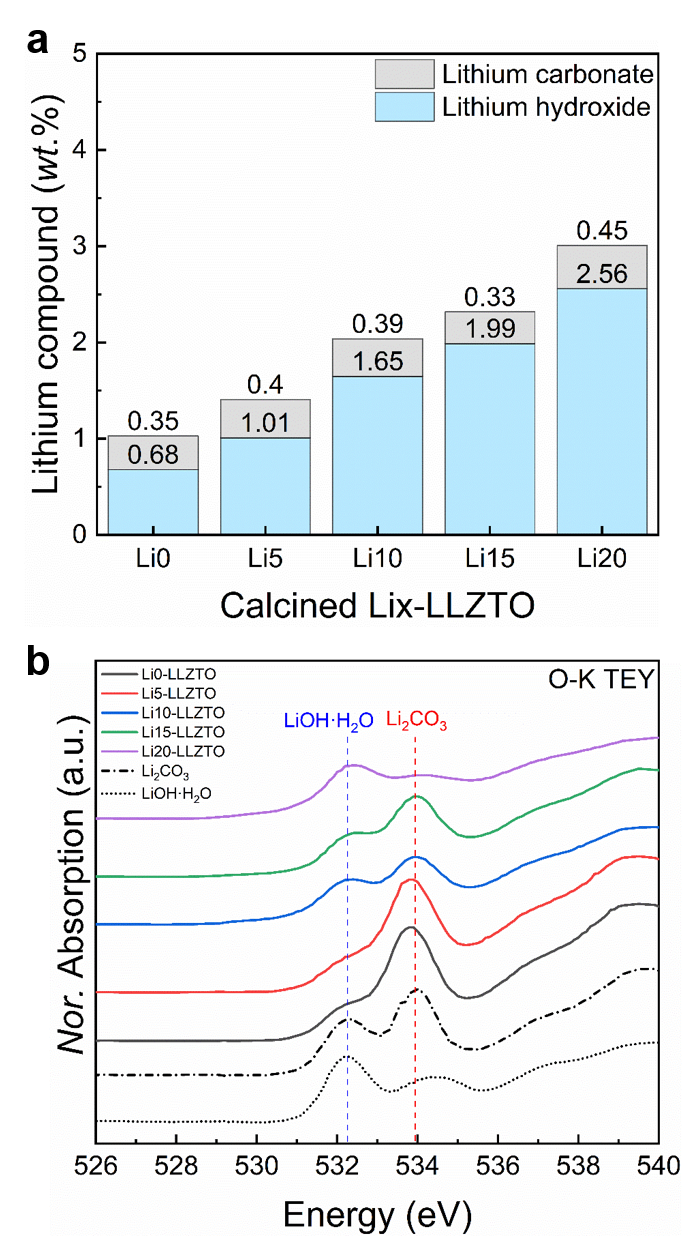
**

**Figure S5**. (a) Titration results of calcined Li*x*–LLZTO powders. (b) O K-edge X-ray absorption spectroscopy (XAS) spectra of calcined Li*x*–LLZTO (*x* = 0, 5, 10, 15, and 20).


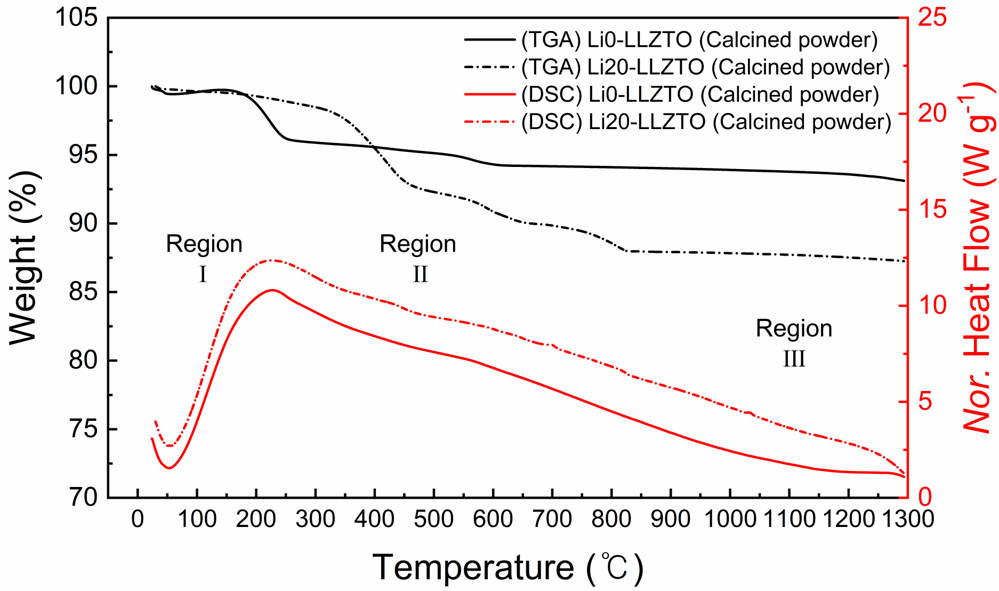


**Figure S6**. TGA–DSC curves of calcined Li0– and Li20–LLZTO upon heating.

**Supporting Text for Figure S6**

The TGA curves of calcined Li*x*–LLZTO (*x* = 0 and 20) show three distinct weight-loss regions, associated with the decomposition of residual Li compounds and subsequent phase evolution.

*Reaction region I (RT–200°C): Dehydration*

The initial weight loss is attributed to the removal of adsorbed moisture from the powder surface.

*Reaction region II (200°C–600°C for Li0–LLZTO; 200°C–800°C for Li20–LLZTO): Decomposition of residual Li compounds*

This region corresponds to the decomposition of LiOH and Li_2_CO_3_ remaining after calcination, leading to Li_2_O evaporation and CO_2_ release. The decomposition profile depends on the initial Li excess; Li20–LLZTO exhibits a broader and higher-temperature weight-loss range than Li0–LLZTO, reflecting the larger amount of residual Li compounds (see Figure S5a).

*Reaction region III (>600°C for Li0–LLZTO; >800°C for Li20–LLZTO): Crystallization and densification*

At this stage, the major decomposition processes are complete, and the material undergoes solid-state reactions that promote cubic-garnet crystallization and densification. Phase purity and structural integrity are largely established during this regime.

**
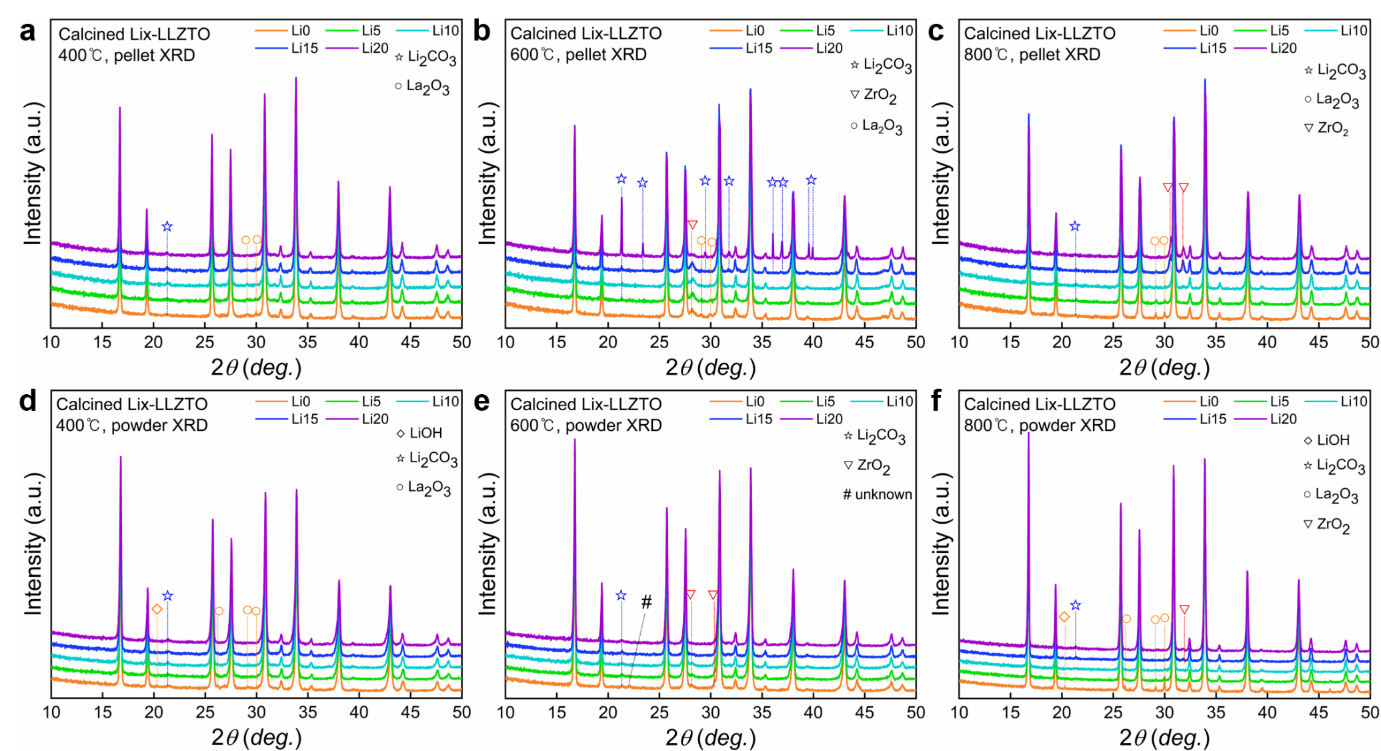
**

**Figure S7**. *Ex situ* XRD patterns of Li*x*–LLZTO pellets (*x* = 0, 5, 10, 15, and 20) annealed at (a) 400°C, (b) 600°C, and (c) 800°C after calcination. *Ex situ* powder XRD patterns of calcined Li*x*–LLZTO annealed at (d) 400°C, (e) 600°C, and (f) 800°C.

**
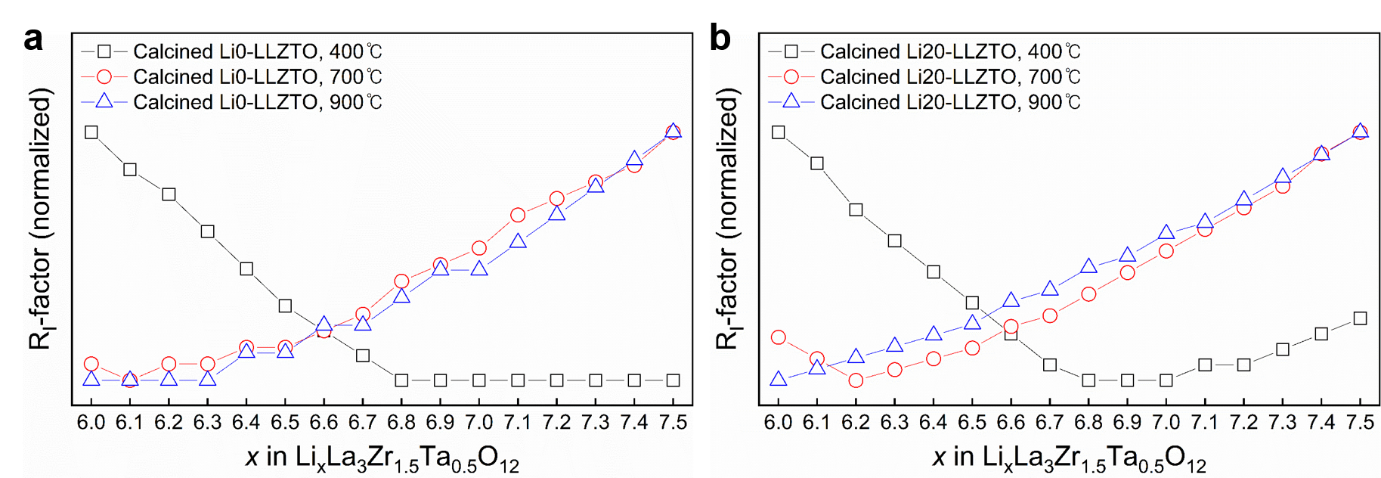
**

**Figure S8**. Quantitative analysis of Li content in cubic phase of calcined (a) Li0–LLZTO and (b) Li20–LLZTO annealed at 400°C, 700°C, and 900°C**.**

**
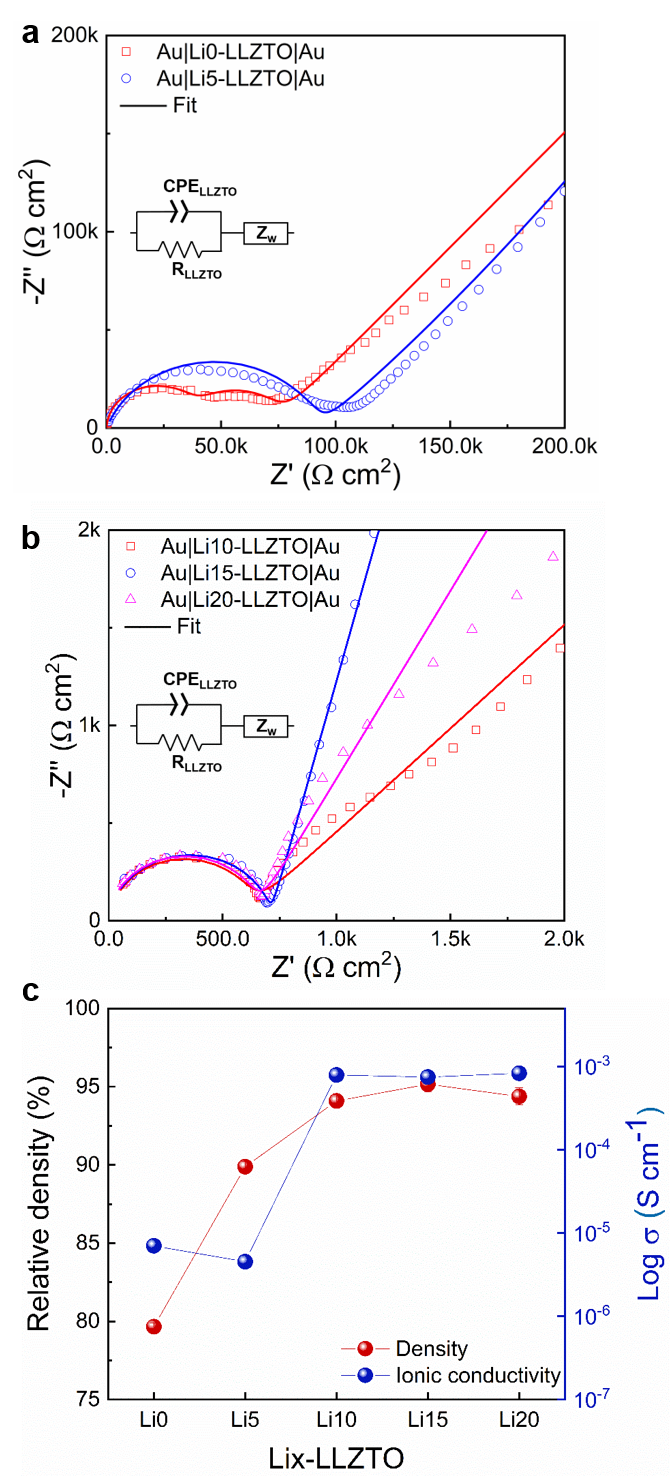
**

**Figure S9**. Nyquist plots of Au|Li*x*–LLZTO|Au cells; (a) *x* = 0 and 5, (b) *x* = 10, 15, and 20. The insets in (a) and (b) display the equivalent circuit model employed for fitting the impedance spectra, together with the extracted resistance components. (c) Relative density and ionic conductivity of Li*x*–LLZTO with *x* = 0, 5, 10, 15, and 20.

**
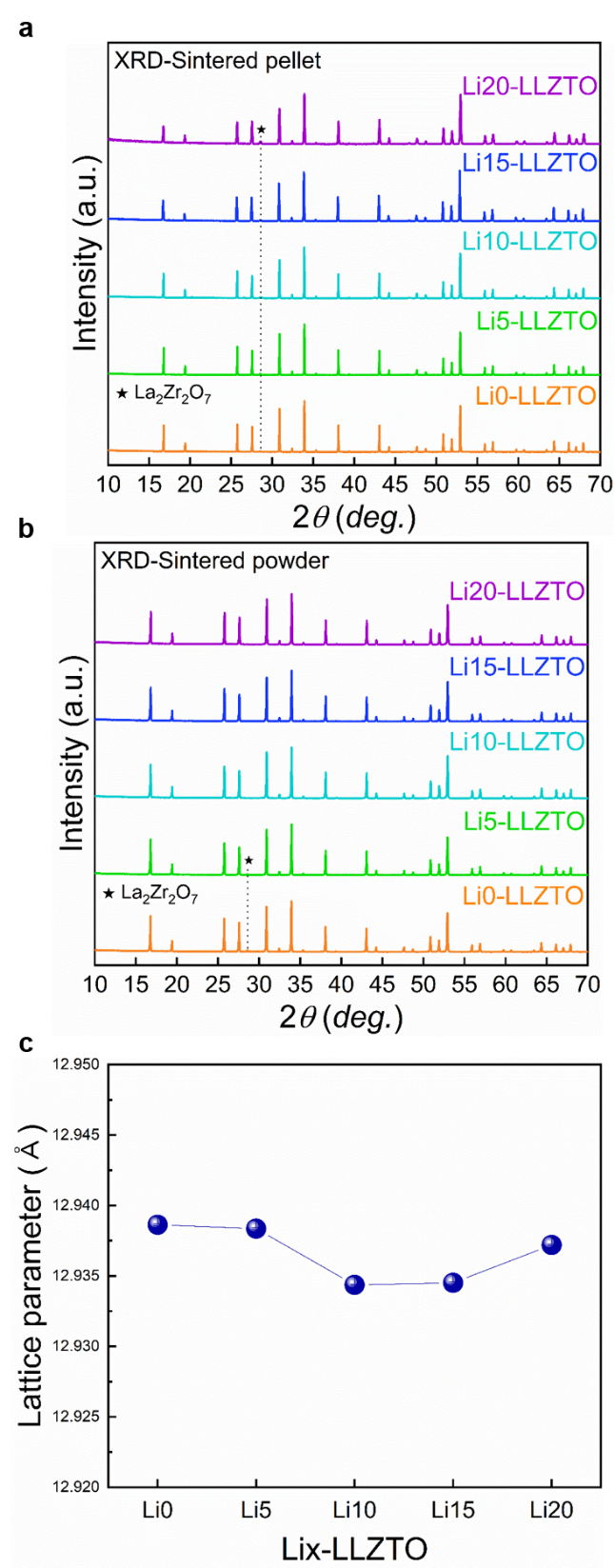
**

**Figure S10**. XRD patterns of sintered Li*x*–LLZTO: (a) intact pellet and (b) pulverized pellet with *x* = 0, 5, 10, 15, and 20. (c) Lattice parameters derived from XRD patterns of pulverized pellet with *x* = 0, 5, 10, 15, and 20.

**
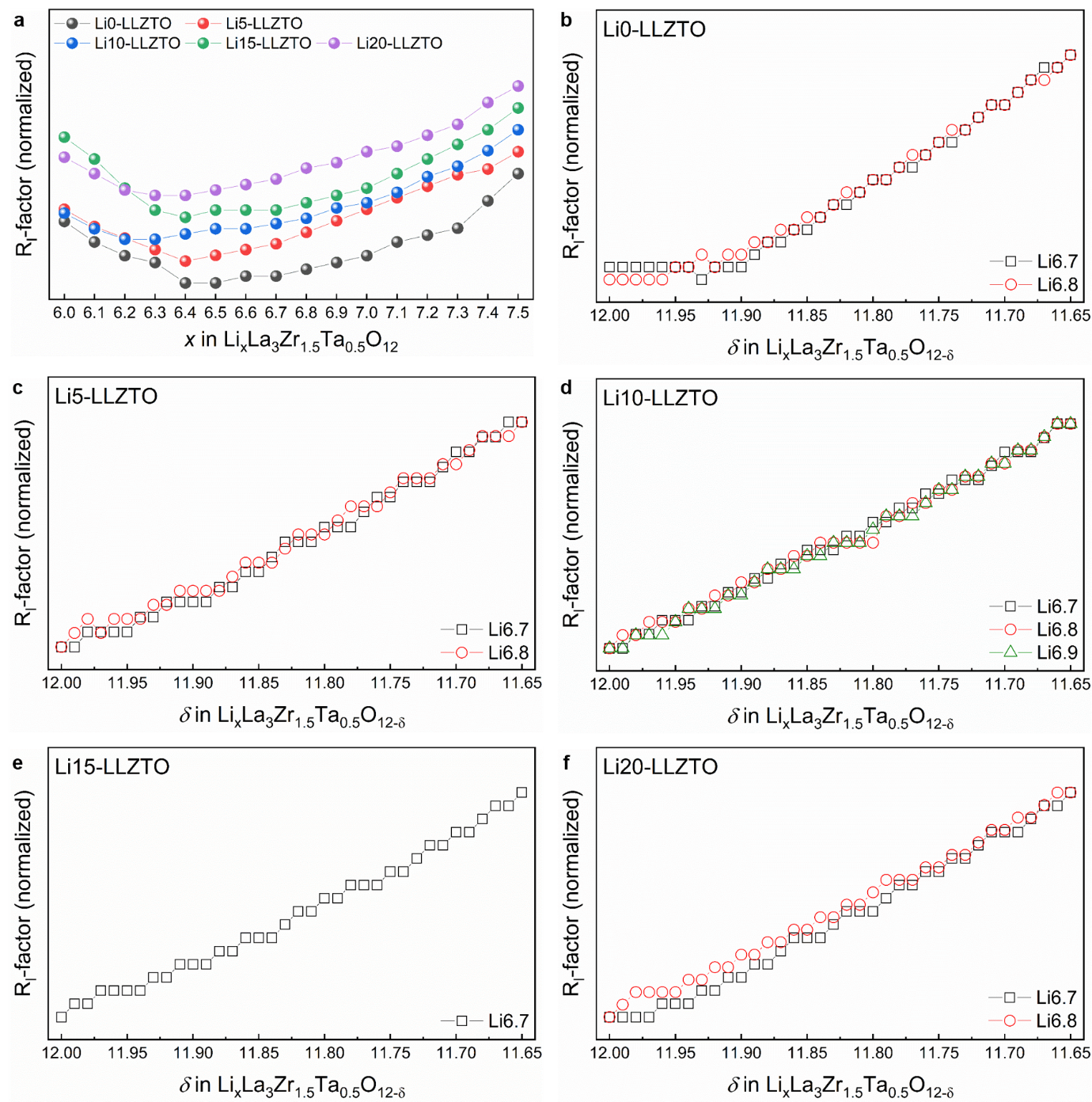
Figure S11**. (a) Quantitative analysis of Li content in the cubic phase of sintered Li*x*–LLZTO with *x* = 0, 5, 10, 15, and 20. Quantitative analysis of oxygen content in the cubic phase of sintered Li*x*–LLZTO: (b) *x* = 0, (c) 5, (d) 10, (e) 15, and (f) 20. All the values were derived from ND analysis.

**Supporting Text for Figure S11**

Lithium and oxygen quantification in sintered Li*x*–LLZTO (*x* = 0, 5, 10, 15, and 20) was performed as described below. For the Li content, the occupancies of the Li1 (24d) and Li2 (96h) sites were systematically varied, and the corresponding R-factors were evaluated after min–max normalization. A threshold of 0.05–0.06 above the minimum R-factor defined the acceptable range for Li quantification. Using these optimized Li occupancies, the oxygen vacancies were then assessed by varying the oxygen occupancy while fixing the Li content. The resulting R-factors were similarly normalized, with a variation of 0.05–0.06 from the minimum taken as the criterion for determining the most reliable oxygen occupancy.

**
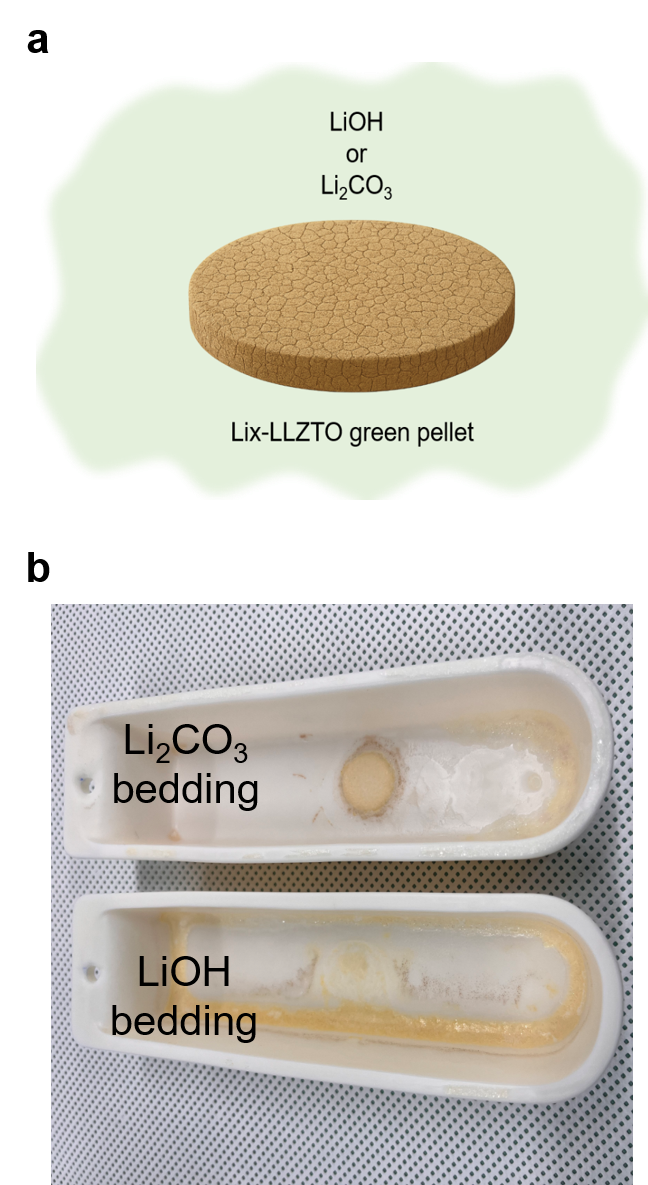
**

**Figure S12**. (a) Schematic illustration of the Li/O rich atmosphere sintering process for Li*x*–LLZTO green pellets using lithium precursors (LiOH and Li_2_CO_3_) as bedding powders. (b) Photograph of the pellets after sintering at 1200°C.

**
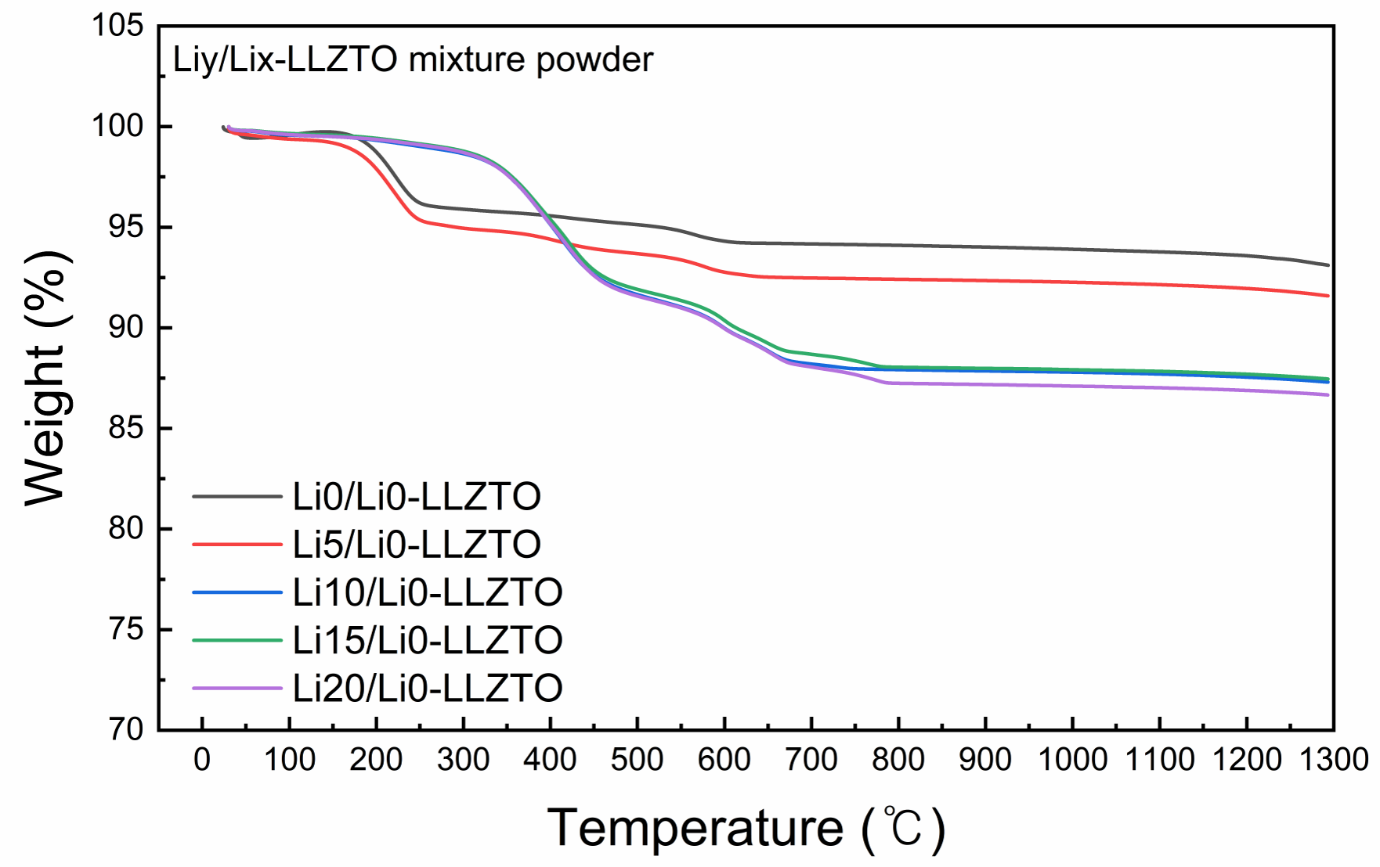
**

**Figure S13**. TGA curves of Li*y*/Li0–LLZTO composite mixtures with *y* = 0, 5, 10, 15, and 20.

**
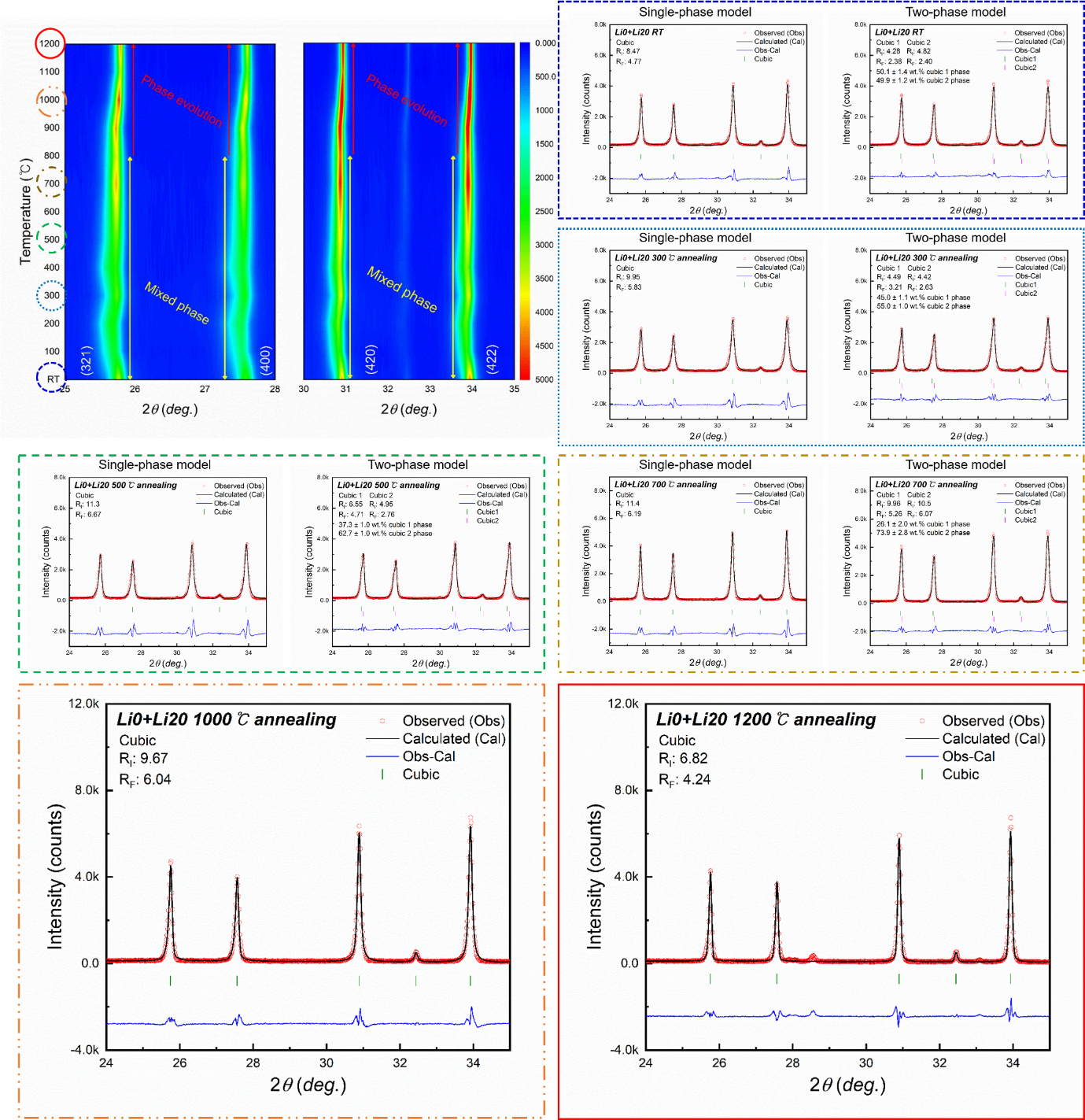
Figure S14**. *Ex situ* XRD contour plots and Rietveld refinement profiles of Li0– and Li20–LLZTO composites at room temperature (RT) and after annealing at 300°C, 500°C, 700°C, 1000°C, and 1200°C.

**
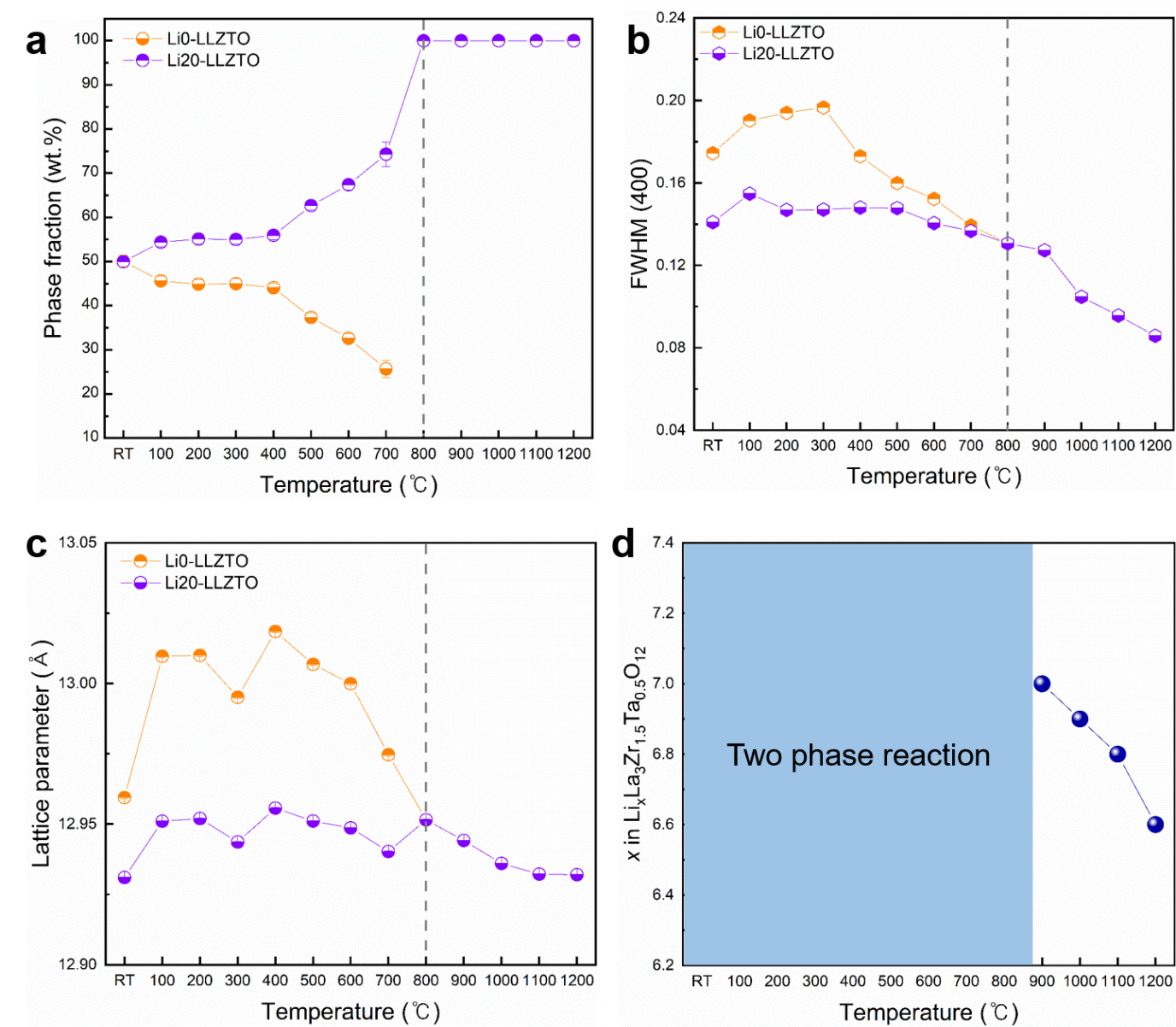
**

**Figure S15**. Structural parameter changes of Li0– and Li20–LLZTO composites during *ex* *situ* annealing. (a) Phase fractions, (b) full width at half maximum (FWHM) of the cubic (400) reflection, (c) lattice parameters derived from XRD Rietveld refinement, and (d) lithium content derived from ND Rietveld refinement.

**Supporting Text for Figure S15**

To validate the feasibility of Li and O incorporation during sintering, prior to applying the Li*y*/Li*x*–LLZTO approach, we investigated the thermal reactions of Li0–LLZTO with Li*y*–LLZTO (*y* = 0, 5, 10, 15, and 20) powders. As indicated by the TGA results in Figure S13, these mixtures exhibited similar decomposition trends for Li*x*–LLZTO, depending on the Li content. Based on these findings, a 1:1 *wt.*% mixture of Li0– and Li20–LLZTO was subjected to *ex situ* annealing to gain further insight into the phase evolution under Li/O-rich conditions. Figure S14 presents contour plots derived from the *ex situ* XRD patterns of the annealed samples. The mixed phase was sustained up to 700°C, after which the peaks began to merge, indicating the formation of a single phase above this temperature.

Rietveld refinement was performed using both single-phase and two-phase models, with the best fit determined from reliability factors and the evolution of lattice parameters and the full width at half maximum (FWHM). The two‑phase model provided better fits at intermediate temperatures (RT, 300°C, 500°C, and 700°C), consistent with phase separation in the Li0– and Li20–LLZTO mixture composite. The phase fraction, initially balanced at a 50:50 *wt.*% ratio at room temperature, shifted to 26:74 *wt.*% at 700°C (Figure S15a). At temperatures above 800°C, phase consolidation became evident, with peak merging and further changes in the FWHM and lattice parameters, for which the single‑phase model yielded a satisfactory fit (Figure S15b–c).

The Li content in the cubic structure was further evaluated using Rietveld refinement of the ND patterns of the mixed powders annealed at temperatures ranging from 900°C to 1200°C. A significantly larger amount of Li was incorporated into the cubic structure at 900°C; however, the Li content decreased to 6.6 pfu after annealing at 1200°C, attributable to Li sublimation at elevated temperatures in the absence of bedding powder. Notably, this Li content exceeds that of Li0– and Li20–LLZTO annealed at 900°C (Figure S8), indicating that Li‑ion diffusion occurred between the Li0– and Li20–LLZTO phases during annealing. These findings confirm that Li redistribution between compositionally distinct garnet phases can be harnessed to enhance the Li content within the structure, thereby establishing the Li*y*/Li*x*–LLZTO design as a compositionally driven route for vacancy suppression during sintering. The quantitative analysis was also conducted on the mixed powder of Li0– and Li20–LLZTO under 900°C; however, this temperature regime corresponds to a two-phase coexistence region, in which residual Li decomposes and vigorous Li-ion migration occurs between the two phases. As a result, the Li content tends to increase indefinitely, rendering quantitative evaluation practically unbounded.

**
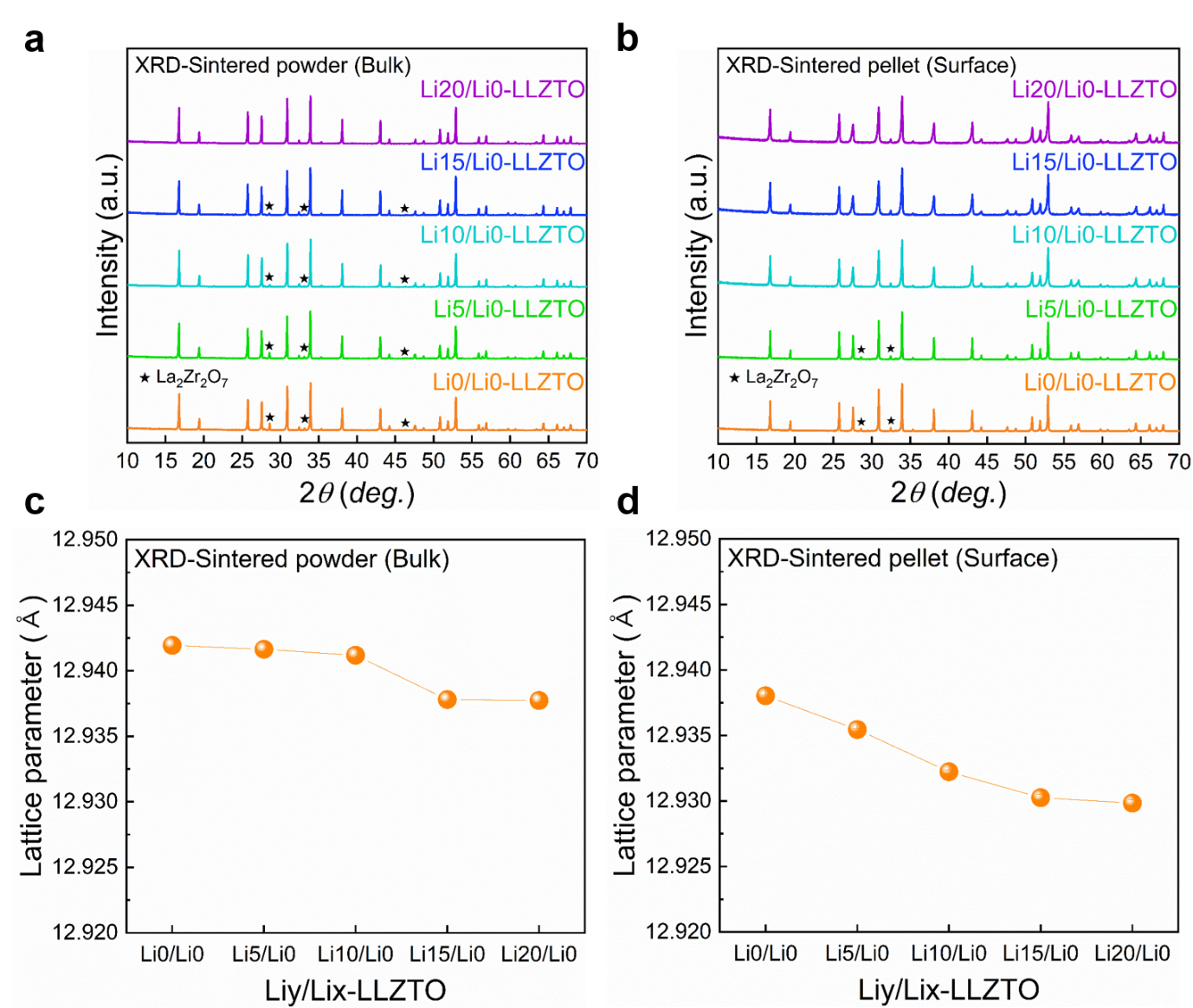
**

**Figure S16**. (a) Powder XRD and (b) pellet XRD patterns of sintered Li*y*/Li0–LLZTO samples (*y* = 0, 5, 10, 15, and 20). Lattice parameters derived from (c) powder XRD and (d) pellet XRD patterns of the same samples.

**
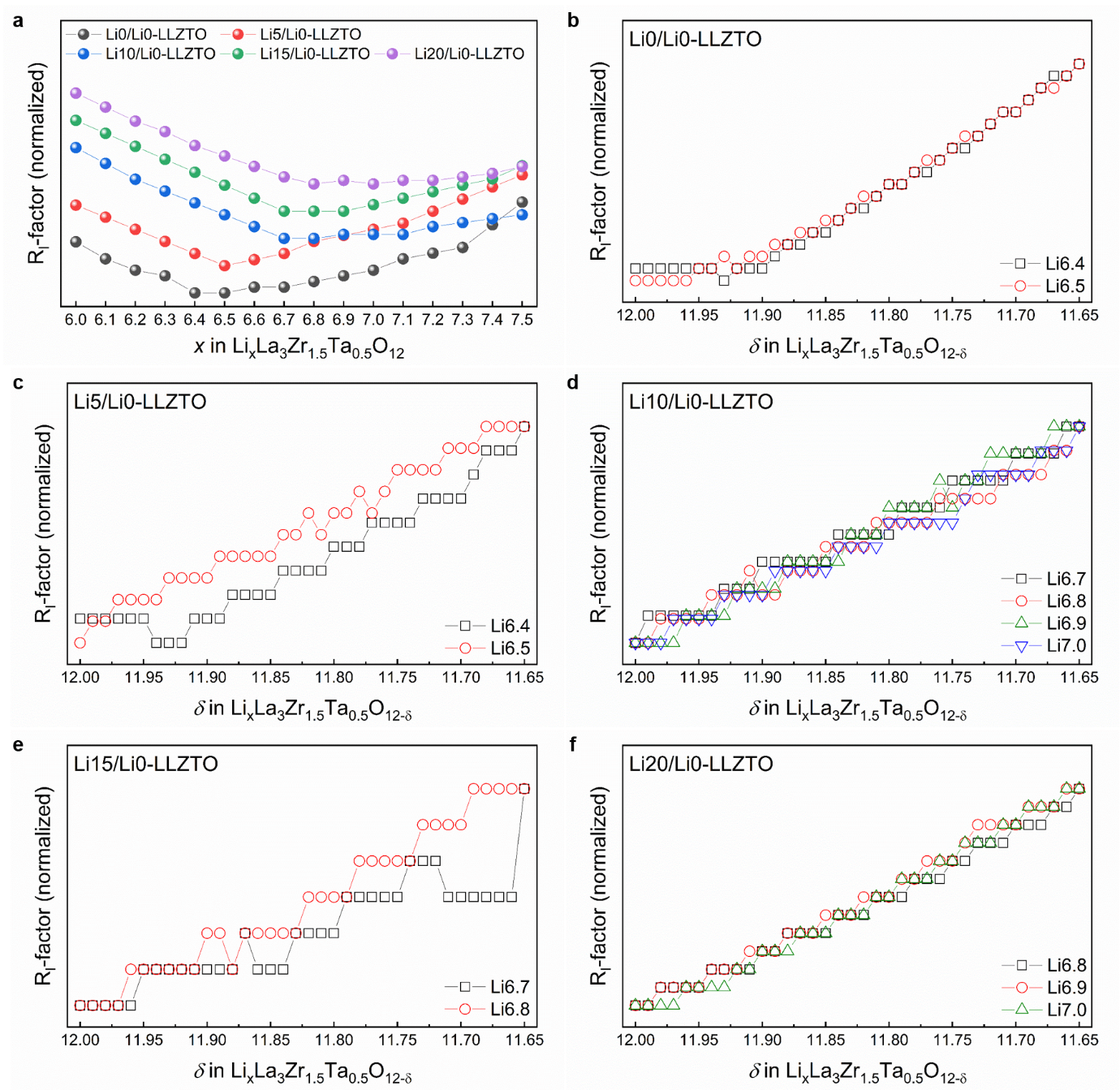
Figure S17**. (a) Quantitative analysis of Li content in the sintered cubic phase of Li*y*/Li0–LLZTO (*y* = 0, 5, 10, 15, and 20). Quantitative analysis of oxygen content in the sintered cubic phase of Li*y*/Li0–LLZTO (*y* = (b) 0, (c) 5, (d) 10, (e) 15, and (f) 20).

**Supporting Text for Figure S17**

The Li and O insertion observed under Li/O-rich sintering conditions can be explained by differences in the effective Li/O chemical potential environment established during high-temperature processing. Under the applied sintering conditions, LLZTO coexists with Li-containing secondary phases such as LiOH and Li_2_CO_3_, whose relative amounts systematically vary with the excess Li supplied during synthesis. As a result, the effective Li/O chemical potential buffered by these secondary phases is not fixed but evolves depending on the Li and O environment during sintering. In particular, a Li0-LLZTO pellet experiences fundamentally different chemical potential environments when sintered with Li20-LLZTO bedding compared to Li0-LLZTO bedding, leading to distinct thermodynamic driving forces for Li and O incorporation.

Under compositionally symmetric bedding conditions, the effective Li/O chemical potential gradient between the pellet and the surrounding powder remains limited, resulting in an insufficient thermodynamic driving force for diffusion and defect equilibration. Consequently, coupled Li and O vacancies persist after sintering (Figure 1f). In contrast, Li/O-rich bedding conditions establish a pronounced chemical potential gradient, which thermodynamically enables Li and O insertion into the garnet lattice and promotes vacancy equilibration during sintering (Figure 2c). This behavior is directly evidenced by ND analysis, which reveals a concurrent increase in Li occupancy and a reduction in oxygen vacancy concentration, and is further supported by the evolution of structural parameters during *ex situ* annealing (Figure S14) and quantitative Li analysis derived from ND (Figure S15d). Overall, the observed Li/O insertion can be understood as a composition redistribution process driven by differences in effective chemical potential, providing a thermodynamic basis for the distinct defect states obtained under different sintering conditions.

**
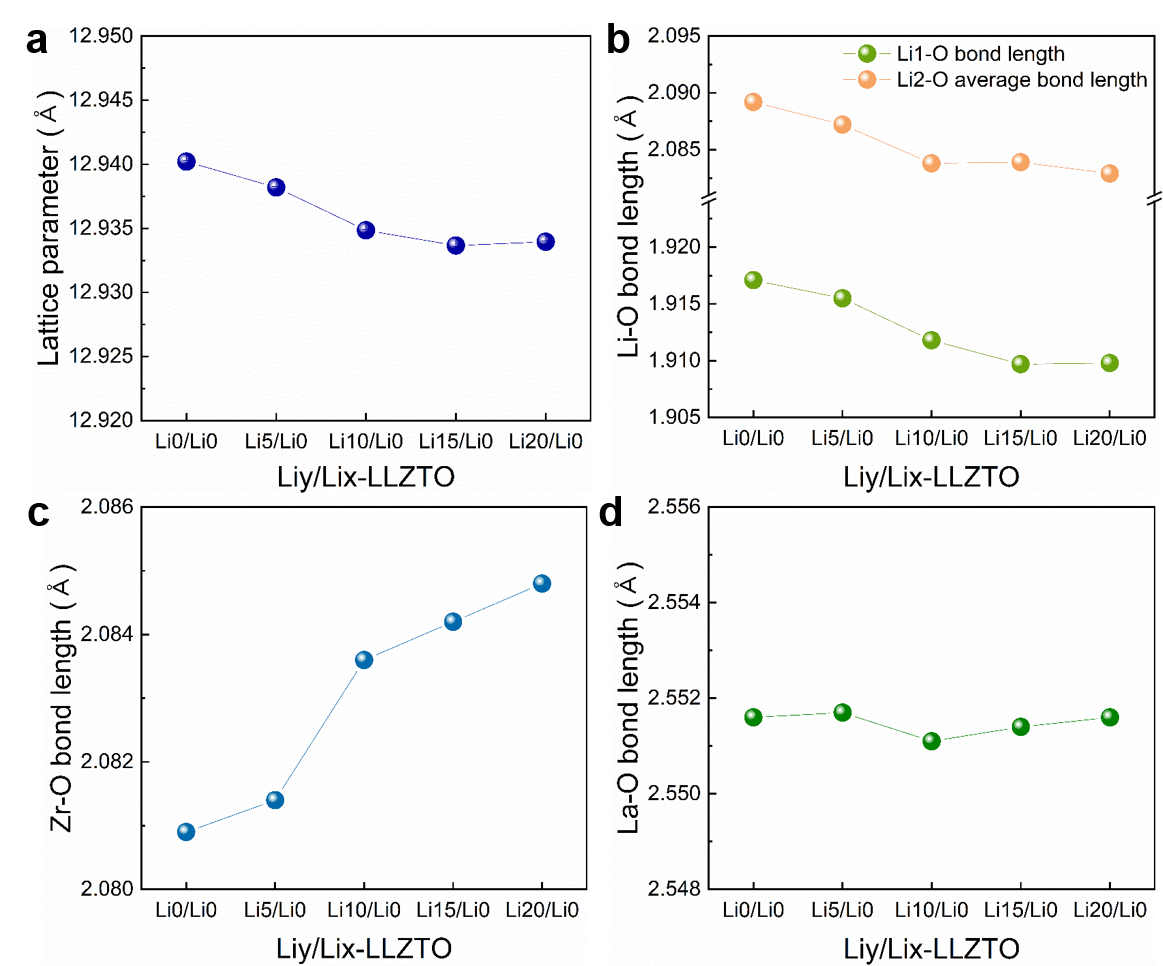
**

**Figure S18**. Structural parameters derived from ND patterns of Li*y*/Li0–LLZTO (*y* = 0, 5, 10, 15, and 20). (a) Lattice parameter variations, (b) Li–O bond length variations, (c) Zr–O bond length variation, and (d) La–O bond length variation.

**Supporting Text for Figure S18**

To elucidate the structural evolution associated with Li enrichment and O-vacancy suppression under a Li/O-rich atmosphere, variations in key structural parameters were analyzed as a function of quantified Li and O contents. As shown in Figure S18b–c, the Li–O bond length decreased with increasing Li content, whereas the Zr–O bond length gradually increased. In contrast, the La–O bond length remained nearly unchanged across all compositions (Figure S18d). Given that the garnet framework is formed by corner-sharing oxygen, changes in Li–O distances are expected to modulate local strain.

To assess strain evolution, the bond angle variance (BAV) of Li and Zr coordination polyhedra was calculated (Figure 2d–e). Li enrichment caused minimal distortion of the Li1–O tetrahedra but significant distortion of the Li2–O octahedra. This distortion in Li2–O units relieved local strain in adjacent ZrO_6_ octahedra, reducing Zr–O bond angle disorder and enhancing Zr–O ordering, accompanied by a gradual elongation of the Zr–O bond length.

These observations parallel structural trends reported for garnets undergoing a tetragonal-to-cubic transition. In tetragonal garnets, ordered Li mitigates Li–Li repulsion, lowering strain in the Li–O network but increasing ZrO_6_ distortion.^[1-2]^ In vacancy-stabilized cubic garnets, Li–Li repulsion is enhanced, raising strain in the Li–O network while relieving ZrO_6_ distortion. In our samples, preferential occupation of the Li2 (96h) site (Table S7) intensifies Li2–O distortions yet concurrently relaxes the ZrO_6_ framework. This dual effect increases symmetry and stabilizes the cubic phase well beyond the nominal 6.5 pfu.

**
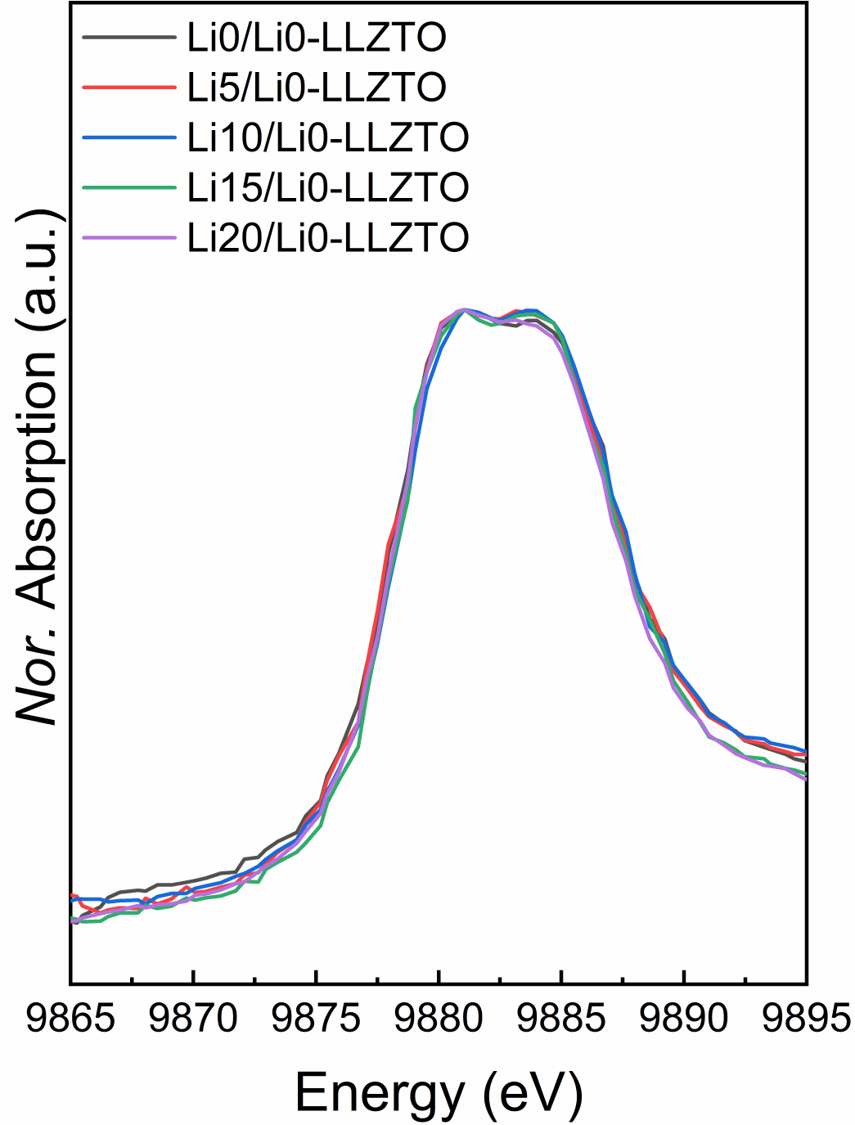
**

**Figure S19**. Ta L_3_-edge XAS of Li*y*/Li0–LLZTO (*y* = 0, 5, 10, 15, and 20).

**
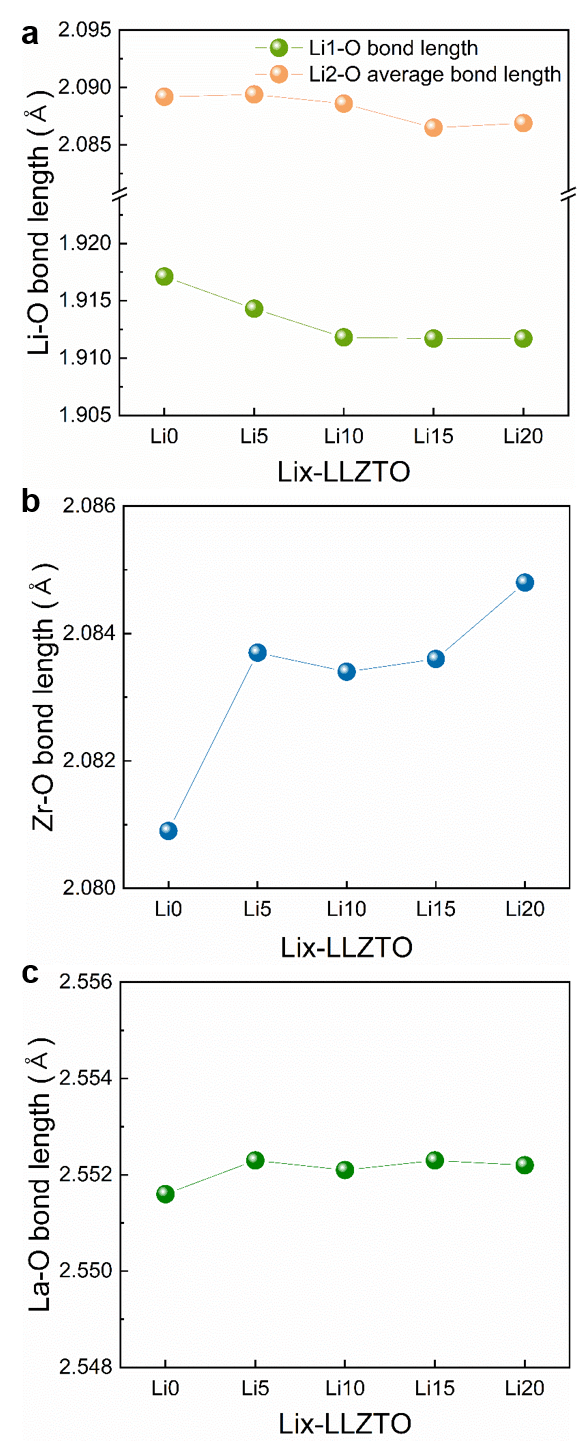
**

**Figure S20**. Structural parameters derived from ND patterns of Li*x*–LLZTO (*x* = 0, 5, 10, 15, and 20). (a) Variation in Li–O bond lengths, (b) variation in Zr–O bond length, and (c) variation in La–O bond length.

**
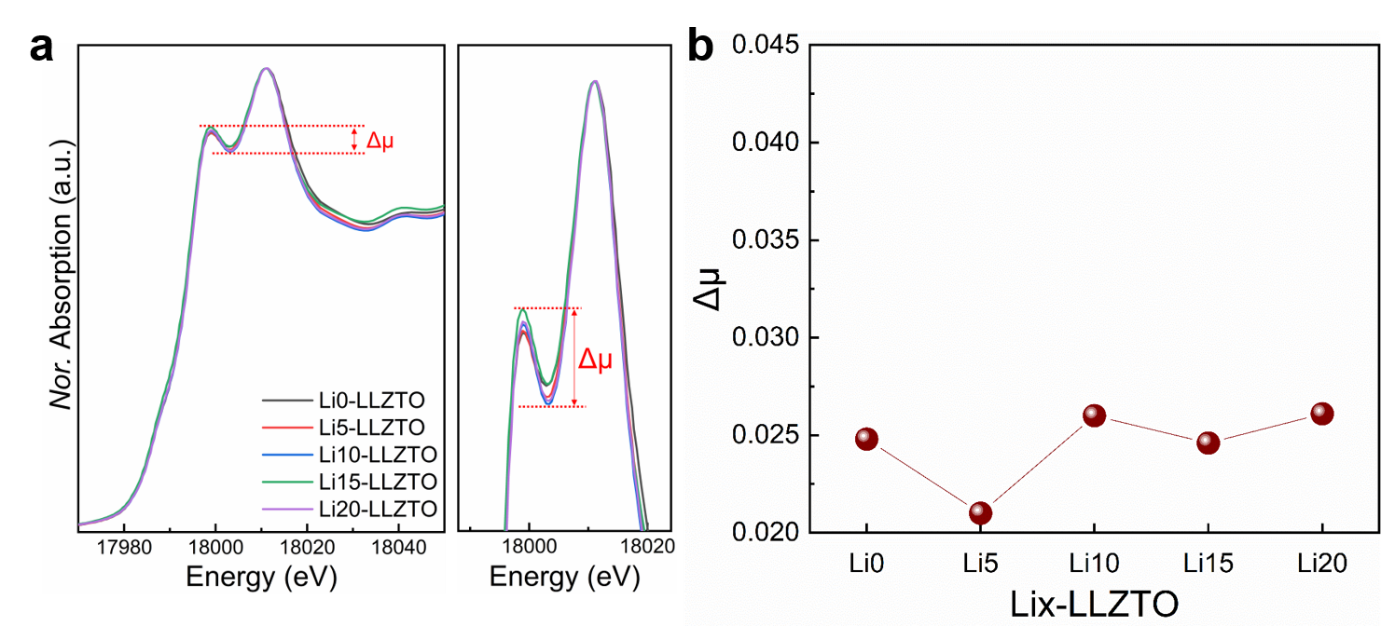
Figure S21**. (a) Zr K-edge XAS of Li*x*–LLZTO. (b) Variations in absorption coefficients (Δμ) of ZrO_6_ octahedra in Li*x*–LLZTO (*x* = 0, 5, 10, 15, and 20).

**
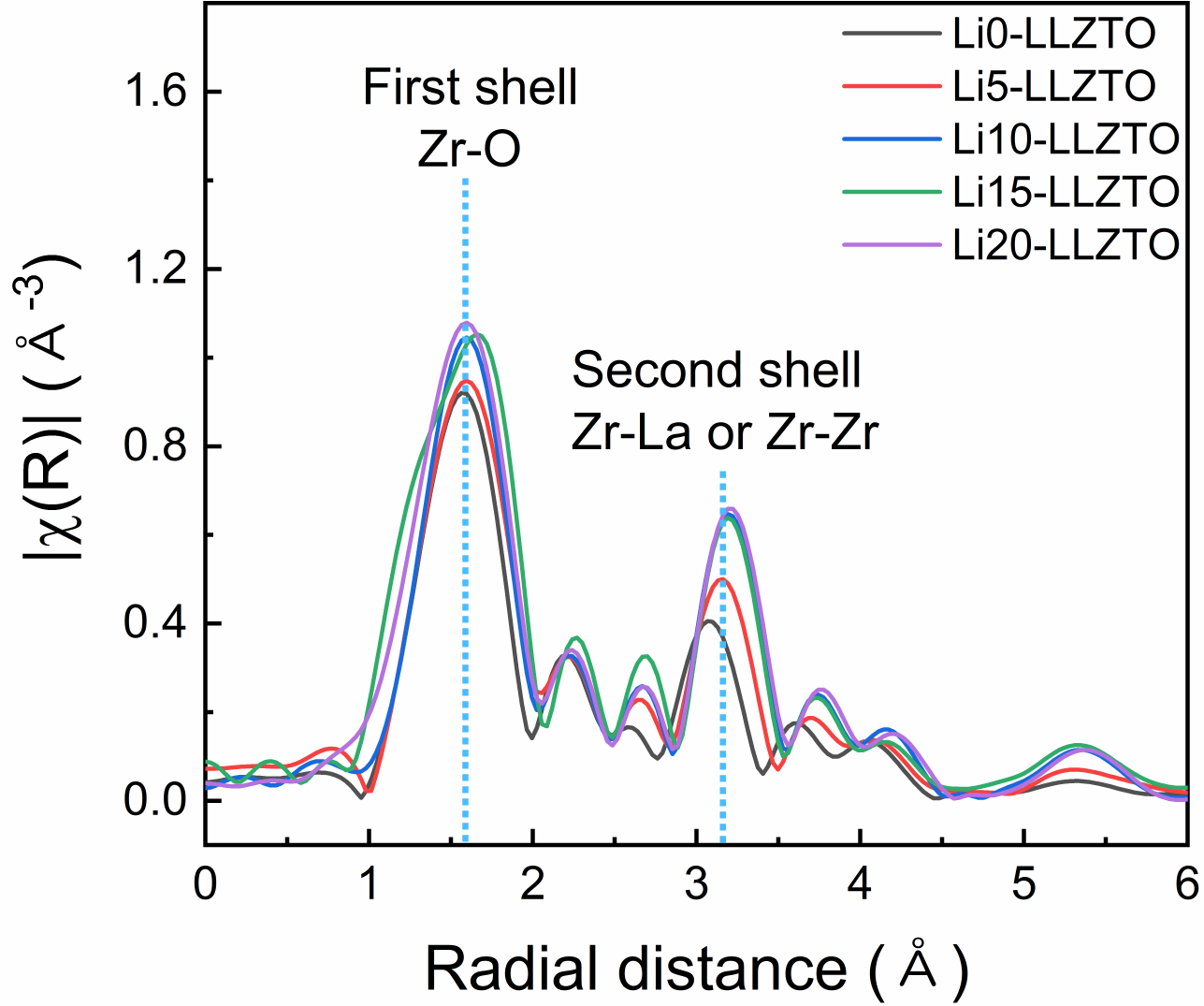
**

**Figure S22**. EXAFS spectra at the Zr K-edge of Li*x*–LLZTO (*x* = 0, 5, 10, 15, and 20).

**
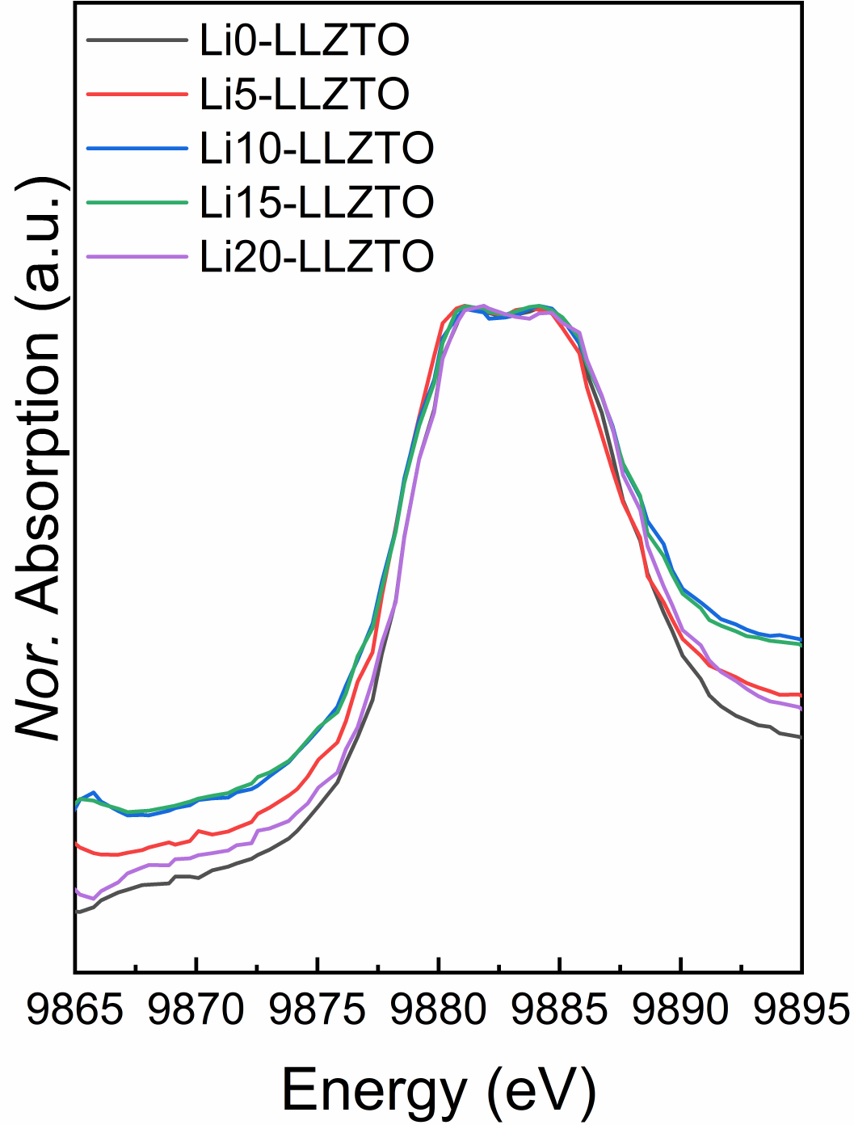
**

**Figure S23**. Ta L_3_-edge XAS of Li*x*–LLZTO (*x* = 0, 5, 10, 15, and 20).

**
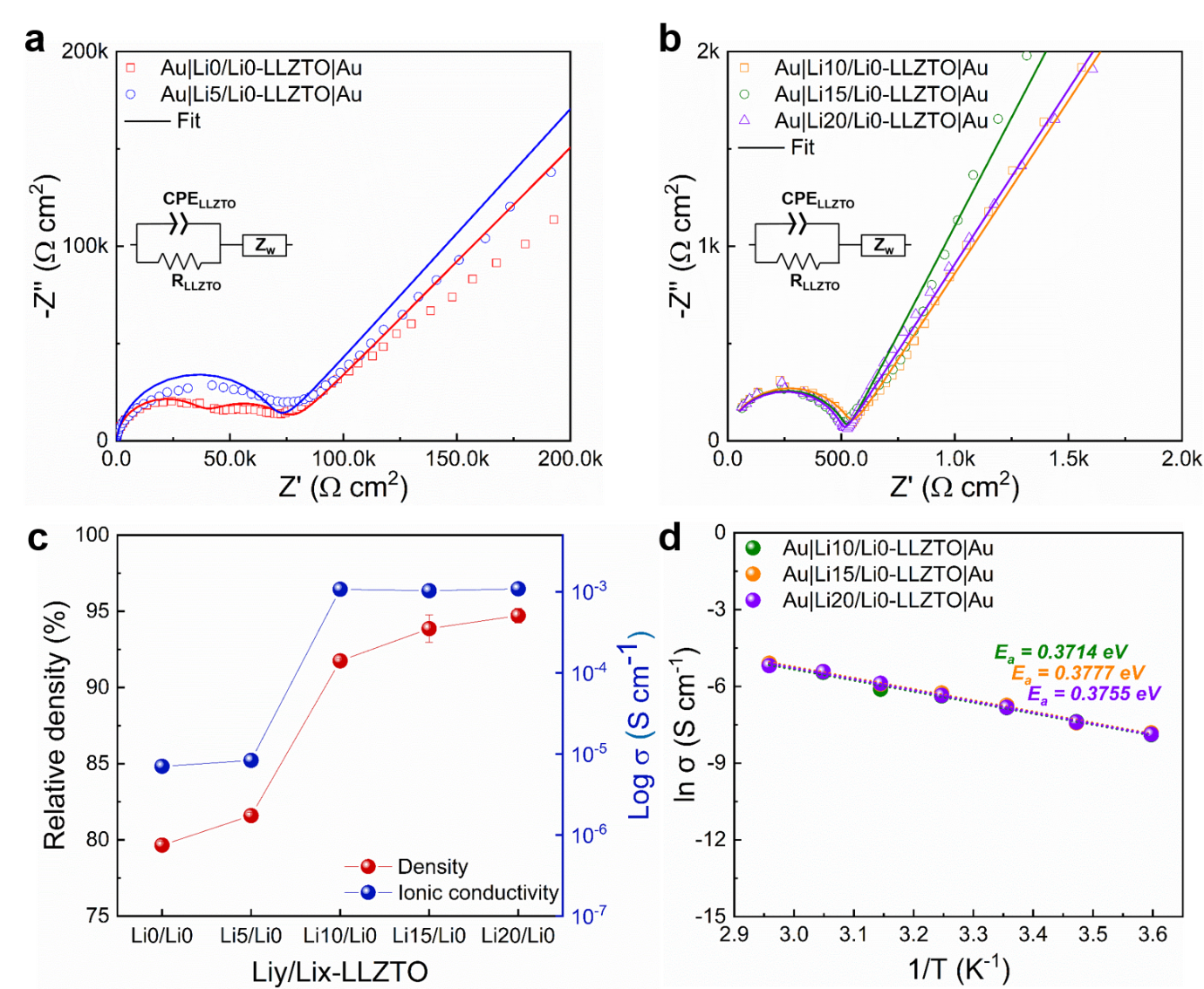
**

**Figure S24**. Nyquist plots of Au|Li*y*/Li0–LLZTO|Au symmetric cells; (a) *y* = 0 and 5, (b) *y* = 10, 15, and 20. The insets in (a) and (b) display the equivalent circuit model employed for fitting the impedance spectra, together with the extracted resistance components. (c) Relative density and ionic conductivity of Li*y*/Li0–LLZTO (*y* = 0, 5, 10, 15, and 20). (d) Arrhenius plots for Li*y*/Li0–LLZTO (*y* = 10, 15, and 20).

**
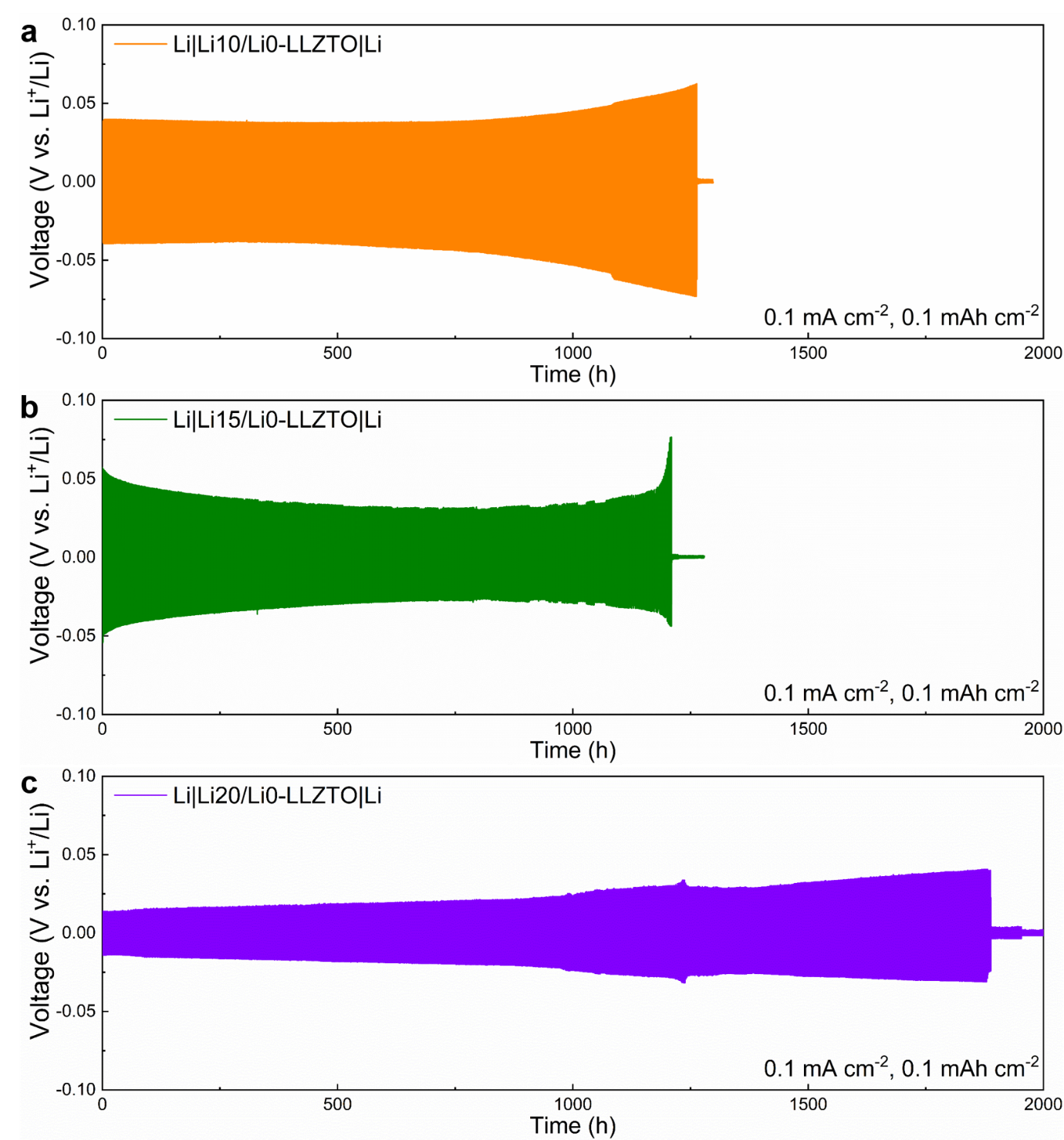
**

**Figure S25**. Voltage profiles for Li*y*/Li0–LLZTO (*y* = (a) 10, (b) 15, and (c) 20) Li symmetric cells measured at a current density 0.1 mA cm^−2^ at 30°C.

**Supporting Text for Figure S25**

In this study, lithium symmetric cell cycling was conducted at a limited areal capacity of 0.1 mAh cm^-2^ to enable a reproducible evaluation of intrinsic interfacial stability under a lithium metal electrode with a thickness of 20 μm. Under such conditions, the influence of lithium depletion and mechanical effects associated with large plating volumes is minimized, allowing a direct and systematic comparison of interfacial reaction behavior and stability among different garnet electrolytes.

**
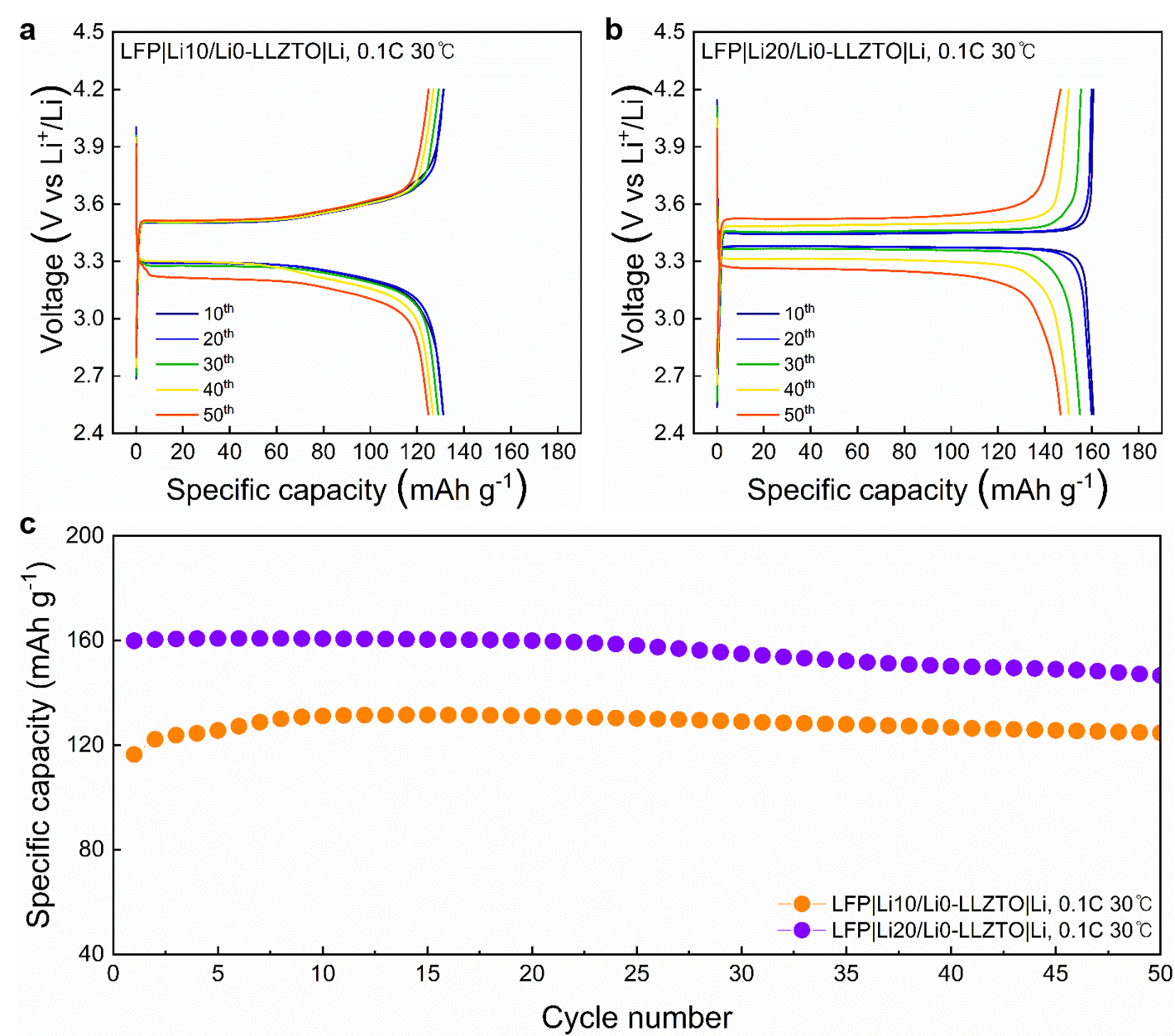
**

**Figure S26**. Voltage profiles of LFP|Li*y*/Li0–LLZTO|Li hybrid full-cells at 0.1C and 30°C (*y* = (a) 10, and (b) 20). (c) Discharge capacity of LFP|Li*y*/Li0–LLZTO|Li hybrid full-cells cycled at 30°C (*y* = 10 and 20).


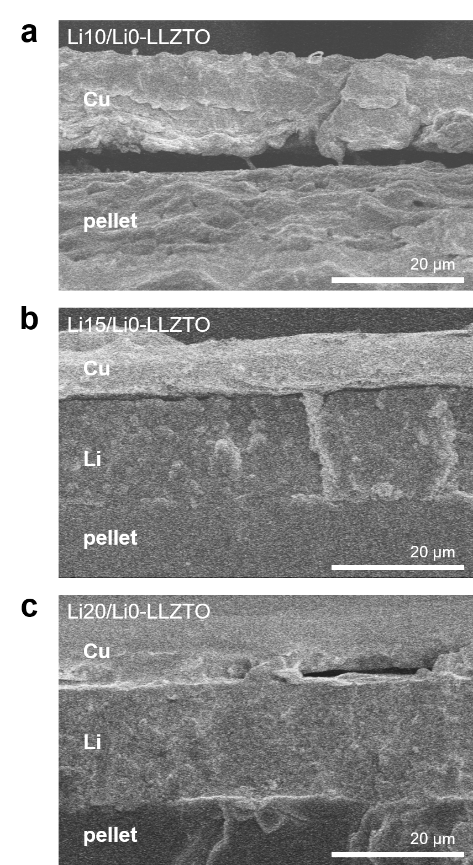


**Figure S27.** Cross-sectional scanning electron microscope (SEM) images of lithium symmetric cells assembled with Li*y*/Li0-LLZTO pellet after cycling (*y* = (a) 10, (b) 15, and (c) 20).


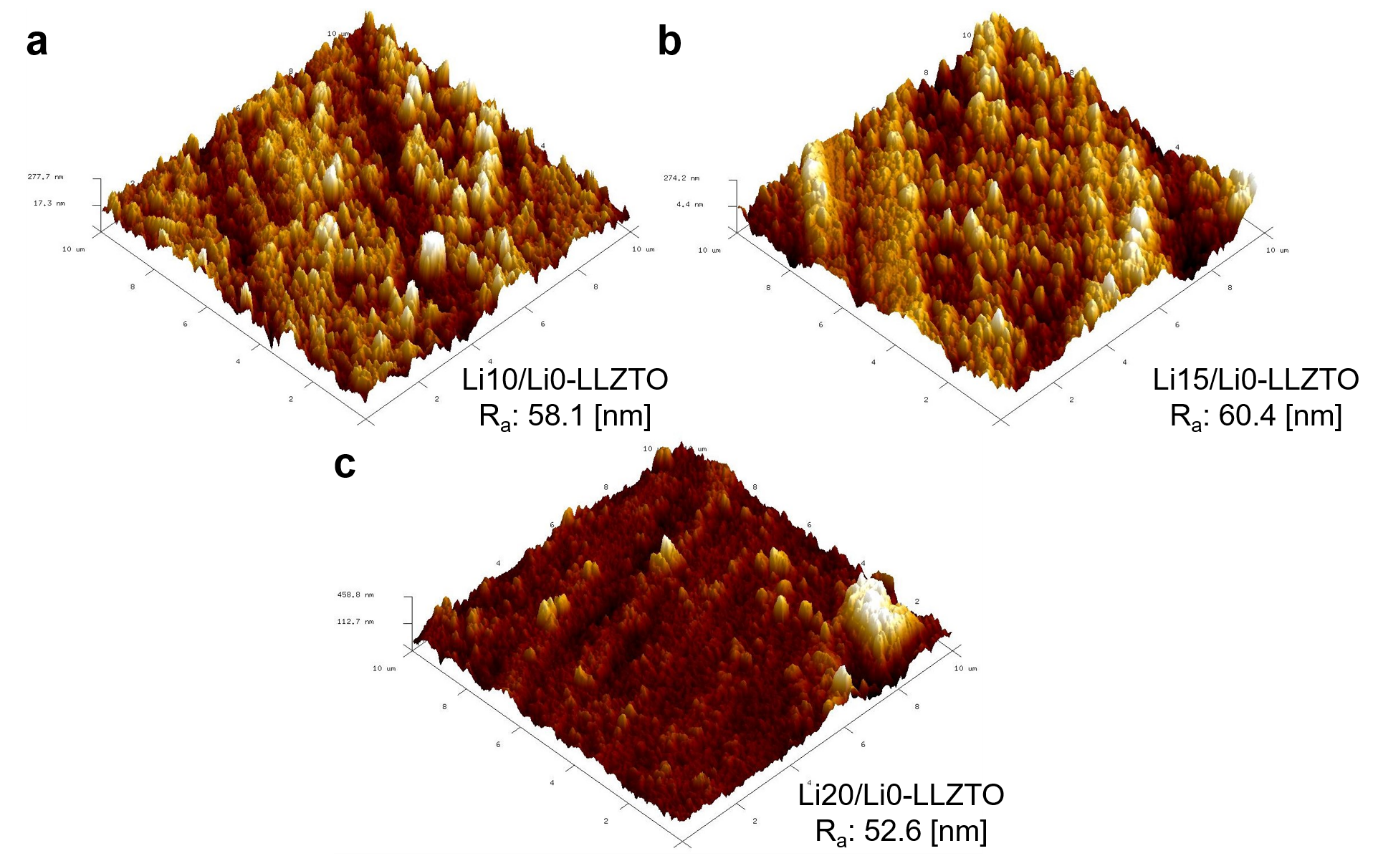


**Figure S28**. Three–dimensional rendered atomic force microscope surface topography of Li*y*/Li0–LLZTO pellets (*y* = (a) 10, (b) 15, and (c) 20).





**Figure S29.** Li^+^/H^+^ exchange energies calculated for the Li_n_La_3_Zr_1.5_Ta_0.5_O_8.75+0.5n_ series (n=5.75, 6, 6.25, and 6.5), corresponding to the energy difference for the Z=8 supercell. The exchange energy is defined based on the replacement of 1/8 Li with 1/8 H per formula unit relative to the pristine structure.


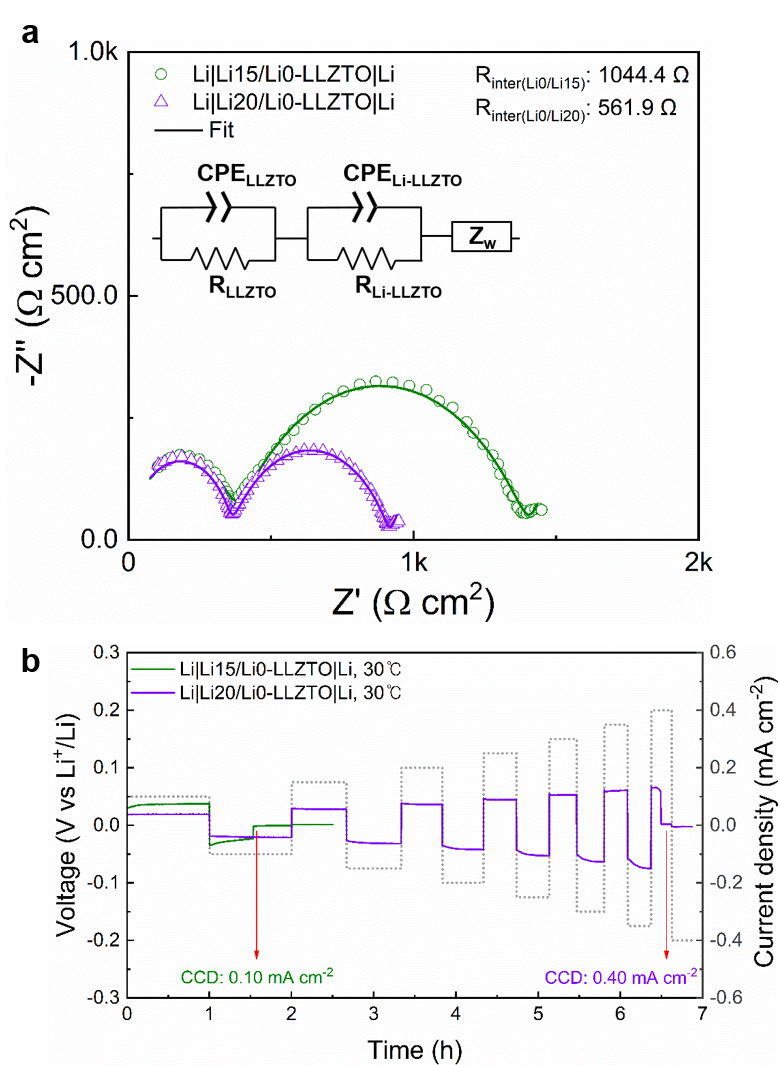


**Figure S30**. Electrochemical measurements for analyzing proton exchange behavior in garnet. (a) Nyquist plots of lithium symmetric cells using Li*y*/Li0–LLZTO pellets (*y* = 15 and 20) after exposing the pellet to ambient air for 1 day. (b) Critical current density (CCD) tests of corresponding lithium symmetric cells. The inset in (a) displays the equivalent circuit model employed for fitting the impedance spectra, together with the extracted resistance components.

**
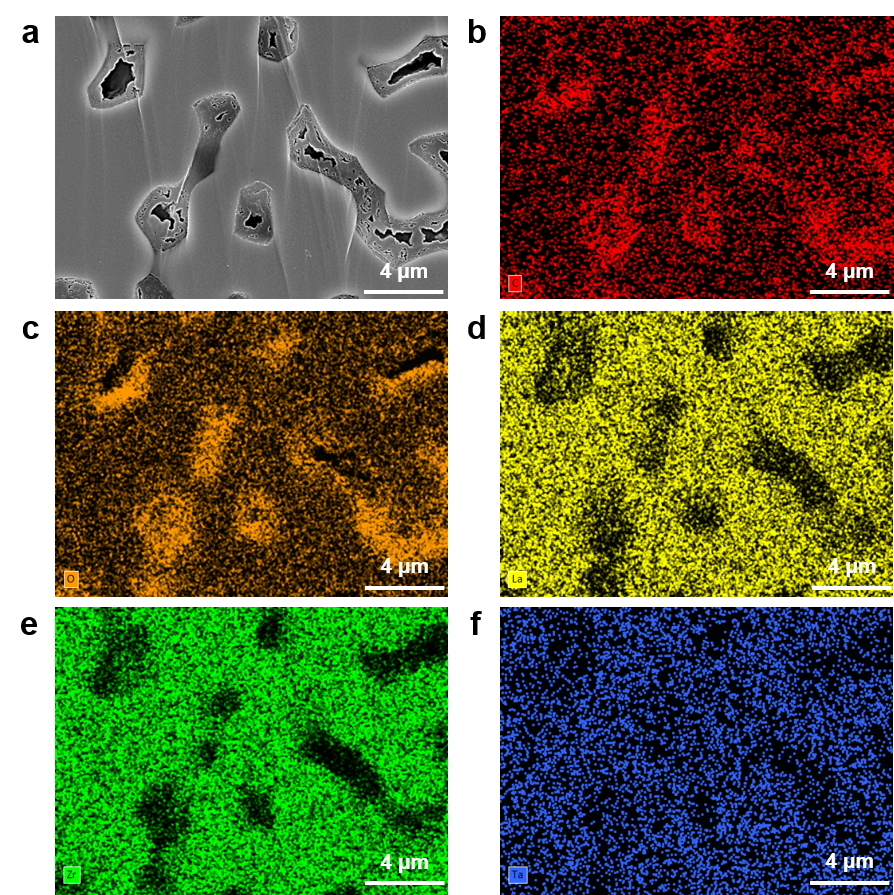
**

**Figure S31**. SEM and energy-dispersive X-ray spectroscopy (EDS) images of cross-sectional tailored pellet of Li10/Li0–LLZTO. (a) SEM image and EDS mapping images of (b) carbon, (c) oxygen, (d) lanthanum, (e) zirconium, and (f) tantalum.

**
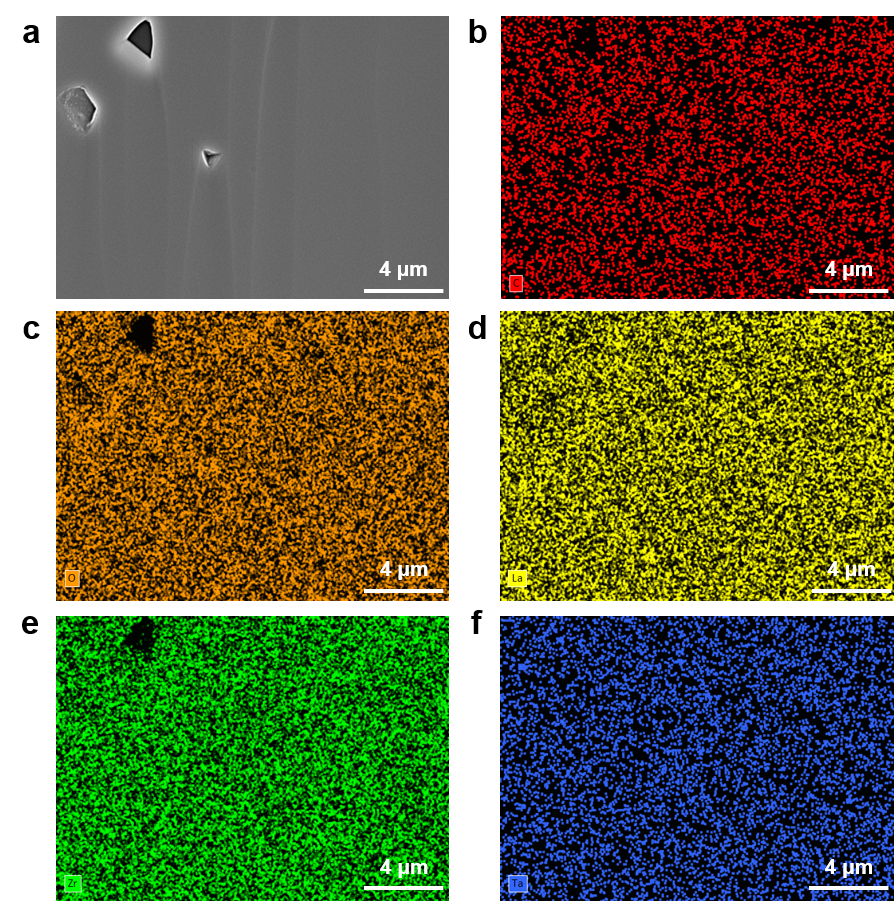
**

**Figure S32**. SEM–EDS images of cross-sectional tailored pellet of Li15/Li0–LLZTO. (a) SEM image and EDS mapping images of (b) carbon, (c) oxygen, (d) lanthanum, (e) zirconium, and (f) tantalum.

**
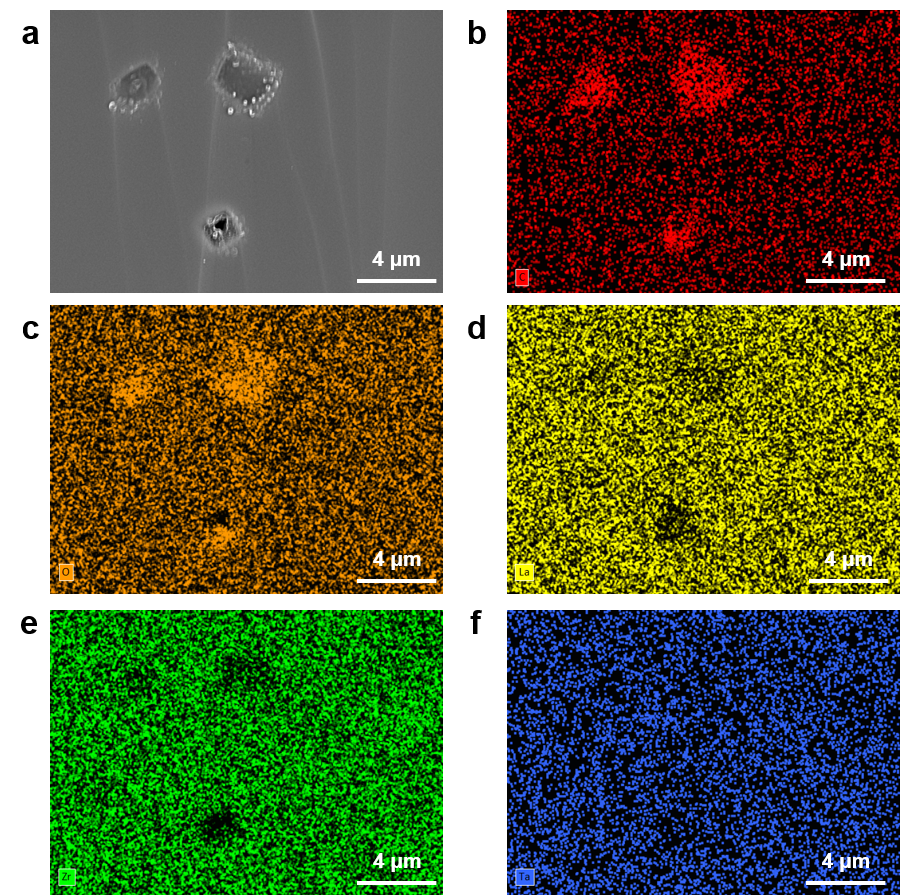
**

**Figure S33**. **a–f**, SEM–EDS images of cross-sectional tailored pellet of Li20/Li0–LLZTO. (a) SEM image and EDS mapping images of (b) carbon, (c) oxygen, (d) lanthanum, (e) zirconium, and (f) tantalum.

**
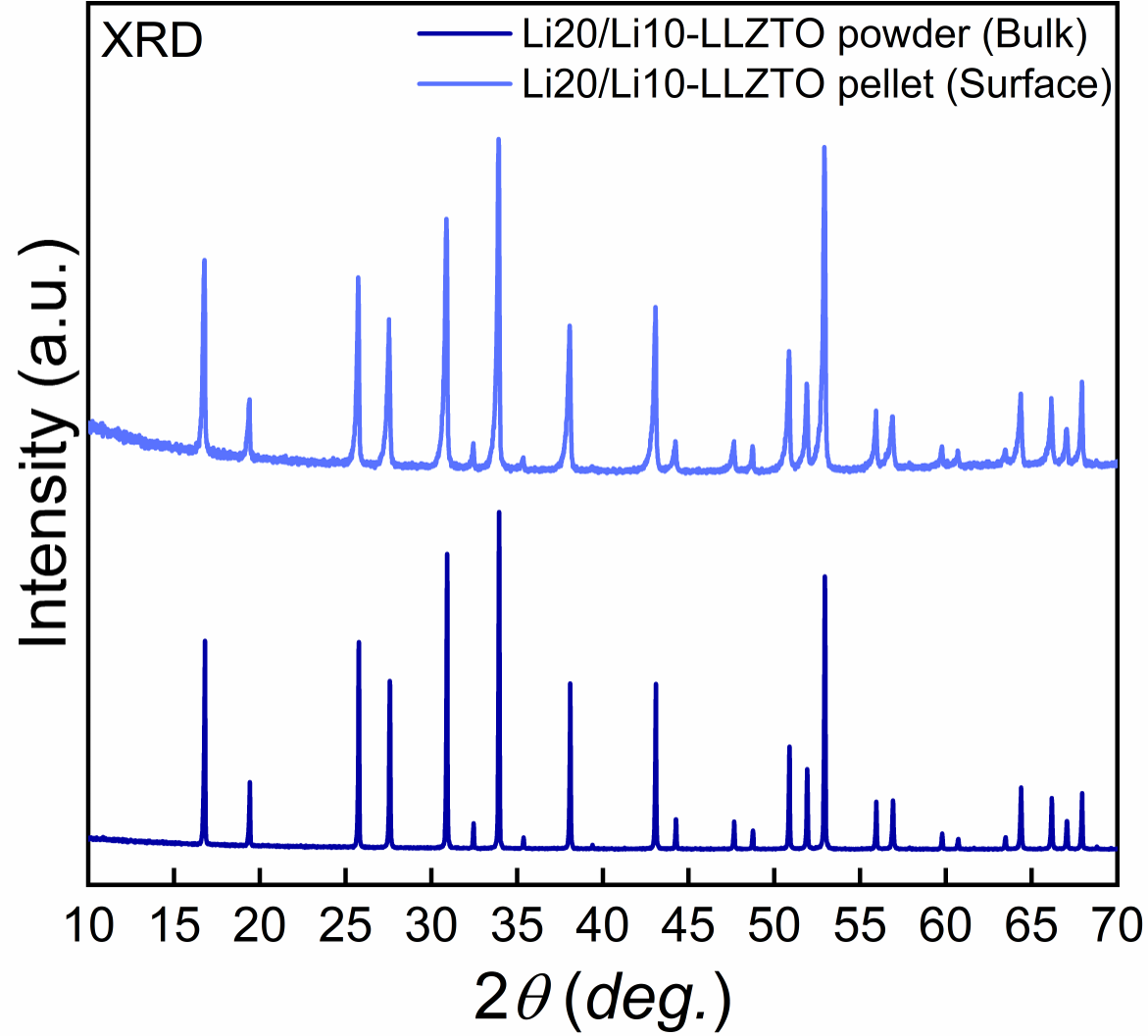
**

**Figure S34**. XRD patterns of Li20/Li10–LLZTO powder and pellet.

**
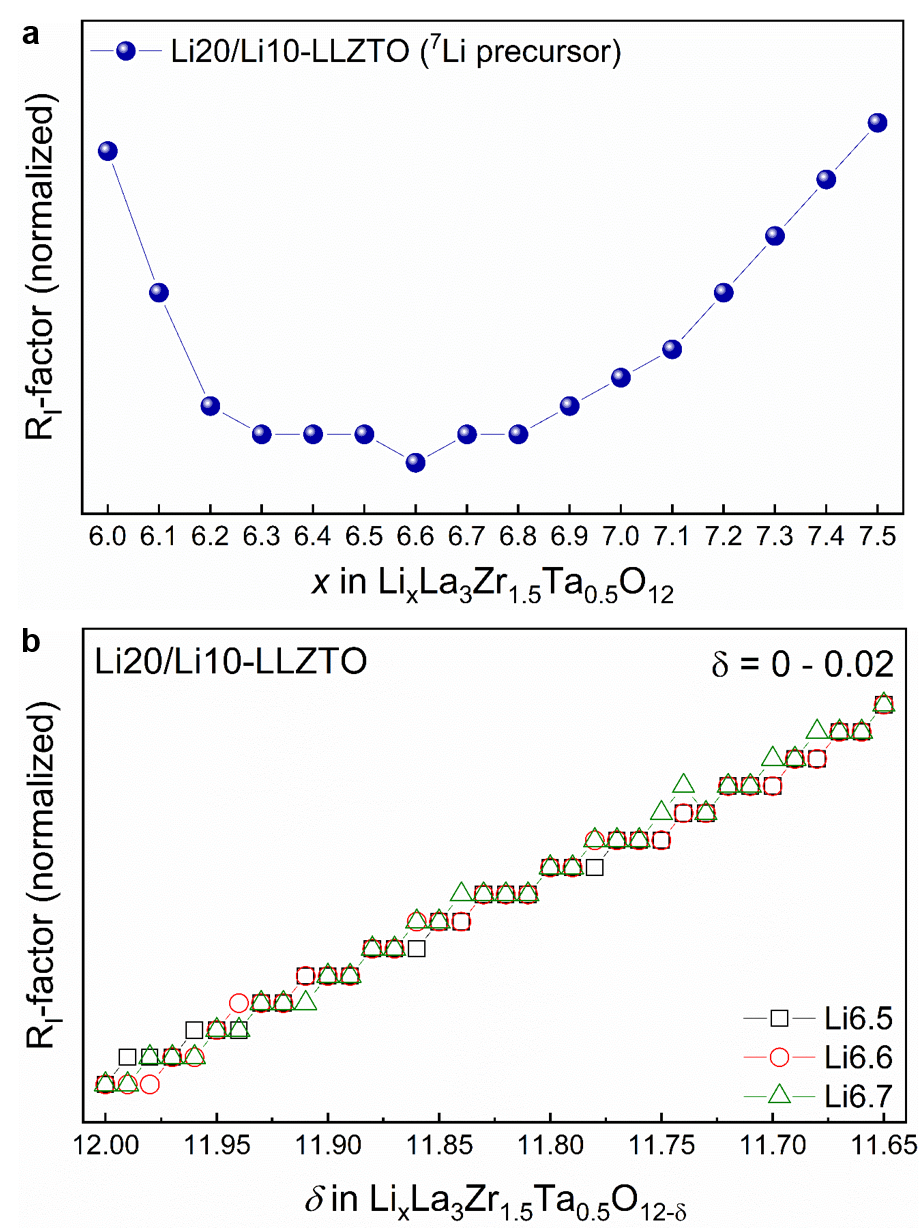
**

**Figure S35**. Quantitative analysis of lithium and oxygen contents in Li20/Li10–LLZTO synthesized using a ^7^Li precursor: (a) lithium content and (b) oxygen content.

**Supporting Text for Figure S35**

Natural lithium contains ~7.5% ^6^Li and ~92.5% ^7^Li. The neutron absorption cross section of ^6^Li is approximately 940 barns, which is four orders of magnitude higher than that of ^7^Li (~0.045 barns). The large absorption of ^6^Li can significantly attenuate the diffraction intensity and may limit the overall quality of the acquired data. By using ^7^Li-enriched precursor, the neutron attenuation was greatly reduced, enhancing the signal-to-noise ratio and improving the accuracy of Li-site occupancy refinement.

**
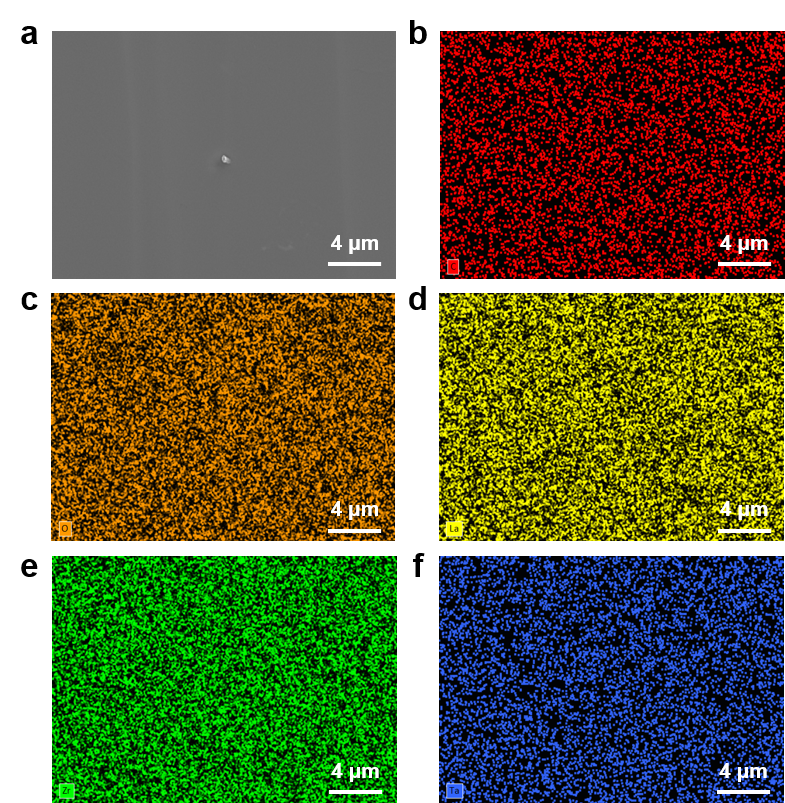
**

**Figure S36**. SEM–EDS images of cross-sectional tailored pellet of Li20/Li10–LLZTO. (a) SEM image and EDS mapping images of (b) carbon, (c) oxygen, (d) lanthanum, (e) zirconium, and (f) tantalum.

**
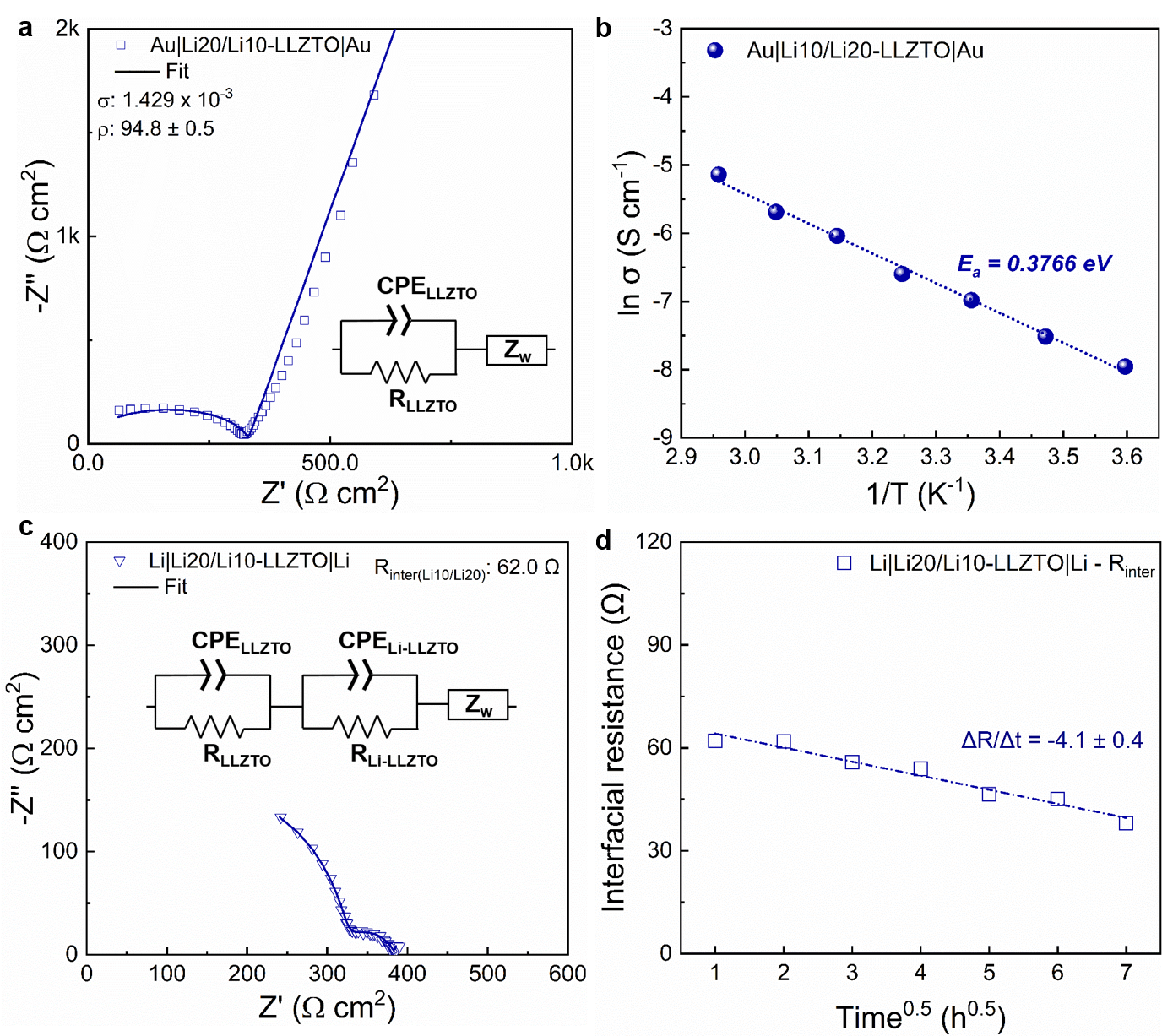
**

**Figure S37**. Electrochemical measurements of Li20/Li10–LLZTO. Nyquist plot of (a) Au symmetric cell. (b) Arrhenius plot. (c) Nyquist plot of lithium symmetric cell. (d) Interfacial resistance derived from time-dependent electrochemical impedance spectroscopy of lithium symmetric cell. The insets in (a) and (c) display the equivalent circuit model employed for fitting the impedance spectra, together with the extracted resistance components.

**
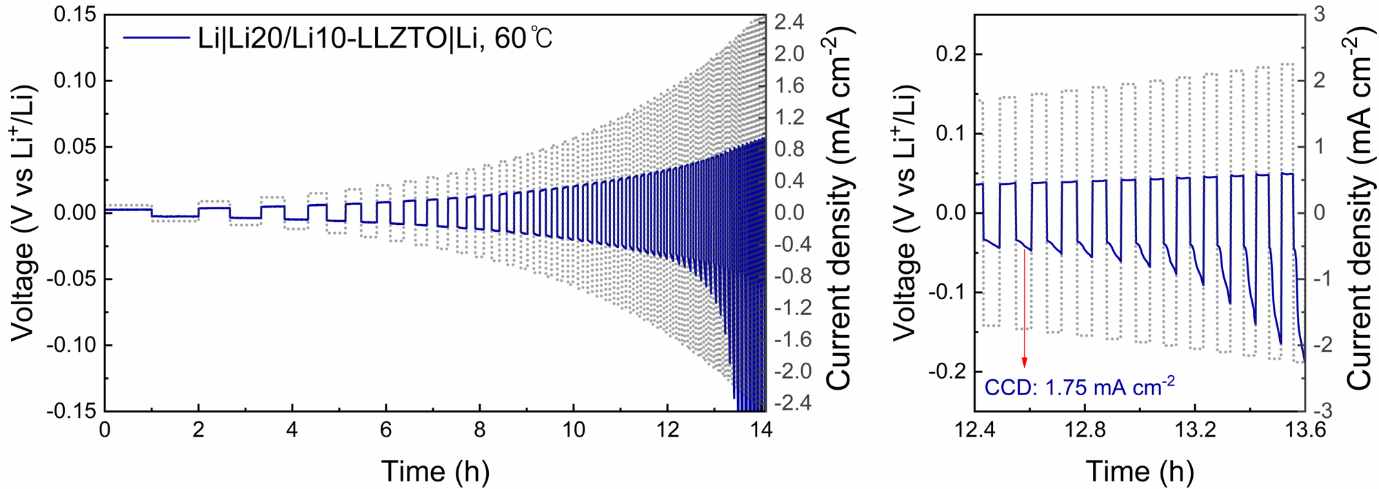
**

**Figure S38**. CCD measurement of lithium symmetric cell using Li20/Li10–LLZTO pellet measured at 60°C.





**Figure S39**. Correlation between ionic conductivity and CCD of garnet-type solid electrolytes, comparing this study with previously reported values.^[3-48]^

**
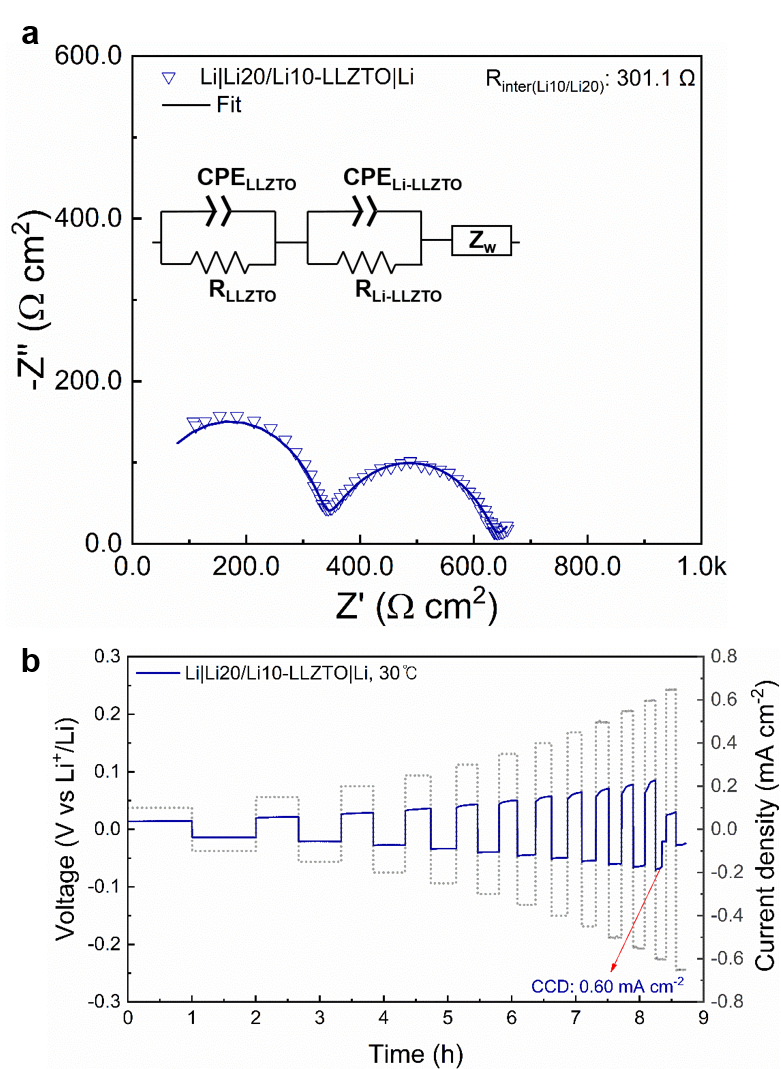
**

**Figure S40**. Electrochemical measurements for analyzing proton exchange behavior in garnet. (a) Nyquist plot of lithium symmetric cell using Li20/Li10–LLZTO pellet after exposing the pellet to ambient air for 1 day. (b) CCD test of corresponding lithium symmetric cell. The inset in (a) displays the equivalent circuit model employed for fitting the impedance spectra, together with the extracted resistance components.

**Table S1**. Amount of La_2_O_3_ impurity phase derived from Rietveld analysis of XRD patterns of Li*x*–LLZTO (*x* = 0, 5, 10, 15, and 20) calcined at 900°C.

| Calcined Li*x*–LLZTO | La_2_O_3_ [*wt.*%] |
| --- | --- |
| 0 | 0.88 |
| 5 | – |
| 10 | – |
| 15 | – |
| 20 | – |

**Table S2**. Structural parameters derived from Rietveld refinement of ND patterns of calcined Li*x*–LLZTO (*x* = 0, 5, 10, 15, and 20).

| Calcined Li0–LLZTO | | | | | |
| --- | --- | --- | --- | --- | --- |
| Site | *x* | *y* | *z* | B_iso_ | *Occ.* |
| La | 0.125 | 0 | 0 | 0.86 (4) | 1 |
| Zr | 0 | 0 | 0 | 0.94 (5) | 0.75 |
| Ta | 0 | 0 | 0 | 0.94 (5) | 0.25 |
| Li1 | 0.375 | 0 | 0.25 | 1.80 (24) | 0.446 (12) |
| Li2 | 0.6881 (12) | 0.5798 (11) | 0.1003 (11) | 1.80 (24) | 0.405 (3) |
| O | 0.2814 (1) | 0.1018 (1) | 0.1964 (1) | 1.01 (3) | 0.9875 |

| Calcined Li5–LLZTO | | | | | |
| --- | --- | --- | --- | --- | --- |
| Site | *x* | *y* | *z* | B_iso_ | *Occ.* |
| La | 0.125 | 0 | 0 | 0.84 (3) | 1 |
| Zr | 0 | 0 | 0 | 0.85 (4) | 0.75 |
| Ta | 0 | 0 | 0 | 0.85 (4) | 0.25 |
| Li1 | 0.375 | 0 | 0.25 | 1.87 (20) | 0.430 (12) |
| Li2 | 0.6909 (10) | 0.5818 (9) | 0.1009 (9) | 1.97 (20) | 0.409 (3) |
| O | 0.2814 (1) | 0.1017 (1) | 0.1964 (1) | 0.98 (2) | 0.9875 |

| Calcined Li10–LLZTO | | | | | |
| --- | --- | --- | --- | --- | --- |
| Site | *x* | *y* | *z* | B_iso_ | *Occ.* |
| La | 0.125 | 0 | 0 | 0.67 (4) | 1 |
| Zr | 0 | 0 | 0 | 0.99 (6) | 0.75 |
| Ta | 0 | 0 | 0 | 0.99 (6) | 0.25 |
| Li1 | 0.375 | 0 | 0.25 | 1.38 (25) | 0.403 (16) |
| Li2 | 0.6927 (12) | 0.5821 (11) | 0.1014 (11) | 1.38 (25) | 0.416 (4) |
| O | 0.2818 (1) | 0.1016 (1) | 0.1964 (1) | 1.05 (4) | 0.9875 |

| Calcined Li15–LLZTO | | | | | |
| --- | --- | --- | --- | --- | --- |
| Site | *x* | *y* | *z* | B_iso_ | *Occ.* |
| La | 0.125 | 0 | 0 | 0.64 (5) | 1 |
| Zr | 0 | 0 | 0 | 1.05 (7) | 0.75 |
| Ta | 0 | 0 | 0 | 1.05 (7) | 0.25 |
| Li1 | 0.375 | 0 | 0.25 | 1.33 (29) | 0.417 (16) |
| Li2 | 0.6961 (13) | 0.5850 (13) | 0.1061 (14) | 1.33 (29) | 0.412 (4) |
| O | 0.2819 (1) | 0.1020 (2) | 0.1963 (2) | 1.06 (4) | 0.9875 |

| Calcined Li20–LLZTO | | | | | |
| --- | --- | --- | --- | --- | --- |
| Site | *x* | *y* | *z* | B_iso_ | *Occ.* |
| La | 0.125 | 0 | 0 | 0.80 (4) | 1 |
| Zr | 0 | 0 | 0 | 1.05 (5) | 0.75 |
| Ta | 0 | 0 | 0 | 1.05 (5) | 0.25 |
| Li1 | 0.375 | 0 | 0.25 | 2.28 (25) | 0.504 (16) |
| Li2 | 0.6929 (13) | 0.5885 (12) | 0.1039 (13) | 2.28 (25) | 0.391 (4) |
| O | 0.2817 (1) | 0.1014 (1) | 0.1960 (1) | 1.10 (3) | 0.9875 |

|  | Li0 | Li5 | Li10 | Li15 | Li20 |
| --- | --- | --- | --- | --- | --- |
| R_p_ (%) | 4.53 | 3.89 | 6.00 | 6.61 | 3.32 |
| R_wp_ (%) | 6.12 | 5.03 | 7.78 | 8.55 | 4.62 |
| R_F_ (%) | 2.51 | 2.49 | 2.99 | 3.21 | 3.22 |
| R_I_ (%) | 3.46 | 3.36 | 4.3 | 4.52 | 4.05 |
| a (Å) | 12.9289 (2) | 12.9301 (2) | 12.9265 (3) | 12.9246 (3) | 12.9402 (2) |
| Vol. (Å^3^) | 2161.16 (7) | 2161.75 (5) | 2159.94 (7) | 2159.01 (9) | 2166.84 (6) |

**Supporting Text for Table S2**

Structural analysis of calcined Li*x*–LLZTO (*x* = 0, 5, 10, 15, and 20) was performed with the Li and O occupancies fixed at 6.2 and 11.95 pfu, respectively. These values were chosen based on the quantitative analyses presented in Figures S2-3.

**Table S3**. Quantitative analysis of La_2_Zr_2_O_7_ impurity in the sintered Li*x*–LLZTO (*x* = 0, 5, 10, 15, and 20).

| Sintered Li*x*–LLZTO powders | La_2_Zr_2_O_7_ [*wt.*%] |
| --- | --- |
| 0 | 0.93 |
| 5 | 0.48 |
| 10 | – |
| 15 | – |
| 20 | – |

| Sintered Li*x*–LLZTO pellets | La_2_Zr_2_O_7_ [*wt.*%] |
| --- | --- |
| 0 | 0.78 |
| 5 | 2.02 |
| 10 | 0.64 |
| 15 | 0.87 |
| 20 | 1.43 |

| Sintered Li*x*–LLZTO polished pellets | La_2_Zr_2_O_7_ [*wt.*%] |
| --- | --- |
| 0 | 0.70 |
| 5 | 0.48 |
| 10 | 1.01 |
| 15 | 0.95 |
| 20 | 0.69 |

**Table S4**. Structural parameters derived from Rietveld refinement of ND patterns of sintered Li*x*–LLZTO (*x* = 0, 5, 10, 15, and 20).

| Sintered Li0–LLZTO | | | | | |
| --- | --- | --- | --- | --- | --- |
| Site | *x* | *y* | *z* | B_iso_ | *Occ.* |
| La | 0.125 | 0 | 0 | 0.45 (3) | 1 |
| Zr | 0 | 0 | 0 | 0.45 (4) | 0.75 |
| Ta | 0 | 0 | 0 | 0.45 (4) | 0.25 |
| Li1 | 0.375 | 0 | 0.25 | 2.34 (19) | 0.551 (12) |
| Li2 | 0.6880 (11) | 0.5833 (10) | 0.0987 (10) | 2.34 (19) | 0.400 (3) |
| O | 0.2813 (1) | 0.1016 (1) | 0.1966 (1) | 0.80 (3) | 0.9958 |

| Sintered Li5–LLZTO | | | | | |
| --- | --- | --- | --- | --- | --- |
| Site | *x* | *y* | *z* | B_iso_ | *Occ.* |
| La | 0.125 | 0 | 0 | 0.62 (2) | 1 |
| Zr | 0 | 0 | 0 | 0.57 (3) | 0.75 |
| Ta | 0 | 0 | 0 | 0.57 (3) | 0.25 |
| Li1 | 0.375 | 0 | 0.25 | 2.60 (14) | 0.483 (8) |
| Li2 | 0.6888 (7) | 0.5814 (7) | 0.0987 (7) | 2.60 (14) | 0.417 (2) |
| O | 0.2815 (1) | 0.1014 (1) | 0.1966 (1) | 0.93 (2) | 0.9967 |

| Sintered Li10–LLZTO | | | | | |
| --- | --- | --- | --- | --- | --- |
| Site | *x* | *y* | *z* | B_iso_ | *Occ.* |
| La | 0.125 | 0 | 0 | 0.53 (2) | 1 |
| Zr | 0 | 0 | 0 | 0.43 (3) | 0.75 |
| Ta | 0 | 0 | 0 | 0.43 (3) | 0.25 |
| Li1 | 0.375 | 0 | 0.25 | 2.05 (13) | 0.437 (8) |
| Li2 | 0.6892 (6) | 0.5782 (6) | 0.0971 (5) | 2.05 (13) | 0.416 (2) |
| O | 0.2815 (1) | 0.1013 (1) | 0.1967 (1) | 0.87 (2) | 0.9992 |

| Sintered Li15–LLZTO | | | | | |
| --- | --- | --- | --- | --- | --- |
| Site | *x* | *y* | *z* | B_iso_ | *Occ.* |
| La | 0.125 | 0 | 0 | 0.53 (2) | 1 |
| Zr | 0 | 0 | 0 | 0.43 (3) | 0.75 |
| Ta | 0 | 0 | 0 | 0.43 (3) | 0.25 |
| Li1 | 0.375 | 0 | 0.25 | 2.05 (13) | 0.437 (8) |
| Li2 | 0.6892 (6) | 0.5783 (6) | 0.0971 (6) | 2.05 (13) | 0.432 (2) |
| O | 0.2815 (1) | 0.1013 (1) | 0.1967 (1) | 0.87 (2) | 0.9983 |

| Sintered Li20–LLZTO | | | | | |
| --- | --- | --- | --- | --- | --- |
| Site | *x* | *y* | *z* | B_iso_ | *Occ.* |
| La | 0.125 | 0 | 0 | 0.56 (2) | 1 |
| Zr | 0 | 0 | 0 | 0.53 (3) | 0.75 |
| Ta | 0 | 0 | 0 | 0.53 (3) | 0.25 |
| Li1 | 0.375 | 0 | 0.25 | 2.01 (13) | 0.454 (8) |
| Li2 | 0.6887 (7) | 0.5780 (6) | 0.0972 (6) | 2.01 (13) | 0.416 (2) |
| O | 0.2815 (1) | 0.1013 (1) | 0.1967 (1) | 0.94 (2) | 0.9988 |

|  | Li0 | Li5 | Li10 | Li15 | Li20 |
| --- | --- | --- | --- | --- | --- |
| R_p_ (%) | 3.73 | 3.55 | 4.11 | 4.08 | 4.01 |
| R_wp_ (%) | 5.17 | 4.83 | 5.76 | 5.63 | 5.39 |
| R_F_ (%) | 3.30 | 1.96 | 1.32 | 1.35 | 1.45 |
| R_I_ (%) | 3.77 | 2.48 | 2.09 | 2.17 | 2.19 |
| a (Å) | 12.9402 (1) | 12.9401 (1) | 12.9370 (1) | 12.9371 (1) | 12.9385 (1) |
| Vol. (Å^3^) | 2166.83 (2) | 2167.19 (2) | 2165.23 (2) | 2165.24 (2) | 2165.97 |

**Supporting Text for Table S4**

Prior to the structural refinement of sintered Li*x*–LLZTO, the Li and O occupancies were quantitatively evaluated, and the resulting values were applied to the refinement of each composition, as shown in Figure S11.

**Table S5**. Inductively coupled plasma optical emission sepctroscopy (ICP-OES) elemental compositions of calcined Li*x*-LLZTO powders (*x* = 0, 5, 10, 15, and 20).

| Calcined Li*x*–LLZTO | Li | La | Zr | Ta |
| --- | --- | --- | --- | --- |
| 0 | 7.42 | 3 | 1.53 | 0.5 |
| 5 | 8.42 | 3 | 1.55 | 0.5 |
| 10 | 8.84 | 3 | 1.55 | 0.5 |
| 15 | 8.95 | 3 | 1.55 | 0.5 |
| 20 | 9.07 | 3 | 1.54 | 0.5 |

**Supporting Text for Table S5**

Inductively coupled plasma based techniques are widely used for elemental analysis; however, they exhibit intrinsic limitations when applied to the quantitative determination of lithium, which is a light element. In both ICP-OES and inductively coupled plasma mass spectrometry, lithium quantification can be affected by matrix effects, low signal intensity, and potential lithium loss during the dissolution process, which can limit absolute accuracy.

Despite these limitations, ICP-OES analysis was performed on the calcined Li*x*-LLZTO powders (*x* = 0, 5, 10, 15, and 20), which were subsequently used for the fabrication of both pellets and bedding powders. As summarized in Table S5, although the absolute lithium contents do not perfectly match the ideal stoichiometric values, the measured lithium concentration exhibits a clear and systematic increase with increasing nominal lithium excess. This trend confirms that the designed lithium excess in the precursor compositions is consistently reflected in the starting materials, providing qualitative to semi-quantitative validation of the compositional design prior to sintering.

**Table S6**. Quantitative analysis of impurity phases derived from Rietveld refinement of XRD patterns of powders from sintered Li*y*/Li0–LLZTO pulverized pellets (*y* = 0, 5, 10, 15, and 20).

| Sintered Li*y*/Li0–LLZTO powder | La_2_Zr_2_O_7_ [*wt.*%] |
| --- | --- |
| 0 | 3.03 |
| 5 | 2.70 |
| 10 | 1.15 |
| 15 | 0.77 |
| 20 | – |

**Table S7**. Refined structural parameters derived from Rietveld refinement of ND patterns of sintered Li*y*/Li0–LLZTO samples (*y* = 0, 5, 10, 15, and 20).

| Sintered Li0/Li0–LLZTO | | | | | |
| --- | --- | --- | --- | --- | --- |
| Site | *x* | *y* | *z* | B_iso_ | *Occ.* |
| La | 0.125 | 0 | 0 | 0.45 (3) | 1 |
| Zr | 0 | 0 | 0 | 0.45 (4) | 0.75 |
| Ta | 0 | 0 | 0 | 0.45 (4) | 0.25 |
| Li1 | 0.375 | 0 | 0.25 | 2.34 (19) | 0.551 (12) |
| Li2 | 0.6880 (11) | 0.5833 (10) | 0.0987 (10) | 2.34 (19) | 0.400 (3) |
| O | 0.2813 (1) | 0.1016 (1) | 0.1966 (1) | 0.80 (3) | 0.9958 |

| Sintered Li5/Li0–LLZTO | | | | | |
| --- | --- | --- | --- | --- | --- |
| Site | *x* | *y* | *z* | B_iso_ | *Occ.* |
| La | 0.125 | 0 | 0 | 0.45 (3) | 1 |
| Zr | 0 | 0 | 0 | 0.46 (4) | 0.75 |
| Ta | 0 | 0 | 0 | 0.46 (4) | 0.25 |
| Li1 | 0.375 | 0 | 0.25 | 2.52 (18) | 0.527 (12) |
| Li2 | 0.6876 (10) | 0.5819 (10) | 0.0981 (8) | 2.52 (18) | 0.410 (3) |
| O | 0.2814 (1) | 0.1015 (1) | 0.1966 (1) | 0.81 (2) | 0.9996 |

| Sintered Li10/Li0–LLZTO | | | | | |
| --- | --- | --- | --- | --- | --- |
| Site | *x* | *y* | *z* | B_iso_ | *Occ.* |
| La | 0.125 | 0 | 0 | 0.37 (2) | 1 |
| Zr | 0 | 0 | 0 | 0.35 (3) | 0.75 |
| Ta | 0 | 0 | 0 | 0.35 (3) | 0.25 |
| Li1 | 0.375 | 0 | 0.25 | 2.43 (15) | 0.497 (12) |
| Li2 | 0.6889 (8) | 0.5792 (7) | 0.0964 (7) | 2.43 (15) | 0.447 (3) |
| O | 0.2815 (1) | 0.1013 (1) | 0.1967 (1) | 0.77 (2) | 0.9983 |

| Sintered Li15/Li0–LLZTO | | | | | |
| --- | --- | --- | --- | --- | --- |
| Site | *x* | *y* | *z* | B_iso_ | *Occ.* |
| La | 0.125 | 0 | 0 | 0.31 (2) | 1 |
| Zr | 0 | 0 | 0 | 0.28 (3) | 0.75 |
| Ta | 0 | 0 | 0 | 0.28 (3) | 0.25 |
| Li1 | 0.375 | 0 | 0.25 | 2.08 (15) | 0.447 (12) |
| Li2 | 0.6877 (7) | 0.5787 (7) | 0.0969 (7) | 2.08 (15) | 0.448 (3) |
| O | 0.2815 (1) | 0.1012 (1) | 0.1969 (1) | 0.71 (2) | 0.9992 |

| Sintered Li20/Li0–LLZTO | | | | | |
| --- | --- | --- | --- | --- | --- |
| Site | *x* | *y* | *z* | B_iso_ | *Occ.* |
| La | 0.125 | 0 | 0 | 0.26 (2) | 1 |
| Zr | 0 | 0 | 0 | 0.25 (3) | 0.75 |
| Ta | 0 | 0 | 0 | 0.25 (3) | 0.25 |
| Li1 | 0.375 | 0 | 0.25 | 2.25 (15) | 0.503 (12) |
| Li2 | 0.6881 (8) | 0.5780 (7) | 0.0964 (7) | 2.25 (15) | 0.49 (3) |
| O | 0.2816 (1) | 0.1012 (1) | 0.1967 (1) | 0.70 (2) | 0.9975 |

|  | Li0/Li0 | Li5/Li0 | Li10/Li0 | Li15/Li0 | Li20/Li0 |
| --- | --- | --- | --- | --- | --- |
| R_p_ (%) | 3.73 | 3.91 | 4.09 | 4.18 | 4.35 |
| R_wp_ (%) | 5.17 | 5.39 | 5.44 | 5.57 | 5.83 |
| R_F_ (%) | 3.30 | 2.72 | 1.81 | 1.84 | 1.78 |
| R_I_ (%) | 3.77 | 3.19 | 2.57 | 2.70 | 2.60 |
| *a* (Å) | 12.9402 (1) | 12.9382 (1) | 12.9349 (1) | 12.9337 (1) | 12.9340 (1) |
| *Vol.* (Å^3^) | 2166.83 (2) | 2165.81 (2) | 2164.14 (2) | 2163.54 (2) | 2163.69 (2) |

**Supporting Text for Table S7**

Prior to conducting structural refinement for the sintered Li*y*/Li0–LLZTO, the Li and O occupancies were quantified, and the determined values were applied to each composition during the refinement procedure (see Figure S17).

**Table S8.** Summary of reported lithium compositions and corresponding crystal structures for doped LLZO garnet electrolytes from the literature, highlighting the critical lithium contents associated with the cubic-to-tetragonal phase transition.

| Dopant | Site | x | Li composition | Reported phase | Reference |
| --- | --- | --- | --- | --- | --- |
| Ga | Li | 0.10 | 6.70 | Cubic + tetragonal | [49] |
|  |  | 0.15 | 6.55 | Cubic |  |
|  |  | 0.20 | 6.40 | Cubic |  |
|  |  | 0.25 | 6.25 | Cubic |  |
|  |  | 0.30 | 6.10 | Cubic |  |
|  |  | 0.35 | 5.95 | Cubic |  |
|  |  | 0.40 | 5.80 | Cubic |  |
| Ge | Li | 0.05 | 6.80 | Cubic + tetragonal | [50] |
|  |  | 0.10 | 6.60 | Cubic |  |
|  |  | 0.15 | 6.40 | Cubic + impurity |  |
|  |  | 0.30 | 5.80 | Impurity-dominated |  |
|  |  | 0.50 | 5.00 | Impurity-dominated |  |
| Ce | La | 0.00 | 7.00 | Tetragonal | [51] |
|  |  | 0.20 | 6.80 | Cubic |  |
|  |  | 0.40 | 6.60 | Cubic |  |
|  |  | 0.60 | 6.40 | Cubic |  |
| Ce | Zr | 0.00 | 7.00 | Tetragonal | [52] |
|  |  | 0.125 | 7.00 | Tetragonal |  |
|  |  | 0.25 | 7.00 | Tetragonal |  |
|  |  | > 0.25 | 7.00 | Cubic |  |
| Mo | Zr | 0.10 | 6.80 | Cubic + tetragonal | [53] |
|  |  | 0.20 | 6.60 | Cubic + tetragonal |  |
|  |  | 0.25 | 6.50 | Cubic |  |
|  |  | 0.30 | 6.40 | Cubic + impurity |  |
|  |  | 0.40 | 6.20 | Cubic + impurity |  |
| Nb | Zr | 0.00 | 7.00 | Tetragonal | [54] |
|  |  | 0.25 | 6.75 | Cubic + tetragonal |  |
|  |  | 0.375 | 6.625 | Cubic |  |
|  |  | 0.50 | 6.50 | Cubic |  |
|  |  | 0.75 | 6.25 | Cubic |  |
| Nd | Zr | 0.05 | 7.05 | Cubic + tetragonal | [55] |
|  |  | 0.10 | 7.10 | Cubic + tetragonal |  |
|  |  | 0.15 | 7.15 | Cubic + tetragonal |  |
|  |  | 0.20 | 7.20 | Cubic + tetragonal |  |
| Ta | Zr | 0.00 | 7.00 | Tetragonal | [56] |
|  |  | 0.25 | 6.75 | Cubic + tetragonal |  |
|  |  | 0.50 | 6.50 | Cubic |  |
|  |  | 0.75 | 6.25 | Cubic |  |
|  |  | 1.00 | 6.00 | Cubic |  |

**Supporting Text for Table S8**

In the literature, the stability of the cubic garnet phase has commonly been discussed primarily in terms of dopant type, dopant concentration, and nominal lithium composition. Within this framework, critical lithium contents for the cubic-to-tetragonal phase transition have been reported for various doped LLZO systems, including Ta-doped compositions, as summarized in Table S8. However, these discussions generally do not explicitly consider oxygen vacancy formation and the accompanying lithium loss that inevitably occur during high-temperature sintering.

If oxygen vacancy formation and the associated lithium depletion are not taken into account, the lithium content actually retained within the garnet lattice can deviate substantially from the nominal composition. Under such conditions, the lithium content required to stabilize the cubic phase cannot be defined without explicitly considering defect chemistry. Consequently, cubic phase stabilization reported in previous studies may originate either from dopant-induced changes in lithium content or from lithium depletion associated with additional oxygen vacancy formation during sintering. Because the actual lithium content within the garnet lattice has not been quantitatively resolved in these studies, the origin of cubic phase stabilization remains ambiguous.

In this work, we explicitly address this limitation by quantitatively analyzing Li and O vacancies at both the calcined and sintered stages and correlating these vacancy concentrations with the resulting crystal phases. Through this approach, we identify the ranges of Li and O vacancy concentrations associated with the formation of the cubic garnet phase. As a representative example, previous studies on Ta-doped LLZO have reported the cubic-to-tetragonal phase boundary at approximately 6.5 Li per formula unit, implicitly assuming that the targeted dopant concentration and nominal lithium content directly govern phase stability. However, using a conventional sintering process, we demonstrate that even in Ta0.5-doped LLZO synthesized with excess lithium, intrinsic Li and O vacancies persist while the cubic phase remains stabilized. This result indicates that the reported phase boundary at 6.5 Li per formula unit does not uniquely define cubic phase stability and highlights an inherent ambiguity in the literature regarding the respective roles of dopant concentration and lithium content.

**Table S9**. Calculated Li occupancies in the chemical formula, converted from fractional site occupancies, for the Li1 (24d) and Li2 (96d) sites, and Li–Li bonding environments (bond lengths and bond angles) of Li*y*/Li0–LLZTO (*y* = 0, 5, 10, 15, and 20), derived from Rietveld refinement of ND patterns.

| Li*y*/Li0–LLZTO | Li1 (24d) | Li2 (96h) |
| --- | --- | --- |
| 0 | 1.653 | 4.797 |
| 5 | 1.582 | 4.918 |
| 10 | 1.492 | 5.358 |
| 15 | 1.430 | 5.370 |
| 20 | 1.509 | 5.391 |

| Li*y*/Li0–LLZTO | Li1–Li1 [Å] | Li1–Li2 [Å] | Li2–Li2 [Å] |
| --- | --- | --- | --- |
| 0 | 3.96212 (3) | 1.602 (14) | 0.780 (3) |
| 5 | 3.96150 (3) | 1.604 (12) | 0.780 (3) |
| 10 | 3.96048 (0) | 1.591 (10) | 0.811 (19) |
| 15 | 3.96011 (0) | 1.606 (10) | 0.786 (19) |
| 20 | 3.96020 (3) | 1.602 (10) | 0.796 (19) |

| Li*y*/Li0–LLZTO | Li1–Li2–Li1 [deg.] | Li2–Li1–Li2 [deg.] |
| --- | --- | --- |
| 0 | 168.6(9) | 96.5(3) |
| 5 | 168.9(8) | 96.9(3) |
| 10 | 170.4(7) | 98.0(3) |
| 15 | 169.8(6) | 98.0(3) |
| 20 | 170.1(7) | 98.3(3) |

**Table S10**. Detailed structural information derived from Rietveld refinement of ND patterns associated with lithium in Li*x*–LLZTO (*x* = 0, 5, 10, 15, and 20).

| Li*x*–LLZTO | Li1–Li1 [Å] | Li1–Li2 [Å] | Li2–Li2 [Å] |
| --- | --- | --- | --- |
| 0 | 3.96212 (3) | 1.602 (14) | 0.780 (3) |
| 5 | 3.96233 (3) | 1.605 (10) | 0.775 (19) |
| 10 | 3.96114 (3) | 1.609 (7) | 0.772 (14) |
| 15 | 3.96115 (3) | 1.601 (7) | 0.789 (14) |
| 20 | 3.96159 (3) | 1.606 (9) | 0.782 (16) |

| Li*x*–LLZTO | Li1–Li2–Li1 [deg.] | Li2–Li1–Li2 [deg.] |
| --- | --- | --- |
| 0 | 168.6 (9) | 96.5 (3) |
| 5 | 170.0 (6) | 97.1 (3) |
| 10 | 171.0 (5) | 98.2 (2) |
| 15 | 171.0 (5) | 98.2 (2) |
| 20 | 170.7 (6) | 98.2 (2) |

**Table S11**. Calculated Li occupancies in the chemical formula, converted from fractional site occupancies, for the Li1 (24d) and Li2 (96d) sites in Li*x*–LLZTO (*x* = 0, 5, 10, 15, and 20), derived from Rietveld refinement of ND patterns.

| Li*x*–LLZTO | Li1 (24d) | Li2 (96h) |
| --- | --- | --- |
| 0 | 1.653 | 4.797 |
| 5 | 1.448 | 5.002 |
| 10 | 1.311 | 4.989 |
| 15 | 1.311 | 5.189 |
| 20 | 1.362 | 4.988 |

**Table S12**. Detailed structural information derived from Rietveld refinement of ND patterns for Li20/Li0–LLZTO incorporating defect models with La and Zr deficiency to account for charge compensation.

| Li20/Li0–LLZTO | | | | | |
| --- | --- | --- | --- | --- | --- |
| Site | *x* | *y* | *z* | B_iso_ | *Occ.* |
| La | 0.125 | 0 | 0 | 0.20 (2) | 0.980 |
| Zr | 0 | 0 | 0 | 0.13 (3) | 0.719 |
| Ta | 0 | 0 | 0 | 0.13 (3) | 0.250 |
| Li1 | 0.375 | 0 | 0.25 | 2.33 (15) | 0.502 (8) |
| Li2 | 0.6886 (8) | 0.5786 (7) | 0.0962 (7) | 2.33 (15) | 0.449 (2) |
| O | 0.2816 (1) | 0.1013 (1) | 0.1968 (1) | 0.76 (2) | 0.9988 |

|  |  | Li1 (24d) | Li2 (96h) |
| --- | --- | --- | --- |
| R_p_ (%) | 4.33 | 1.507 | 5.393 |
| R_wp_ (%) | 5.81 |  |  |
| R_F_ (%) | 1.73 |  |  |
| R_I_ (%) | 2.58 |  |  |
| a (Å) | 12.9340 (1) |  |  |
| Vol. (Å^3^) | 2163.69 (2) |  |  |

|  | Li–Li repulsion parameter | | Li-loop angle parameter | |
| --- | --- | --- | --- | --- |
|  | Li1–Li1 [Å] | Li2–Li2 [Å] | Li1–Li2–Li1 [deg.] | Li2–Li1–Li2 [deg.] |
| Li20/Li0 | 3.96020 (0) | 0.809 (19) | 170.2 (7) | 98.2 (3) |

**Supporting Text for Table S12**

During the Li/O-rich atmosphere sintering process, the reduction of lithium vacancies and oxygen defects contributes to the relaxation of internal strain, enabling stabilization of the cubic structure even when the lithium content exceeds 6.5 pfu. In addition to this structural relaxation mechanism, further analysis was conducted to evaluate the possibility of charge compensation through metal deficiencies.

At elevated sintering temperature, Li–O evaporation may lead to the formation of impurity phases such as La_2_Zr_2_O_7_, during which La and Zr in the garnet structure can be lost in a 1:1 ratio. To assess this possibility, a defect model was constructed based on the quantified lithium and oxygen contents, where the occupancies were fixed at Li = 6.9 and O = 11.985. Under these conditions, charge neutrality could be achieved by introducing La and Zr deficiencies of 0.071 pfu, each in equal proportion.

Rietveld refinement using this defect model yielded reasonable fitting parameters, indicating that La- and Zr-deficient LLZTO is structurally plausible. This result suggests that metal-deficiency-based charge compensation is a viable mechanism in lithium-rich garnet systems.

**Table S13**. Detailed structural information derived from Rietveld refinement of ND patterns of Li20/Li10–LLZTO synthesized using a ^7^Li precursor.

| Li20/Li10–LLZTO | | | | | |
| --- | --- | --- | --- | --- | --- |
| Site | *x* | *y* | *z* | B_iso_ | *Occ.* |
| La | 0.125 | 0 | 0 | 0.28 (2) | 1 |
| Zr | 0 | 0 | 0 | 0.34 (3) | 0.75 |
| Ta | 0 | 0 | 0 | 0.34 (3) | 0.25 |
| Li1 | 0.375 | 0 | 0.25 | 1.97 (15) | 0.495 (8) |
| Li2 | 0.6873 (8) | 0.5768 (7) | 0.0977 (7) | 1.97 (15) | 0.426 (2) |
| O | 0.2814 (1) | 0.1012 (1) | 0.1966 (1) | 0.77 (2) | 0.9992 |

|  |  | Li1 (24d) | Li2 (96h) |
| --- | --- | --- | --- |
| R_p_ (%) | 4.56 | 1.484 | 5.116 |
| R_wp_ (%) | 6.00 |  |  |
| R_F_ (%) | 1.61 |  |  |
| R_I_ (%) | 2.65 |  |  |
| a (Å) | 12.9338 (1) |  |  |
| Vol. (Å^3^) | 2163.61 (2) |  |  |

|  | Li–Li repulsion parameter | | Li-loop angle parameter | |
| --- | --- | --- | --- | --- |
|  | Li1–Li1 [Å] | Li2–Li2 [Å] | Li1–Li2–Li1 [deg.] | Li2–Li1–Li2 [deg.] |
| Li20/Li10 | 3.96015 (3) | 0.752 (19) | 170.1 (7) | 98.5 (3) |

**References**

[1] N. Bernstein, M. Johannes, K. Hoang, *Phys. Rev. Lett.* **2012**, 109, 205702.

[2] S.-K. Jung, H. Gwon, H. Kim, G. Yoon, D. Shin, J. Hong, C. Jung, J.-S. Kim, *Nat. Commun.* **2022**, 13, 7638.

[3] X. Huang, Y. Lu, H. Guo, Z. Song, T. Xiu, M. E. Badding, Z. Wen, *ACS Appl. Energy Mater.* **2018**, 1, 5355.

[4] F. M. Pesci, R. H. Brugge, A. O. Hekselman, A. Cavallaro, R. J. Chater, A. Aguadero, *J. Mater. Chem.* **2018**, 6, 19817.

[5] S. Chen, Z. Nie, F. Tian, L. Nie, R. Wei, J. Yu, T. Gao, Z. Sun, N. Yang, W. Liu, *Adv. Funct. Mater.* **2022**, 32, 2113318.

[6] T. Dussart, N. Rividi, M. Fialin, G. Toussaint, P. Stevens, C. Laberty-Robert, *J. Electrochem. Soc.* **2021**, 168, 120550.

[7] H. Guo, J. Su, W. Zha, T. Xiu, Z. Song, M. E. Badding, J. Jin, Z. Wen, *J. Alloys Compd.* **2021**, 856, 157222.

[8] S. P. Kammampata, H. Yamada, T. Ito, R. Paul, V. Thangadurai, *J. Mater. Chem.* **2020**, 8, 2581.

[9] Y. Zhou, X. Li, Y. Yang, X. Huang, B. Tian, *ACS Appl. Energy Mater.* **2022**, 5, 13817.

[10] Y. Song, L. Yang, W. Zhao, Z. Wang, Y. Zhao, Z. Wang, Q. Zhao, H. Liu, F. Pan, *Adv. Energy Mater.* **2019**, 9, 1900671.

[11] Y. Lu, X. Huang, Y. Ruan, Q. Wang, R. Kun, J. Yang, Z. Wen, *J. Mater. Chem.* **2018**, 6, 18853.

[12] M. Stockham, B. Dong, M. James, Y. Li, Y. Ding, P. Slater, *Dalton Transactions* **2021**, 50, 2364.

[13] C. Zheng, Y. Ruan, J. Su, Z. Song, T. Xiu, J. Jin, M. E. Badding, Z. Wen, *Chem. Eng. J.* **2021**, 411, 128508.

[14] R. Wei, Y. Zhang, J. Yu, X. Zhang, Y. Zhang, T. Gao, Y. Yu, J. Zhao, W. Liu, *ACS Appl. Mater. Interfaces* **2024**, 16, 55198.

[15] R.-H. Lee, S.-H. Lee, *Journal of Energy Storage* **2023**, 72, 107421.

[16] Y. Song, L. Yang, L. Tao, Q. Zhao, Z. Wang, Y. Cui, H. Liu, Y. Lin, F. Pan, *J. Mater. Chem.* **2019**, 7, 22898.

[17] R. H. Basappa, T. Ito, T. Morimura, R. Bekarevich, K. Mitsuishi, H. Yamada, *J. Power Sources* **2017**, 363, 145.

[18] X. Tao, L. Yang, J. Liu, Z. Zang, P. Zeng, C. Zou, L. Yi, X. Chen, X. Liu, X. Wang, *J. Alloys Compd.* **2023**, 937, 168380.

[19] S. Patra, J. Narayanasamy, S. Chakravarty, R. Murugan, *ACS Appl. Energy Mater.* **2020**, 3, 2737.

[20] M. S. Jo, S. W. Kim, S. E. Lee, H. C. Lee, *Journal of the Korean Ceramic Society* **2025**, 62, 281.

[21] J.-W. Sim, R.-H. Lee, H.-K. Kim, J. K. Lee, J. R. Yoon, S.-H. Lee, *Chem. Mater.* **2023**, 35, 6538.

[22] W. Ji, B. Luo, Q. Wang, G. Yu, Z. Liu, Z. Zhao, R. Zhao, S. Wang, X. Wang, B. Zhang, *Adv. Energy Mater.* **2023**, 13, 2300165.

[23] A. Sharafi, C. G. Haslam, R. D. Kerns, J. Wolfenstine, J. Sakamoto, *J. Mater. Chem.* **2017**, 5, 21491.

[24] C. Zheng, J. Su, Z. Song, T. Xiu, J. Jin, M. E. Badding, Z. Wen, *Mater. Today Energy* **2022**, 27, 101034.

[25] R. Dubey, J. Sastre, C. Cancellieri, F. Okur, A. Forster, L. Pompizii, A. Priebe, Y. E. Romanyuk, L. P. Jeurgens, M. V. Kovalenko, *Adv. Energy Mater.* **2021**, 11, 2102086.

[26] M. Müller, J. Schmieg, S. Dierickx, J. Joos, A. Weber, D. Gerthsen, E. Ivers-Tiffée, *ACS Appl. Mater. Interfaces* **2022**, 14, 14739.

[27] L. Yang, X. Tao, X. Huang, C. Zou, L. Yi, X. Chen, Z. Zang, Z. Luo, X. Wang, *ACS Appl. Mater. Interfaces* **2021**, 13, 56054.

[28] A. Sharafi, H. M. Meyer, J. Nanda, J. Wolfenstine, J. Sakamoto, *J. Power Sources* **2016**, 302, 135.

[29] W. S. Scheld, K. Kim, C. Schwab, A. C. Moy, S. K. Jiang, M. Mann, C. Dellen, Y. J. Sohn, S. Lobe, M. Ihrig, *Adv. Funct. Mater.* **2023**, 33, 2302939.

[30] X. Ma, Y. Xu, *Electrochim. Acta* **2022**, 409, 139986.

[31] C.-L. Tsai, V. Roddatis, C. V. Chandran, Q. Ma, S. Uhlenbruck, M. Bram, P. Heitjans, O. Guillon, *ACS Appl. Mater. Interfaces* **2016**, 8, 10617.

[32] X. Ma, Y. Xu, *Electrochim. Acta* **2023**, 441, 141789.

[33] J. Zhang, C. Wang, J. Fu, M. Ye, H. Zhai, J. Li, G. Tan, X. Tang, X. Sun, *Adv. Funct. Mater.* **2025**, 35, 2416229.

[34] Z. Qin, Y. Xie, X. Meng, D. Qian, C. Shan, D. Mao, G. He, Z. Zheng, L. Wan, Y. Huang, *Chem. Eng. J.* **2022**, 447, 137538.

[35] B.-Q. Xiong, Q. Nian, X. Zhao, Y. Chen, Y. Li, J. Jiang, S. Jiao, X. Zhan, X. Ren, *ACS Energy Lett.* **2022**, 8, 537.

[36] X. Wang, X. Yu, X. Wang, J. Chen, D. Wang, C. Dong, Z. Mao, *Carbon Neutralization* **2025**, 4, e185.

[37] J. Lou, G. Wang, Y. Xia, C. Liang, H. Huang, Y. Gan, X. Tao, J. Zhang, W. Zhang, *J. Power Sources* **2020**, 448, 227440.

[38] S. Yu, Z. Gong, M. Gao, J. Li, W. Xie, Y. Wei, D. Li, L. Yang, D. Chen, Y. Li, *Journal of Materials Science & Technology* **2025**, 206, 248.

[39] A. Cheng, X. He, R. Wang, B. Shan, K. Wang, K. Jiang, *Chem. Eng. J.* **2022**, 450, 138236.

[40] C. Ji, S. Zhou, L. Cai, Y. Yuan, X. Liu, P. Huang, X. Xiong, *Chem. Eng. J.* **2025**, 506, 160161.

[41] Z. Bi, W. Huang, S. Mu, W. Sun, N. Zhao, X. Guo, *Nano Energy* **2021**, 90, 106498.

[42] F. Zhu, W. Deng, B. Zhang, H. Wang, L. Xu, H. Liu, Z. Luo, G. Zou, H. Hou, X. Ji, *Nano Energy* **2023**, 111, 108416.

[43] F. Zhu, H. Liu, J. Huang, B. Zhang, B. Song, W. Deng, G. Zou, H. Hou, X. Ji, *Adv. Funct. Mater.* **2024**, 34, 2411737.

[44] H. Huo, Y. Chen, R. Li, N. Zhao, J. Luo, J. G. P. da Silva, R. Mücke, P. Kaghazchi, X. Guo, X. Sun, *Energy & environmental science* **2020**, 13, 127.

[45] Y. Hu, P. Li, S. Yu, S. Fu, Y. Liu, Y. Wei, D. Li, L. Yang, D. Chen, N. Wang, *ACS Appl. Energy Mater.* **2025**, 8, 2928.

[46] S. Zhang, M. Jia, S. Guo, N. S. Grundish, A.-M. Cao, Y. Li, *Nano Lett.* **2025**.

[47] A. Baniya, A. Gurung, J. Pokharel, K. Chen, R. Pathak, B. S. Lamsal, N. Ghimire, R. S. Bobba, S. I. Rahman, S. Mabrouk, *ACS Appl. Energy Mater.* **2022**, 5, 648.

[48] W. Huang, Z. Bi, N. Zhao, Q. Sun, X. Guo, *Chem. Eng. J.* **2021**, 424, 130423.

[49] J.-F. Wu, E.-Y. Chen, Y. Yu, L. Liu, Y. Wu, W. K. Pang, V. K. Peterson, X. Guo, *ACS Appl. Mater. Interfaces* **2017**, 9, 1542.

[50] R. Brugge, J. Kilner, A. Aguadero, *Solid State Ion.* **2019**, 337, 154.

[51] E. Rangasamy, J. Wolfenstine, J. Allen, J. Sakamoto, *J. Power Sources* **2013**, 230, 261.

[52] B. Dong, S. R. Yeandel, P. Goddard, P. R. Slater, *Chem. Mater.* **2019**, 32, 215.

[53] D. Rettenwander, A. Welzl, L. Cheng, J. r. Fleig, M. Musso, E. Suard, M. M. Doeff, G. n. J. Redhammer, G. Amthauer, *Inorganic chemistry* **2015**, 54, 10440.

[54] Y. Luo, J. Dong, Y. Yan, J. Liu, L. Wang, Z. a. Chen, H. Zhang, *Journal of Materials Science* **2025**, 1.

[55] S. Saran, Y. R. Eker, *Current Applied Physics* **2022**, 41, 1.

[56] T. Zhang, T. D. Christopher, S. Huang, Y. g. Liu, W. Gao, T. Sӧhnel, P. Cao, *Ceram. Int.* **2019**, 45, 20954.
